# The Privileged Life of a Theoretical Observer

**DOI:** 10.1007/s11207-022-02011-7

**Published:** 2022-07-26

**Authors:** Douglas Gough

**Affiliations:** 1 Institute of Astronomy, Madingley Road, Cambridge, CB3 0HA UK; 2 Department of Applied Mathematics and Theoretical Physics, Centre for Mathematical Sciences, Wilberforce Road, Cambridge, CB3 0WA UK

## Abstract

This is a summary of my scientific career, biased by my personal view of events and unashamedly concentrating on those aspects of some of the scientific developments to which I have contributed. A selective unbiased alternative has been written by Christensen-Dalsgaard and Thompson (A selective overview. In: Thompson, M.J., Christensen-Dalsgaard, J. (Eds.) *Stellar Astrophysical Fluid Dynamics*, Cambridge University Press, pp. 1 – 19, 2003), followed by some further remarks by Christensen-Dalsgaard (*Unsolved Problems in Stellar Physics: A Conference in Honour of Douglas Gough*, American Institute of Physics Conference Series, 948, xii, 2007).

## In the Beginning

I was born somewhat over a year into the Second World War. My mother had been evacuated from London to Worcester, and after my birth we went to Torquay in the south-west of England; then she returned with me to the East End of London to live with her parents. My father had been conscripted into the army, sent to the south of Greece where he was captured and made to march to a stalag in Germany, thence to a POW camp just outside Graz where he remained for the rest of the war. My mother knew that he must have been either captured or killed; it was a very long time before she was told that he was alive. Most of the prisoners were forced into building a dam, and many of them died in the winter’s cold. My father was fortunate by being a tailor, and stayed inside repairing prisoners’ clothes (and carrying out other, clandestine, tailoring activities). After the war he was first taken to a rehabilitation centre before returning to England and being allowed a short visit home. Thus, I was five years old when first we met. I remember not taking to him at first because he showed much more interest in my mother than in me; I preferred the soldier who had driven my father home, for, not knowing anybody in the family, he played games with me. The situation was exacerbated by my father’s evident disapproval of some of the ways that his son had been brought up, by my grandmother in particular, which I took to be a criticism of me. Hoping that my father was a hero, I asked him how many enemy soldiers he had killed. I was bitterly disappointed when he replied: “I hope none”, and perplexed at the time as to how it could have been a hope. It is probably because I didn’t know him in my most formative years that we never became really close; however, I did develop a great respect for his integrity. My mother loved me dearly, but she never understood how I think.

It was not until four years later that my brother, Kenneth, was born; my parents considerately waited for me to adjust to my father’s appearance in the family. Thus, Kenneth and I were each essentially only children; it was not until we were both adults that we communicated as equals.

My earliest memories are of London: the most vivid is being terrified of the (no doubt friendly) people in horrendous-looking gas masks sitting opposite me in the local air-raid shelter during enemy attacks. For me that was worse than the sound of the planes overhead, the rumble of the doodlebugs and the terminal explosions; the implications of the rubble that the previous day had been the house next door did not properly penetrate into my young brain. Instead I was fascinated by the enormous barrage balloon filling the small park near by, where it was securely tethered at ground level in daytime. I remember at the end of the war the enormous hole in the street’s tarmac outside our house, a consequence of a VE-Day celebratory bonfire. At night I slept in my grandparents’ parlour, in which was a piano that I longed to play. But my formidable English grandmother forbade me even to touch the keys, on the grounds that I might damage them! That must have been partly why there was always some tension between us. My Italian grandfather, Vincente (who fought for England and was decorated in the first world war, and only subsequently was granted British citizenship, tactfully dropped the second e from his name at the start of the second world war)^1^, was quite different: he was tremendously loving and entertaining, as were his brothers Franco and Bruno. The highlights of those days were when the three partly Italian families would gather together for a meal: whenever I see Mafia-looking men dressed in dark suits, heavy black overcoats and black fedoras I am always filled with anticipation of good fun.

My most enjoyable interludes were holidays visiting my mother’s sister, Winnie, and my favourite uncle, Arthur, in the outskirts of Coventry. Before they were married, Arthur had cycled the nearly 200 km from London dressed in his suit for his job interview at Courtaulds; he was a conscientious objector, and during the war remained in Coventry developing and maintaining equipment for manufacturing viscose fibres, such as nylon thread for parachutes. I remember the strong fine fabric, sickly yellow-green in colour, and reinforced with webbing, and I was curious how parachutes were folded to ensure that they would open when needed. I was reminded of it some sixty years later when I visited the balloon-making facility of the Tata Institute of Fundamental Research (TIFR) in Hyderabad, though then the materials were a little different. During those wartime visits to Coventry I played all day with my cousins, Vivien and Christopher, in the woods, occasionally intruding on a dairy farm near by. It was idyllic. Later, in London, I enjoyed exploring bombed buildings, climbing to the roofs of derelict factories for the fine views; of course, they were strictly out of bounds.

Soon after my father was demobbed, he and my mother rented two rooms and a kitchen on the top floor of an old bomb-damaged house. My father worked very hard, having returned to Hector Powell’s tailoring factory where he was a foreman, and where he had met my mother. During that time he also had his own tailor’s shop in Clapton, just north of Bethnal Green, which he opened with money that he had won playing bridge in the POW camp. Often he could afford only four hours sleep in a night. As trade grew he was able to resign from his factory employment, and devote himself entirely to his own business. In my younger days I used to enjoy going to his shop, and talking with the customers. Many of them were Teddy Boys, young bachelors with money to spend and with a taste for high-quality suits in Edwardian style, but otherwise ordinary nice people. They became the mainstay of my father’s business until the fashion for jeans brought bespoke tailoring almost to a close. There were also customers who, I was told, were famous. One was a virtuoso violinist who had his tailcoat made with the left sleeve longer than the right in order to make them appear to be of the same length when he was playing his violin. Then there was Coco the Clown, whose voluminous check suit was reinforced with webbing, rather like my uncle Arthur’s parachutes, onto which a rope could be hooked for swinging him safely high around the Big Top. My favourites were two brothers, Reggie and Ronnie (Kray), immaculately dressed and very friendly, especially Reggie who always gave me half-a-crown.^2^ At my tender age I knew that they must be kind because they were in the protection business, looking after old people in the neighbourhood: I was assured that if any vulnerable person was burgled, the brothers would ensure that it would never happen again. Only when I was older did I realise that the aged were not the only people the brothers ‘protected’, and that the powers of persuasion, though very effective, were not the most tactful. Later, as a teenager after I had learnt to drive, I would help my father by delivering suits that he had made for other tailors, most notably in Savile Row. That was always a little delicate because the shops there are tiny, with no rear entrance, so there was always the danger that my meeting with the proprietor would be in the presence of a customer who believed that his beloved suit had been made on the premises. Needless to say, the price charged to the customer was staggeringly greater than what my father received, partly because Savile-Row rents are so high, but mainly, I gather, because the clientele would not have appreciated the quality of their acquisitions had they been charged merely a fair market price.

## School Days, and Unlikely Happenings

I went to a small primary school, Shacklewell, some twenty minutes walk from home. On the day I started, at the age of five, I was taken by my mother, but after that I refused to be seen being mollycoddled, and insisted that I went alone. That gave me a modicum of freedom.

In November 1950, aged nine, I slipped on the pavement on my way home from school and badly fractured my left femur. I was taken to the children’s ward in the Metropolitan Hospital where I awoke with my leg in traction supported from on high, just as I had seen in comic strips. Three weeks later I was put in a plaster cast extending from my toes to my neck, rendering me unable to bend. At that hospital children were not allowed visitors: the administration erroneously believed that the trauma experienced by a child following the departure of a relative or friend outweighed the loneliness of not seeing them at all! I was bored, and somehow a message got through to my parents, who responded by sending me a newly acquired volume devoted to teaching mathematics and some of its practical applications (Sawyer, 1948). I read it from cover to cover, and by the time I was temporarily discharged from hospital the following April I had completed most of the tests at the ends of the chapters. That was my introduction to a subject that was to dominate the rest of my life.

In those days many pupils’ greatest incentive for learning was the consequent end-of-year examination. It didn’t work for everyone, particularly those who didn’t do well: they didn’t do well partly because they were not motivated, and they were not motivated because they perceived that they wouldn’t do well. But in my schools, at least, that was not the case for those ‘near the top’, who studied just hard enough to compete favourably with the others in the class, and no harder: one’s level of attainment was therefore determined solely by the perceived ability of one’s peers. Educationalists in England later encouraged teachers to concentrate on the average and below-average pupils, believing naively that the brightest would attain their optimal level independently of anything else. Laudable as that stance might be for the sake of fairness, from my experience it is obviously otherwise flawed. I was lucky enough to be near the top, and won entrance to Hackney Downs School, culturally mixed and arguably the better of the boys’ grammar schools within walking distance of my home.

**Figure 1:**
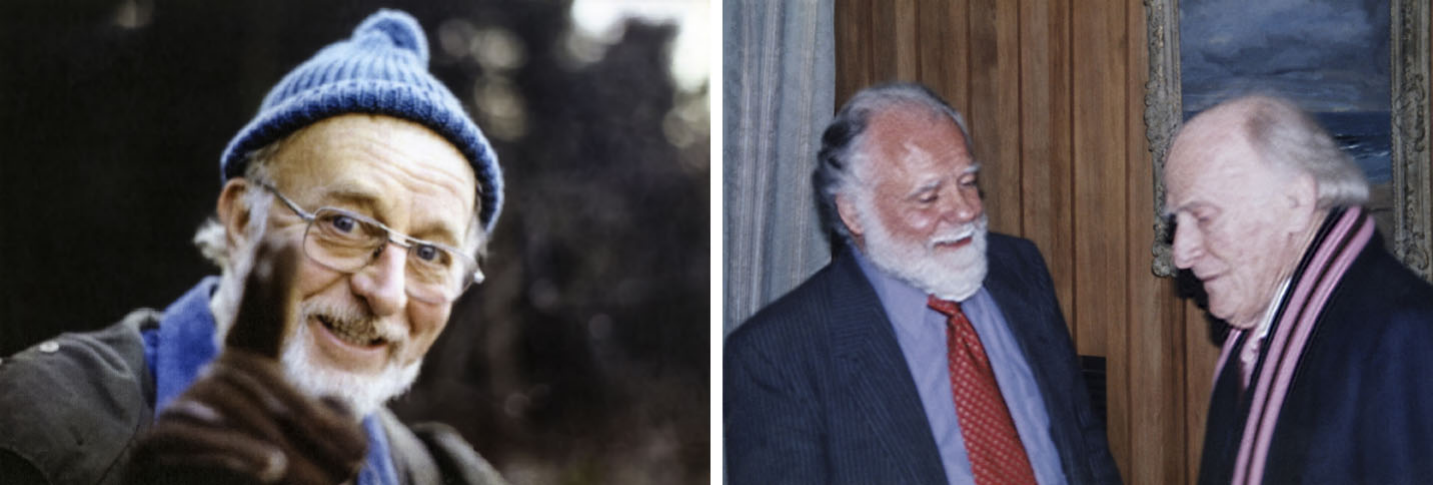
Left: Joe Brearley, Deputy Head of Hackney Downs School from 1953 until he retired in 1971. Right: A conversation with Yehudi Menuhin in 1999, two days before his last flight to Berlin.

My parents were strict (so I thought) on behaviour; I feared doing anything wrong and getting into trouble. So, on the whole, I was well behaved at home. Therefore school was a paradise for counterbalance. I didn’t believe in being naughty; that causes undue irritation, although I did disrupt a class once, which I’ll mention later. Otherwise, it was only what I thought were serious pranks that were worth bothering with, although looking back on them now they seem petty: climbing over the school wall onto the main-line railway track, usually to recover a ball; picking up (with the help of others) a master’s car and placing it gently between two trees front and back so that it couldn’t be driven away; smearing the steps up to the headmaster’s study after the close of school with nitrogen tri-iodide; raising a friend’s sister’s bra to the top of the flagpole and removing the halyard, rendering it necessary to erect a scaffold to restore propriety; impregnating the wooden floors of the chemistry laboratory with butyric acid – I didn’t realise how involatile it is – using so much that the putrid smell permeated the entire science wing for two months (causing me to regret having done it). Later, the butyric-acid episode was repeated by someone else, engendering Joe Brearley’s (Figure 1) furore, because this time the act was not original.

One of the strictly forbidden activities was to run along the corridors, rightly so because all the class rooms had heavy doors that opened both inwards and outwards, and to collide at speed with one would cause serious injury. Once, I was in a hurry and literally ran into Joseph (Gestapo, as we called him) Brearley (Watkins, 2008) as he stepped into the corridor. Joe (as later I was able to call him) was the deputy headmaster, the strictest of disciplinarians, meting out severe punishments to maintain order, yet being scrupulously fair. Terrified, I blurted out an excuse in my East-End argot. Instantly Joe went into attack by pointing out a grammatical error, against which I hurriedly reminded him how in spoken English certain words can be omitted when their implied presence would obviously be understood, uttering a hurriedly invented, necessarily inelegant, yet grammatically correct complete sentence in an attempt to reduce the severity of the impending punishment. To my surprise, Joe stopped in his tracks, turned around, and walked off, speechless. Some days later he sought me out, telling me that he had gone straight to the staff room to report on what had happened, and that the English department were still arguing as to whether I was right. The issue of punishment never arose; so far as I was concerned my literary invention was a complete success. I tell this anecdote because it epitomises the manner in which Joe influenced the character of the school: he put intellectual inventiveness above all else.

Honesty and loyalty were characteristics that Joe instilled. No miscreant would ever allow another to take his blame, and no schoolmaster would ever put a pupil in the position of having to reveal information that would incriminate another. I discovered later that such a code of ethics is not shared by at least some of those who consider themselves to be ‘upper class’. I suppose that explains the expression “Honour amongst thieves”.

Joe taught English and his favourite language, German. He had inspired many pupils of the school, including the future Nobel-Prize-winning playwright Harold Pinter, and the actors Steven Berkoff and Michael Caine amongst many, and he established the ethos of the school, emphasising integrity, hard work and hard play. The school was not outstanding academically, although it did send several pupils annually to colleges principally in London to study mainly lucrative practical subjects. However, we were the best amongst London schools in field athletics. I was not a good field athlete, but I did run cross country for the school, and I played rugby fives: in my final year I captained the London Grammar Schools team, and later I played for my Cambridge college and, occasionally, for the university. Fives courts are few and far between, so I also took up squash, which I played for the next four-and-a-half decades. Long after Joe and the other masters of his time had retired, the school went into decline. In 1994, Hackney Downs became the first state high school in the country to be fatally condemned.

One of the interesting aspects of Hackney Downs School was that we often had a visiting teacher on sabbatical leave from elsewhere. One such man was Ken Evans, senior mathematics master from Scotch College in Melbourne, Australia. It was one of his classes that I once disrupted. He announced that he was about to teach us probability theory. Behaving as a frequentist, he started by saying that if one were to toss a coin ten times it would be more likely to come down five heads and five tails than in any other combination. I playfully interjected, claiming that my belief was six tails followed by four heads. Ken ignored me and continued. Without another word, I took a coin and tossed it high in the air, announcing a tail after it fell. I tossed again: another tail. Then I tossed a third. By now the class were congregating around me, and after the fourth tail even Ken arrived to convince himself that I was not cheating. I felt strangely empowered as I had never experienced before, and rarely since, and had absolutely no doubt that my prophesy would come to pass. And indeed it did: I tossed two more tails, followed by four heads. In retrospect, I realise how impressive Ken was: he quietly apologised, saying he had muddled his schedule, and that he would teach probability in a few weeks time, in the meanwhile continuing with calculus. Needless to say, I have never even tried to repeat that performance. I have had other rare experiences that offer no ordinary explanation, and that have taught me always to maintain an open mind when I encounter people, my wife Rosanne especially, who have experienced ‘unbelievable’ happenings. There is a tendency among scientists to disbelieve what cannot be repeated; a non-repeatable phenomenon is difficult to address by scientific method. But that does not mean that it cannot happen.

As an aside, I relate one of the several ‘unbelievable’ happenings encountered by Rosanne, chosen because I experienced it too. About twenty years ago we went to a performance of, appropriately as it turned out, ‘A Midsummer Night’s Dream’ at the University of Colorado. Unusually, we had difficulty finding a place to park. I left Rosanne at the theatre, and eventually succeeded, parking rather more distantly than in the past. Rosanne was welcomed at the theatre door by a long-bearded man, about our age, who escorted her to her place, and told her in an apparently over-sincere manner how he really hoped that she would enjoy the performance. How odd! At the interval I went to buy coffee. On my return I found Rosanne with the very same man, who extraordinarily politely sought my permission to talk to her. He said that he would never forget her, because as a nurse she had looked after him so well when he was a young man in Wardenburg, the university’s medical centre, suffering from a rare disease. Rosanne then knew who he must be; bewildered, she and I briefly looked at each other, and then turned back to address the man: he had vanished! The auditorium was almost empty; he could not possibly have simply walked out of sight. After the performance as we returned to the car, we realised that it was parked outside Wardenburg, where the man with whom we had been talking had died more than three decades earlier.

Back to schooldays. When I was about 15 years old, the London Symphony Orchestra came to Hackney to expose young people to classical music. Every secondary-school pupil in the borough had the opportunity to attend, and since it involved missing a morning’s lessons I leapt at it. I found the experience absolutely wonderful. In the middle of the concert I was bowled over by a piece by Mendelssohn, probably the Hebrides Overture, and was especially enthusiastic in my applause. A rather ‘superior’ boy sitting next to me frowned and told me that such enthusiasm should be left until the end of the entire concert. How silly! That morning was perhaps the true dawn of my love for music, although I have not forgotten my unfulfilled childhood desire to play the piano. I then went frequently to concerts and operas whenever I had enough money, especially in summer to Henry Wood’s affordable Promenade Concerts at the century-old Royal Albert Hall: I recall an occasion during the fourth movement of the Pastoral Symphony being almost drowned by rain dripping on my head from the leaking roof,^3^ hearing thunder from both outside and in. Even Beethoven hadn’t foreseen such realism, I suspect. I recall especially the first performance outside the Soviet Union of Dmitri Shostakovich’s cello concerto at the Royal Festival Hall, played by Mstislav Rostropovich with the Leningrad Symphony Orchestra under Gennadi Rozhdestvensky in the presence of Shostakovich himself: the encore was the entire cadenza and final movement, as had been the case with the violin concerto. I enjoyed a performance of Beethoven’s violin concerto by Wolfgang Schneiderhan who then played the entire chaconne from JS Bach’s Partita No2 as an encore; and I was stunned by an ‘unknown’ soprano at the Covent Garden Opera at what I later learned from the newspapers was Joan Sutherland’s London debut. Living in a city like London has enormous cultural benefit. I used often to buy a cheap seat in the choir at the Royal Festival Hall, behind the orchestra and facing the conductor; one memorable occasion was when I found myself in the front row with my head immediately above the bell of a tuba, which was absolutely wonderful when the tuba wasn’t being played. A very moving occasion was a concert by the Hallé Orchestra under Sir John Barbirolli, perchance on the day that Ralph Vaughan Williams died; the death was announced by Barbirolli, who, before the advertised programme, conducted the Fantasia on a Theme of Thomas Tallis as a tribute, having requested that there should be no applause at the end: wonderfully pure silence after the strings gradually died down at the end had an enormous emotional impact. Subsequently, there was a memorial service at Westminster Abbey, at which my two favourite violinists, Yehudi Menuhin and David Oistrakh – poles apart in style – played Bach’s double concerto in D minor. I was extremely fortunate some four decades later to spend half-an-hour alone with Yehudi (Figure 1), kindly arranged by John Boyd, Master of Churchill College. We discussed humanism and music, during which Yehudi praised the world’s most inventive composers, J.S. Bach, Mozart and The Beatles, just two days before he visited Berlin to perform in a concert, where, most sadly, he died.

## Towards Cambridge

In my teens I always sought some kind of paid employment, from newspaper-delivery rounds to working as a laboratory assistant in my school, helping to prepare demonstrations and cleaning up the apparatus afterwards. It was during that time that I illicitly put some phosphorus in a deflagrating spoon and gingerly held it up into the pilot light of a geyser in the chemistry laboratory. The almost explosive ignition ejected the burning material that became caught in the sleeve of my lab coat, burning my right wrist. So severe was the burn that it didn’t hurt: I felt nothing, so I thought nothing of it. However, the other lab assistants insisted that I report it, whereupon I was rushed to hospital where I was detained for more than a week for fear of losing the use of my hand. The scar remains in remembrance. A more successful event followed: There was a strange common belief that in Australia the bathtub vortex flows in the opposite direction to those in the northern hemisphere. A simple angular-momentum-conservation argument rendered that highly unlikely, yet I could not convince the believers. The sinks in the school’s laboratories, though not themselves axisymmetric, were plugged by tall circularly symmetrical cylindrical stoppers which could be removed without inducing circulation in the water they restrained. So one Friday evening I filled every sink in all the laboratories, and asked the caretaker not to disturb them over the weekend and to kindly let me in before school opened on Monday morning. Then, I carefully removed the plugs and observed the ensuing vortices. There was no statistically significant evidence for the direction of the Earth’s rotation. But there was a very significant bias towards the direction of the bend in the drainpipe under the bench. Interestingly, I was one day to become a member of the university department headed by George Batchelor, who has contributed much to the theory of vortices (Batchelor, 1967) required for understanding the draining of fluids from sinks (e.g. Klimenko, 1998).

Later, I spent an entire summer holiday working for the Mond Nickel Company, helping a scientist with his experimental investigation into the surface properties of rhodium. I was actually able to make useful suggestions, and was sometimes allowed to pursue them myself. I remember the exhilaration of partaking in a discovery, albeit minor. It made me want a career in research, presumably industrial – I was quite unaware that academic research exists. However, I did find the slow progress of the experimental procedure somewhat frustrating, particularly because repeatability was hardly better in this professional laboratory than what I had experienced at school. So I resolved to be a theorist, not realising that repeated calculations even by mature scientists also don’t necessarily come up with the same answer every time!

I recognised that what I needed to study at university was mathematics and physics. I had read that mathematicians are productive only when young, and I was already seventeen years old, so evidently I should postpone the physics. My schoolmasters had told me that I stood a fighting chance to get into the best university in the country, namely Cambridge, but to do so would require winning an open scholarship, because, coming from a school without an academic reputation, there could be no other route. I acquired some past examination papers, and discovered to my horror that I couldn’t even understand the questions in mathematics, let alone answer them; so reluctantly I turned to Natural Sciences. I learned that one had to apply to a particular college, and that the colleges were in groups with different exams. The Queens’ group’s examinations appeared to be marginally less formidable than the others, so that determined my choice. But which college amongst them? My headmaster suggested that I visit Cambridge for a day to see which college I liked best. So I did. On alighting from my bus in the market square, I was confronted by the spectacular chapel of King’s College in full sunlight. King’s was not in my preferred examination group, so I walked through and approached Queens’ College from behind. Now it was raining, and I faced a dull redbrick building; the prospect of being there seemed gloomy. So I doubled back to St John’s College, geographically the next on my route through the Queens’ list. At the entrance I was confronted by a porter who asked my business. When I told him, he gave me the most friendly welcome, telling me what a wonderful college St John’s is, and insisting that I meet the Senior Tutor straight away. That was the last thing I wanted because I was extremely shy and quite unprepared for an interview. Fortunately, the Senior Tutor was out. Buoyed with confidence from that experience, I went on to Emmanuel College and approached a porter with my story; he was a big surly man, and, looking down at me said: “You’ll never get in here”. So my choice was made. It may seem an unconventional way of choosing a college, but looking back on it I realise that it was actually not a bad one: the demeanour of the porters is surely a good indicator of the ethos of the entire community, and at least at John’s – as it is colloquially called, distinguishing it from St John’s College in Oxford – so it turned out to be. Back home, I applied for entry, and, lo and behold, some months later I received an unconditional offer of a place; my school must have sent a very convincing reference. I recall reading my letter many times, incredulously searching for the caveat that must surely be hidden within, but there was none. What an enormous privilege!

Some time later I returned to Cambridge, this time to sit the scholarship examination. Being in Natural Sciences, in addition to the theoretical papers in physics, chemistry, mathematics and general knowledge (which included translating from both a modern language and from Latin), I had two practical examinations, one in physics, the other in chemistry. The physics was straightforward, as was, so I thought, the chemistry. During the latter I had to carry out titrations involving the products of a strange chemical reaction, the principles of which were explained in general terms, and from the outcome one had to infer the molecular weights of the ‘unknown’ reactants. Presuming that the results had to be integers, I quickly carried out the work sufficiently precisely to obtain the answer, and then I ‘improved’ my measurements for reporting, adding small random errors to the ‘exact’ values. Notwithstanding the principles that Joe Brearley had instilled, in my view my behaviour was hardly unfair: the objective of a normal investigative experiment is to determine some property of the object under investigation, but the objective of this experiment was to get a scholarship. The university acceded, awarding me a scholarship only, I suspect, because I had declared that I was interested principally in physics. My award letter said that I had done well in all subjects except chemistry! Soon after coming up as an undergraduate, I was attracted by an extracurricular lecture in chemistry involving what seemed to be related to what I had encountered in my exam: the then little-known Belousov–Zhabotinsky reaction. Afterwards, I spoke to the lecturer telling him about my examination. It turned out that it had been he who had prepared the titration, with reagents contaminated in a known way, so he knew how I had come by my results.

Joe Brearley was overjoyed when he learned that I was to go to John’s; unbeknown to me, he was a Johnian himself. By now we were friends; indeed, Joe later came to our wedding. Before I left school, Joe gave me serious advice about how I should benefit most from my undergraduate time in Cambridge: work hard, play hard, and under no circumstance develop a taste for good wine, for that would destroy my bank balance. During my first two years I took the second and third pieces of advice to heart, and the first two thereafter.

In the spring of 1959, before entering Cambridge, I spent a weekend playing rugby fives against our sister school, Oundle, together with a team from the Clove Club, our ‘Old Boys’. The experience opened my eyes to a stratum of English society that I had not encountered before. We were in the dressing room when a so-called elite rugby team, called Harlequins, arrived. They were not prepared to share the dressing room with riff-raff like us from the East End of London, and insisted that we leave. In the Clove Club team was Keith Pavitt, an engineering student at Trinity College, Cambridge, who completely outclassed them intellectually, resulting in their leaving in indignation for a ‘preferable’ location, which turned out to be a mere classroom, with no showering facilities! That was my first meeting with Keith; we discussed my anticipated entrance to Cambridge, having to read natural sciences rather than mathematics. ‘That’s not necessary’, replied Keith: all I had to do was to write to my tutor asking to change. My request was granted immediately: one of the benefits of Cambridge over most other English universities is its flexibility. Thus, my laboratory days were over, so I thought.

That year I was also fortunate enough to be awarded a W.H. Rhodes scholarship to tour Canada (actually only Quebec and Ontario) in the summer. We were 48 ‘disadvantaged’ inner-city recipients, from London, Bradford, Birmingham, Manchester and Glasgow. We sailed on the Cunard liner Carinthia to Quebec, and then on to Montreal. One of the scholars with whom I shared a cabin was a Fraser, from whom I learned what Scots wear under their kilts. One night, in the middle of the Atlantic, the captain ordered everyone aboard either to go to the top deck or retreat to their cabins, because he was going to turn out every light on the ship. I went on top, of course, and witnessed a most spectacular aurora, shimmering from the pole, overhead, and well to the south, occupying some 70 per cent of the sky. On reading this memoir Ed Cliver informed me that the aurora was almost certainly associated with a well-known magnetic storm in mid-July 1959 linked to an episode of strong solar activity that by chance marked the start of Jack Harvey’s career (Harvey, 2020). Many years later, when I was resident astronomer on a tour ship sailing the Caribbean to view Halley’s comet, I casually went to the bridge to ask the captain to turn out the lights, and was instantly rebuffed on the ground that someone might trip and sue him. Eventually, I managed to persuade him to extinguish the lights on just the top deck, but it wasn’t the same.

On the day the Carinthia entered the St Lawrence Seaway we were enveloped in a thick early-morning fog. As the Sun rose the fog cleared, almost instantaneously it seemed, leaving a crystal-clear view of the cliffs. The only cliffs I had ever seen before were at Dover, and with no other scale of reference I assumed that these were of similar height. Then I saw what appeared to be a miniature model village by the water, with small boats manned by dolls, until I realised that the dolls were moving: they were real people! The shock of having suddenly to readjust my seriously faulty distance scale remains with me to this day. Our time on land was wonderfully educational. We were official guests of the City of Ottawa, meeting the Minister of Finance who subjected us to an excruciating economic screed, and we proudly signed the red-leather-bound, gold-edged inventory of official visitors; we visited McGill University; we were entertained in private homes to learn of ordinary Canadian life; and we were taken to a rather sophisticated (urban) ‘barn’ dance; we enjoyed canoeing at Lake Wanapitei, and there experienced a totally different boisterous (country) barn dance, during which in my enthusiasm I inadvertently threw my partner over a table; we went down a nickel mine in Sudbury, and I created great hilarity amongst the miners as being the first person they had ever seen standing upright at the rock-face; little did I know at the time that a mine in Sudbury was to become scientifically famous.

## Undergraduate Days

Mathematics in Cambridge was, and in some measure still is, different from other subjects. For example, it is the senior subject, that is, the oldest subject, aside of course from divinity and Latin that originally everyone studied. Abilities of incoming students were so disparate that scholars, presumed to be the most able of the students, skipped the first year of study. I was a scholar, but not in mathematics. So should I follow the first year’s courses? I remember timidly meeting my Director of Studies, Frank Smithies: a man of few words. He explained briefly the decision that he was to make. “How much linear algebra do you know?” he asked. “What’s linear algebra?” I replied. Long silence. “How much analysis do you know?” he continued. “What’s analysis?” ... . “You’d better do the first-year courses.” How right he was! And even then I struggled. It wasn’t until the end of the first term, flailing with analysis, before I sought help from another student, John McCutcheon, who explained to me the purpose of epsilon. Then all became clear. John and I have been good friends ever since. We helped each other a lot as undergraduates, he explaining the purer forms of mathematics to me, I explaining physics to him. The end-of-year (Tripos) examination was a horrendous experience: one is seated amongst one’s competitors, most of whom, I believed, were no doubt faring much better than I. Subsequent publication of the results confirmed my belief.

In my summer vacations I worked to supplement my scholarship income. First, in one of the two chewing-gum factories in England, initially on the gum-processing conveyor belt separating gum where it had been inadequately cut by blades that had been deliberately fractured by operators who had thrown, and still were throwing, spanners into the works in order to relieve boredom; then I was promoted to temporary foreman in the packing department, which was much more interesting, partly because of the wide variety of packaging for the many different brands. I learned that chewing gum could contain not only bits of broken blade, but also wasps, which were targets of football-sized pieces of gum surreptitiously used as projectiles before being processed. Needless to say, I never chew gum. Then, there was the parcel department at the Post Office during the rush before Christmas, from which I was fired because I worked too hard, thinking that I was relieving my workmates; according to my supervisor I was grossly mistaken, because had I continued the management would have expected similar productivity of the permanent staff. In fact, earlier I had been taught how to appear to work without actually doing so, which I found to be more tiring. So, when in the following summer I became a porter driving a delivery van through the grounds of a country hospital, I rearranged the duties so that I could do a whole day’s work in the morning, aside from delivering evening meals, and spend the afternoons secluded amongst the trees reading books. My best job was selling ice cream from a van, because not only was I my own boss but I could also use the van for my own purposes when not working. These vacation experiences taught me a very important lesson: I was certainly not going to spend my life in such routine employment.

One of the advantages of collegiate universities such as Cambridge and Oxford is that one is thrown together with interesting people in other subjects. Within days of my arriving at John’s I was invited by the Fellows to a formal dinner with the other scholars. It was in the Senior Combination Room, a long narrow candle-lit room with an ornate moulded ceiling, not built to accommodate the many people that were now present four-and-a-half centuries later. For this occasion it was furnished with two long tables, so cramped that the diners on the outer sides, by the walls, had first to establish where they were to be sitting and then file in in order. I was on an inner side, and opposite me at first was a vacant place. Later, as we were being served the soup, the tablecloth in front of me rose, and out popped a white-haired 68-year-old head, smartly dressed with black tie, as were the rest of us, but with tousled hair because he had just crawled under the table fifteen metres or so between the legs of the other diners. He smiled and said: “Hello! My name is Harold; these occasions are supposed to be formal, but clearly that’s not so!”. Later I learned that he was the Emeritus Plumian Professor of Astronomy and Experimental Philosophy, a very interesting man whom I was to encounter again. Much later, as President of the college mathematical society, I was dining next to Fred Hoyle, my hero from school days and the current holder of the Plumian chair, and our guest and post-prandial speaker, who told me how he thought Big Bangs might be reconciled with an asymptotic steady state of the Universe; and even later I found myself dining next to Francis Crick, who remarked that if one attaches together a string of similar long molecules all of which have connectors at each end that are inclined from the long axis of the molecule and do not lie in the same plane, then the resulting structure is necessarily a helix; so the structure of DNA is obvious.

In 1961 John’s celebrated its 450th anniversary, an important occasion because its significant anniversaries, we were told, occur only once every 150 years. There was dining and wining for all; we ate cygnet, because John’s is one of the few English establishments permitted to kill swans: in England all swans belong to the Crown (although these cygnets had actually been imported from Australia). The party ended with an impressive firework display as had never before been encountered in Cambridge, even on Guy Fawkes night. It caused great consternation amongst the city residents who were in trepidation of an air raid. Nowadays fireworks are commonplace, especially at May Balls (i.e. end-of-year balls that are held in most colleges after exams in May Week, May Week being the first two weeks in June), and residents now take them in their stride. How very different all this was from my earlier life, and what an enormous privilege to experience it!

John’s has grown enormously since it was founded, so much so that more tables now fill the dining hall, one down the middle and now two others, each on either side against a wall; and three sittings are required to feed everyone. On ordinary days, students cannot be expected to file in in orderly fashion against the walls as occurs in the Fellows’ Combination Room. So instead it has become the custom simply to walk across the stout oak tables, trying to avoid the other diners and their food. I write this because John Faulkner has also written on this subject (Faulkner, 2005), accusing me of treading in his soup. I point out here that John is a superb raconteur, who never lets the truth stand in the way of a good story. John has also published what he named “Gough’s Theorem” (Faulkner and Swenson, 1988), but I should not dispute that.

At the end of my first year at university the most wonderful thing happened: I was suddenly smitten by a friend of a friend, Rosanne, who eventually became my beloved wife. I spent as much of my second year as I could in her presence, which sometimes involved cycling to London where she trained as a nurse; I couldn’t afford the bus or railway fare. Consequently, at the end of the academical year I again performed badly in the Tripos examinations, but it was worth it. I had to redeem myself academically in my third year, however, for now I realised that academical research was a possible career, and I wanted to experience it. I recall going to the Senate House at the end of the academical year to hear the results of Part II of the Mathematical Tripos read from the balcony by the senior examiner, always at nine o’clock on the third Thursday of June: Mathematics is the only subject so privileged. I was relieved to learn that I was now a Wrangler.^4^ After the reading is over, the examiner throws from the balcony a couple of hundred sheets of printed results to rain onto the crowd below. I was lucky enough to catch one. I wondered whether one day it could be me standing on that balcony. Years later, on more than one occasion, it was.

**Figure 2:**
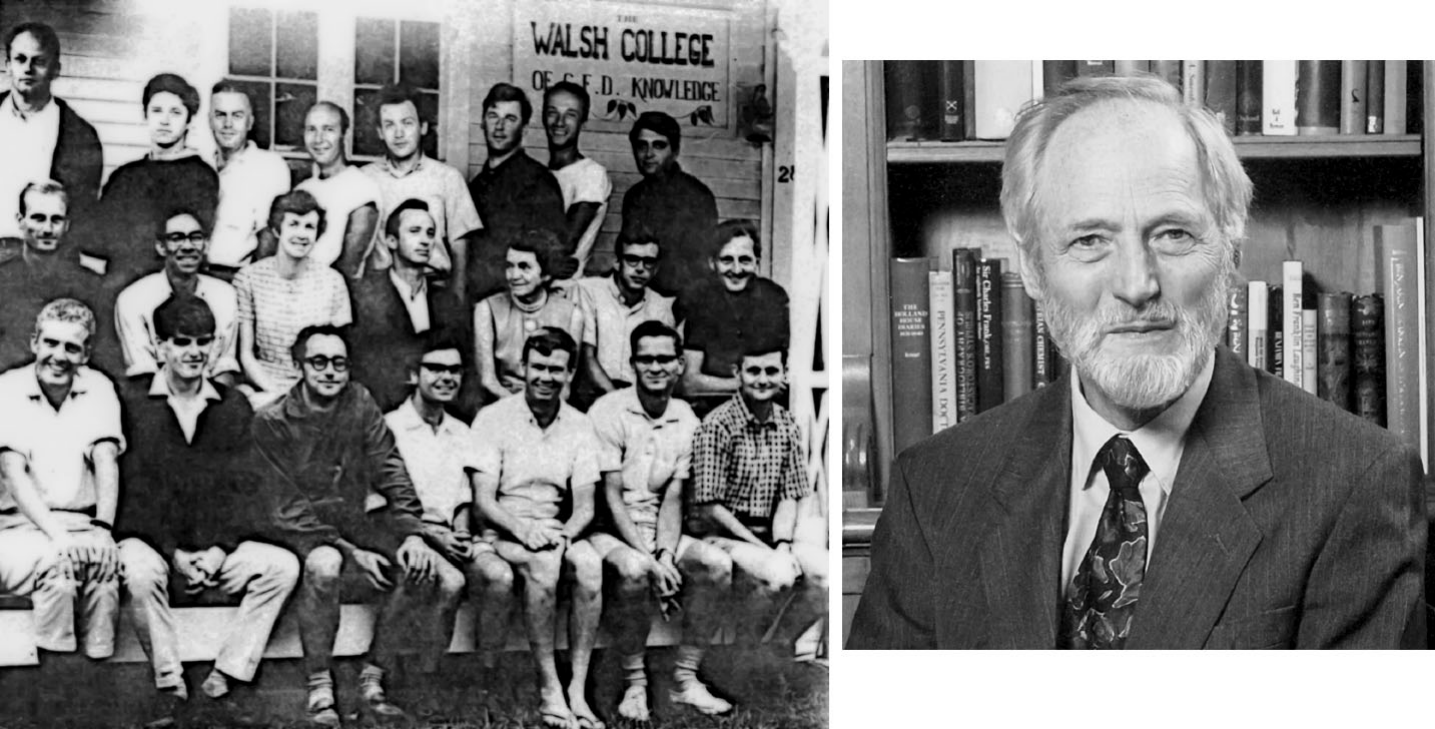
Left: Participants in the GFD programme in 1965. Back row: Tony Maxworthy, Frederic Bisshop, Stewart Turner, Joe Keller, Ed Spiegel, Pierre Welander, Andy Ingersoll and Allan Robinson; middle row: Matthias Tomczak, George Philander, Kathleen Trustrum, Willem Malkus, Mary Thayer, Joe Pedlosky and Francis Bretherton; front row: Hans True, Steve Crow, John Robinson, myself, John Booker, Bill Bowman and Brian Trustrum. Lou Howard, Stan Jacobs, Bob Kraichnan, Owen Phillips, Melvin Stern and George Veronis are missing. Right: My research supervisor Roger Tayler (with permission of the Royal Society).

After graduating I went on to study for Part III of the Mathematical Tripos, a course designed as a preparation for research. My long-term intention was to treat chemistry as a branch of physics, and so deepen our understanding of the subject. So I started taking courses that I thought were the most relevant to that endeavour. However, I found them tedious. So, half-way through the year I gave up the idea. I wanted a career in research, but of course I wanted it to be enjoyable.^5^ So I looked around the applied mathematics department, and observed that the most cheerful faculty were the astrophysicists: Roger Tayler, Dennis Sciama, Leon Mestel, Donald Lynden-Bell and Fred Hoyle. So in the second half of the year I pursued courses in astrophysics. It was a heavier-than-normal load because I had abandoned the first term’s studies, and therefore I had to supplement astrophysics with related courses to make up the deficiency. For the second time I was indebted to the flexibility of my university to permit such apparent waywardness. Perhaps as a result of offering only half-a-year’s work for examination, I didn’t get the distinction I had hoped for. But I was fortunate to have performed well enough to be accepted into the Department of Applied Mathematics and Theoretical Physics (DAMTP) as a research student, where I studied aspects of stellar convection under the guidance of Roger Tayler (Figure 2).

## Entering Research

As a research student I lodged with two other students, Jim Caswell and Mike Newton, on the top floor of a splendid Victorian house owned by Francis and Odile Crick. After our first year we tried to renew the lease, but were refused. That surprised us because we thought that we had been good tenants. Yes, we had, agreed Odile, but she and Francis wouldn’t renew because they planned in due course to demolish the house and replace it with a block of flats (apartments), no doubt with the help of the Nobel Prize; however, if we wished we were welcome to stay on without a lease until that happened. Thus, we remained until late 1964; Rosanne and I were to be married in January 1965, and I moved in briefly with some other friends, who also lived in a flat on the top floor of a large house. They were an enthusiastic, energetic crowd, all but one of whom in the fullness of time were simultaneously to become heads of academic institutions: Timmy O’Riordan, Head of the Department of Environmental Sciences at the University of East Anglia; Bob Stobie and Russell Cannon, Directors of the South African Astronomical Observatory and the Anglo-Australian Telescope, respectively; Tim Pedley and Malcolm Longair, Heads of DAMTP and the Cavendish Laboratory in Cambridge, all at the same time as I was Director of the Institute of Astronomy (Figure 5). The canny member of our crowd was Mike McIntyre, also in DAMPT, who succeeded in avoiding major administrative positions throughout his career.

My engagement to be married to Rosanne had been announced in The (London) Times the previous year. George Batchelor, the head of DAMTP, saw it, and sought me out at coffee time to tell me how pleased he was. From our few earlier encounters, I had learned how distant, yet open, he seemed to be to those whom, like me, he hardly knew. So I asked him why he was so pleased. “It’s quite simple”, he replied, “It means that you will get your PhD on time”. He was right. Of the dozen or so of my exact UK contemporaries (on three-year studentships), just three of us graduated within three years, and we were the only ones who married during that time.

After we were married, Rosanne and I rented a small flat above a pawnbroker with a bedroom window just 12 metres from a church bell, and at the same height. We never overslept on Sundays.

The first task that Roger Tayler suggested was to read a recent paper on stellar evolution by one of the authorities in the subject. I misunderstood what ‘read’ meant, and I presumed that I was to check the derivation of a new equation of state that was presented. I found an error. I thought naively that Roger would take the matter in hand. But no. He said that I, a green research student, should write to the great man and tell him. I checked my calculation several times before timidly drafting a tactful letter, in trepidation of the outcome. I was relieved when the response turned out to be quite friendly: it thanked me for my care, but continued with the assurance that the error did not ‘invalidate’ his evolution calculations because the new equation of state had not actually been used. That opened my eyes to the outside world of astrophysical research.

Roger then suggested I investigate the properties of convection in a radially pulsating star. I started by considering a series of convective-stability problems for an oscillating atmosphere under different idealised conditions. There were many interesting subtleties not far from criticality, but none applied at the high Rayleigh numbers pertinent to stars. I could not see how to formulate a more relevant problem that I could trust. So I shelved the matter.

Roger told me that he had become interested in convective instability in a star that is pervaded by a magnetic field, and invited me to join him in an investigation. We would seek a necessary condition for convection in a compressible atmosphere under constant gravity. Roger suggested that I approach the problem with a variational principle, and that he would work on it too. So busy was he with teaching in term-time that we hardly spoke for weeks, except for brief encounters at coffee during which I tried to keep Roger abreast of my glacial progress, and he would give me advice on how to continue. We had arranged to have a long meeting as soon as term was over, and I worked hard in order to have something positive to say when the time came. I succeeded in deriving a variational principle, and proudly showed it to Roger. He in turn showed me what he had done: he already had the makings of a preliminary criterion, but I was astonished to find that his starting premise was the variational principle that I had so laboriously derived. How could that be? It had already been published, in much greater generality, by Bernstein et al. (1958). Why hadn’t Roger told me? My term’s work had surely been a waste of time. “No it hadn’t”, insisted Roger, “By having derived it yourself you now appreciate it much more than you would have had I merely told you about it”. Once I got used to the idea, I realised what a gift he had given me. In the fullness of time I made similar gifts to my own students, but I doubt they all recognised them as gifts!

Roger and I eventually produced a series of necessary criteria for stability, although in retrospect I’m sure that my contributions could have been obtained more quickly by Roger on his own. Broadly speaking, convection can be stabilised if the ratio of the magnetic pressure to the total pressure everywhere exceeds $1-\gamma/\Gamma$, as one might naively expect from a back-of-the-envelope estimate. Here, $\gamma$ is the first adiabatic exponent and $\Gamma= {\mathrm{d ln}}p / {\mathrm{d ln}} \rho$ measures the background stratification. We published the work (Gough and Tayler, 1966), and I went on to study the necessary and sufficient conditions for the adiabatic stability of a plane-parallel polytropic atmosphere in the presence of a magnetic field. I was starting to write it up when Roger brought to my attention a draft paper from Syrovatskii (subsequently published by Syrovatskii and Zhugzhda, 1968) presenting the same results. Being beaten to press was a matter that students in DAMTP feared, yet oddly I was not terribly upset, because the published paper was so much better than what I believed I could have written. What is really upsetting, as I learnt later, is being beaten to press by a bad paper; I refrain from relating my experiences of that.

## Woods Hole

Early in 1964 I was advised to apply for a Fellowship at the Program of Geophysical Fluid Dynamics (GFD) at the Woods Hole Oceanographic Institution (WHOI), because then I would meet the world’s expert on astrophysical convection: Ed Spiegel. I didn’t know the deadline, and inadvertently I missed it. So instead I spent the summer at Culham Laboratory, the UK centre of nuclear fusion research where much plasma physics was being developed. There I met Nigel Weiss (Figure 9), who became a lifelong friend. The following year, at about the time of the GFD deadline, I received a letter from the Director, Willem Malkus, asking me why I had not re-applied. I wrote back to say that I couldn’t afford it because I was now newly married and would not leave my wife behind. Willem replied with an offer to accommodate the two of us in an old carriage house on site, and that he believed the fellowship stipend, which he could supplement with a travel grant from WHOI, would then be adequate. So I applied, and the Carriage House turned out to be delightful. The ten-week programme involved carrying out original research, writing it up, and in the last week lecturing on it to all the participants. I was fortunate to have Ed as my supervisor, who exposed me to a completely new way of thinking, quite foreign to my experiences in Cambridge. After I explained my desire to progress with convection in the astrophysical context, Ed suggested that I try to generalise to compressible flow a heat-flux-maximisation procedure that had been developed by Willem Malkus (1954) and Lou Howard (1963) for Boussinesq liquids. It turned out that in the governing equations there was a term that severely impeded my progress, so after a couple of weeks of struggling Ed suggested that I simply ignore it; that was something I would never have contemplated on my own because there was no evidence that it was negligible. Admittedly, I had been taught as an undergraduate by Leon Mestel who, quoting from The Bible, frequently told me to be “bloody, bold and resolute” and ignore small terms in equations describing complicated situations (although not when there is no justification for doing so). Nevertheless, I went off and took Ed’s advice, and after two or three weeks I returned with a very long face. Ed asked after my wellbeing. I told him that I had solved the reduced problem to obtain an upper bound, but that I had then used the solution to evaluate the omitted term and had found it not to be negligible. “That’s fantastic!”, cried Ed with great delight. I was totally bewildered, for clearly my result was useless. “You have just obtained the first result in the subject”, continued Ed, “It doesn’t matter whether it’s right or wrong; it’s worthy of celebration.” What fantastic optimism! Needless to say, I could not offer the calculation for my project report, so, building on the experience with my first project as a research student in Cambridge, I hurriedly put together, with Ed’s advice, the germ of a generalisation of mixing-length theory that could be applied to radially pulsating stars, and thereby conceal my flux-maximisation failure. My presentation at the end of the programme was the first lecture I had ever given, and I had made copious notes in fear of making a mistake. Ed’s advice afterwards was that in future I jettison my notes, a frightening prospect in the days before computer-generated presentations. He argued that if I could not remember the details of my presentation, it would bound to be too complicated for the audience to follow. So in future I did as he bid, and my lecturing improved.

Local, officially only non-academic, organisation of the GFD session was carried out by Mary Thayer, a wonderful otherwise retired widow of a WHOI oceanographer. She made sure that everything went to plan. She also typed and edited the booklet of lectures that is published each year, and always succeeded in getting the Fellows’ manuscripts on time: she simply threatened to write herself any manuscript that wasn’t received in a timely fashion. She was formidable enough to be quite plausible, and it worked. Mary also made mid-morning coffee. She started by boiling water just outside the open lecture room, in a large somewhat battered aluminium pot, so old that its bottom was rounded. It stood on a flat hotplate, on which the pot would continually rock from side to side, each side receiving a thermal impulse and emitting an acoustic pulse whenever it touched the plate, thereby (most times) driving an oscillation of the pot and a resonating surface gravity oscillation of its contents in a manner mechanically similar to pushing a child on a swing, and thermodynamically like the maintenance of intrinsic stellar pulsations, the impulsive thermal contacts acting in the manner of Eddington’s valve. As the water approached boiling point the magnitude of the thermal impulses decreased and the turbulent viscosity increased, and the oscillation ceased. The acoustic arrest was apparent inside the lecture room, causing, perhaps subliminally, unrest enough to ensure a timely termination to whatever lecture was in progress. Surprisingly, I had difficulty in arousing interest in the phenomenon amongst the other, typically oceanographic, Fellows (Figure 2). Yet how appropriate for me, who was investigating such a process in stars.

After Woods Hole I returned to my final year at Cambridge. I worked further on the interaction between convection and pulsation, obtaining evidence that convective stabilisation principally via the modulation of the mean heat flux might be responsible for the red edge of the ‘classical’ stellar instability strip in the HR diagram, and that destabilisation by the Reynolds stress (turbulent pressure) could dominate at lower effective temperatures and be responsible for the oscillations of the long-period variables. I was excited by these early results, and by now I was sure that I wanted an academic career. My hope after obtaining my PhD was to work with Ed Spiegel. However, Ed was about to spend a sabbatical year in Cambridge, which was the one place I could not be. At Ed’s advice, I applied to the Joint Institute for Laboratory Astrophysics, now JILA, for a fellowship to work with John Cox. Fortunately, I was successful.

Prior to Woods Hole, on Easter morning 1965, I was in the DAMTP library reading three papers by Lord Rayleigh on instabilities in rotating fluids. In the first (Rayleigh, 1916) is derived what is now called the Rayleigh condition: a uniform incompressible inviscid flow, circularly swirling, is stable to axisymmetric perturbations if the magnitude of vorticity increases outwards everywhere (I paraphrase); the other papers (Rayleigh, 1880, 1895), which are devoted predominantly to rectilinear shear flow, show that a necessary condition for instability to two-dimensional perturbations is that the vorticity has a turning point somewhere.^6^ What interested me was that, although incomplete, the two criteria both involved (potential) vorticity alone. As I was pondering the matter, in walked Donald Lynden-Bell. He was at a loose end because his wife Ruth was in hospital about to give birth to their first child, and he had come into the department to shelter from the snow – not typical weather in Cambridge, especially at Easter. Rosanne was in the same hospital, nursing. It turned out that Donald had been thinking along similar lines, but more thoroughly (and more deeply) than I, and was in the process of generalising the results to inhomogeneous fluids that were neither in pure rotation nor purely rectilinear. We talked for the rest of the day, and came to the conclusion that any turbulence resulting from such instabilities, especially if the dominant energy-containing eddies are almost two-dimensional, produces Reynolds stresses that would, in the first instance, tend not to smooth out large-scale velocity, as was commonly believed, but more likely large-scale vorticity instead. We conjectured that turbulence caused by, for example, convection, would behave similarly. We subsequently learned that G.I. Taylor (1915, 1932) had had similar ideas many decades before, and had developed his mixing-length ideas based on vorticity transport. Moreover, Francis Bretherton and Stewart Turner, in DAMTP, brought to our attention the work of Richard Scorer (1965), an eminent meteorologist at Imperial College, who in an unpublished report also concluded that turbulence tends to homogenise vorticity, although his view of the large-scale consequences were quite different from ours: Scorer argued that redistributing angular velocity such as to (almost) homogenise vorticity leads to an essentially singular vortex that, he argued, explained the formation of tornados and hurricanes; Donald and I had envisaged the system to evolve further, and that eventually there would be no singularity. Angular momentum had therefore to be expelled.

We needed experimental verification. My undergraduate belief that my days performing laboratory experiments had come to an end was about to be shattered. At the suggestion of Thomas Brooke Benjamin, we floated a beaker of water in another, larger, beaker on the departmental turntable in the basement, and generated turbulence in the inner beaker with Alka-Seltzer, an analgesic that effervesces in water. If angular velocity were expelled from the water in the inner beaker, because that beaker and its contents had to conserve angular momentum, at least initially, the beaker would speed up. Each morning, on the way to the department, I stopped at the local pharmacy to buy a tube of Alka-Seltzer tablets. The pharmacist was bemused, because I didn’t look sick; however, after about a week, I regularly arrived to find a tube on the counter awaiting me. As Donald and I were preparing our apparatus, George Batchelor, the most senior fluid dynamicist and head of department, told us that the experiment was not worth performing, for he could prove by logic alone that we would get a null result. Nevertheless, we did perform the experiment, and the beaker did speed up. That caused every fluid dynamicist in the department, including George, together with my astrophysics friends Brandon Carter and Stephen Hawking, to come downstairs to see for themselves and offer alternative explanations, which Donald and I set forth to test. In the end we wrote a paper, probably the most controversial of my career, and sent it to the *Astrophysical Journal*, which Chandrasekhar rejected instantly as being irrelevant to astrophysics. So we sent it to the Journal of Fluid Mechanics, which George Batchelor edited. It went to four referees: two said that it was one of the most original papers they had ever read and that it should be published without modification, the other two that it was the most ridiculous nonsense they had ever seen and under no circumstance should it see the light of day in any journal. George had to make the casting decision, and because his prediction had been shown wrong, he honourably published it (Gough and Lynden-Bell, 1968). Some time later Strittmatter, Illingworth, and Freeman (1970) demonstrated that the cool inner beaker caused thermal currents in the outer beaker to spin it up, which vitiated our argument (but not necessarily our conclusion; they had not shown that the turbulence had no effect at all). I should add, to show off my experimental prowess, that I had observed that the Alka-Seltzer reaction is endothermic, and had measured the heat of reaction to be −50 calories per tablet, but Donald and I had misjudged the extent to which that biased our results.

Later, I sought to find a quasi-steady state in a large cylindrical rotating tank of stratified saline, heated from below, undergoing layered turbulent convection, the purpose of the layers being to insulate dynamically the main body of the fluid from the base of the container. I made neutrally buoyant direction indicators that floated in the upper layers, and I recorded their directions each rotation. I had to stand in an inertial frame (at that time the department had no television camera that could watch from the rotating frame). It was extremely tedious. However, I was rewarded after four continuous days by the realisation that the average angular velocity of the fluid appeared to be significantly less than that of the turntable; some vorticity, and with it angular momentum, must surely have been expelled. But on the fifth and sixth days the reverse happened, and although my statistics were not yet secure I was moved to check the speed of the turntable. It turned out to vary some hundred-fold more than its specification. The turntable was returned to the manufacturer, who reported that the thrust bearing had been installed upside-down! Weeks later the turntable reappeared in DAMTP with an assurance that it now satisfied specifications. However, I was too disheartened to face several potentially fruitless weeks giddily watching the orientations of half-a-dozen floats. Perhaps my days as an experimental physicist really were over. However, that was not to be so.^7^

My PhD dissertation contained three chapters: on the effect of an externally imposed magnetic field on convective instability, on the interaction between convection and pulsation, and on the interaction between convection and rotation. I have maintained an interest in all three subjects ever since. The thesis was written in just three weeks, against the deadline of the birth of Rosanne’s and my first child. In the event, the birth was late, so I then had time to relax, or bite my thumbs. After a week I drove Rosanne fast down a long uneven country road, ensuring that the wheels of the car rode over the most severe undulations. The next day our first daughter, Karen, now Kim, was born, on the day that England won the FIFA World Cup (an achievement yet to be repeated).

All that was now required for the PhD degree was to survive my oral examination. There were two examiners, one internal to the university, the other, Paul Roberts, external. The examination was conducted in the morning. After settling into some details about my computations of the convective stability of a compressible atmosphere in the presence of a magnetic field, Paul drew my attention to my graph of a measure of marginal stability against a dimensionless measure of the depth of the fluid layer, the values plotted having been computed as eigenvalues of a two-point boundary-value problem. I had not noticed that there were two depths for which, from the information provided in my dissertation, the values could be evaluated in one’s head (or at least on the back of a large envelope), and my plot did not go through them. Paul said that if I could locate the error by the end of the day, and if correcting it would make no qualitative difference to my conclusions, then I could make a minor correction and he would be satisfied; otherwise I would have to resubmit the thesis. I went away, quaking with fear. Then, to my relief, I found that the mistake was indeed minor. The graph had been hand-drawn – computer graphics were not available in those days – and my original draft would not fit on the page, so I had had to rescale it. It turned out that all that was wrong was simply that the ordinate scale was mislabelled by a constant factor. The correction was therefore trivial to undertake, and made no material difference to the conclusions in the thesis. A couple of years later I encountered Paul at a conference in Newcastle held in honour of Keith Runcorn, and I asked him how he had discovered my erroneous values apparently so readily. Surely he had not undertaken to check everything in the thesis. No, he replied; he had merely followed his normal practice: to open the thesis at a random page and locate the mistake. It was four years after my own examination that I was an examiner myself. I decided to emulate Paul. Lo and behold, I found a mistake on the first random page I selected. That too turned out to be easy to correct, although it did require cutting out two pages and inserting new ones – dissertations were properly bound before submission in those days. The error I found in the second thesis I examined required replacing a dozen pages. Subsequent candidates have been more careful (or perhaps I have been less assiduous), and I no longer find errors on the first random page I encounter.

A few days before leaving Cambridge for JILA, Rosanne and I were invited by my tutor, Alan Welford, and his wife Ruth, to their home for dinner. I had been fortunate enough as a scholar of John’s to have experienced some interesting wine, but what Alan and Ruth served that evening was more than just interesting, it was stunning. “It’s just something I picked up from the college cellars”, said Alan modestly in response to my praise; it opened my eyes, or perhaps I should say my palate, to a new world. For the first time in my life I took the trouble to remember a wine’s name: Chateau Lafite Rothschild. Henceforth, Joe Brearley’s third piece of advice went out of the window.

## JILA

I really enjoyed the environment at JILA, so different from Cambridge, in an institution that, like Cambridge, has a number of brilliant physicists. I made a great many new friends. John Cox was a quiet man, with whom one had to commune a long time before one could appreciate the depth of his understanding. At the opposite end of the spectrum was the very vocal Dick Thomas, one of the founders of JILA, who always spoke his mind, upsetting the timid amongst those with whom he disagreed. I enjoyed the scientific bantering with Dick; it not only made me seek several different ways of explaining a point, but also, through his appreciation, made me more confident in myself.

My main activity during that year was to complete a formulation of my mixing-length approach to convection in a pulsating star, and to increase my knowledge of other branches of astrophysics. I introduced and ran an evening seminar series, such as I had encountered previously at Woods Hole, for discussions of nascent ideas. To keep matters informal and friendly I served beer and snacks from the outset, but was quickly informed that it was illegal because at the time the university campus was dry. So Dick offered his house in the mountains, which enhanced the proceedings with its stunning views. In winter, after snow, it was a long journey from the campus. On one occasion the speaker failed to arrive. The audience could not be sent home un-entertained, so in the absence of a volunteer I had to speak myself. I was completely unprepared, and had neither notes nor slides, so I spoke on vorticity expulsion by turbulence because there were no complicated equations to construct. It was only the second lecture of my life. Uriel Frisch was there, passing through Boulder from Nice, and was intrigued by the ideas; our interaction engendered many subsequent discussions during my sabbatical year in Nice. Amongst the topics were how turbulent vorticity expulsion could explain the observed sharp jump in stellar rotation velocities along the main sequence, and the ‘equatorial acceleration’ of the Sun’s convection zone (Gough, 1966).

Within a few weeks of arriving at JILA I received a letter offering me a professorship at the University of Alberta, coupled with being chairman of the Geophysics Division of the Physics Department. Obviously the letter had been misdirected, even though it was addressed explicitly to me, Douglas, and had been forwarded from my previous correct DAMTP address in Cambridge. I wrote back admitting that I was probably not the intended recipient. I was a little surprised never to receive a letter of thanks for obviating a potentially embarrassing situation; what would have happened, I sometimes wonder, had I accepted the offer? Later, I became aware of the existence of the geophysicist Dennis Ian Gough; our paths were later to cross on other interesting occasions.

Rosanne, Karen and I had a wonderful year. We stayed in a ‘Chautauqua’ cottage near the edge of town. JILA had organised it for us in advance, with some reluctance (we had wondered why). It turned out to be little more than a crude wooden shack, its only form of heating, in the main room only, being an almost uncontrollable gas-fired furnace under an almost red-hot grill in the floor that we had to rope off once our infant had learned to crawl. This taught us one of the important differences between two ostensibly similar languages from either side of the Atlantic Ocean: for us the word ‘cottage’ had conjured up visions of a small stone- or brick-built, possibly thatched, country abode.

We were amazed by the rapidity with which the weather in Boulder could change. We went to the theatre one evening early into our sojourn, dressed for summer, and were faced by nearly 50 cm of snow at the end of the performance. The locals cheered in anticipation of skiing. Our hearts dropped, because all our warm clothes were in a dock in Montreal awaiting the end of a stevedores’ strike. We enjoyed visiting many interesting places in the vicinity. One memorable trip was to the Santo Domingo Pueblo in New Mexico to witness the spring corn dance. We had been advised to leave our car well outside the Pueblo, and then to watch the dancers from a respectfully distant location. But that was not to be so. I was carrying Karen on my back in a ‘newly invented’ Gerry Kiddie Carrier; the Indians (locally known as Native Americans) had never before seen a paleface carrying a child in the Indian way, and we were all immediately accepted into the festivities. Only the males danced, in a long line from the most senior to children who were barely old enough to stand. We stayed, amongst the women, for most of the day, fascinated. Finally, we moved on to Mesa Verde. We had left Boulder in the snow, leaving the heating in our cottage at the lowest we dared (against a fear that the wind might otherwise blow out the pilot); we returned a few days later on a hot sunny day. The temperature in the cottage was unbearably high, and I had to leave the family outside while I opened all the doors and windows and scraped the candles from the dining table over which they had flowed.

We partook in several local activities: Rosanne and I joined a baroque ensemble and we both learned to play the recorder; Rosanne worked voluntarily at Wardenburg, the University of Colorado’s Health Center (her UK medical qualifications are not recognised for full nursing in the USA) where, amongst other duties, she cared for a student dying from a rare disease, the man whose later apparition at A Midsummer Night’s Dream I described earlier in Section 2; I joined the JILA skiing seminar, where I graduated from snow-ploughs to parallel turns, ‘taught’, as he put it, by Peter Wilson, visiting from Sydney, who simply took me to the top of a piste on which all routes down were too steep to do otherwise.

**Figure 3:**
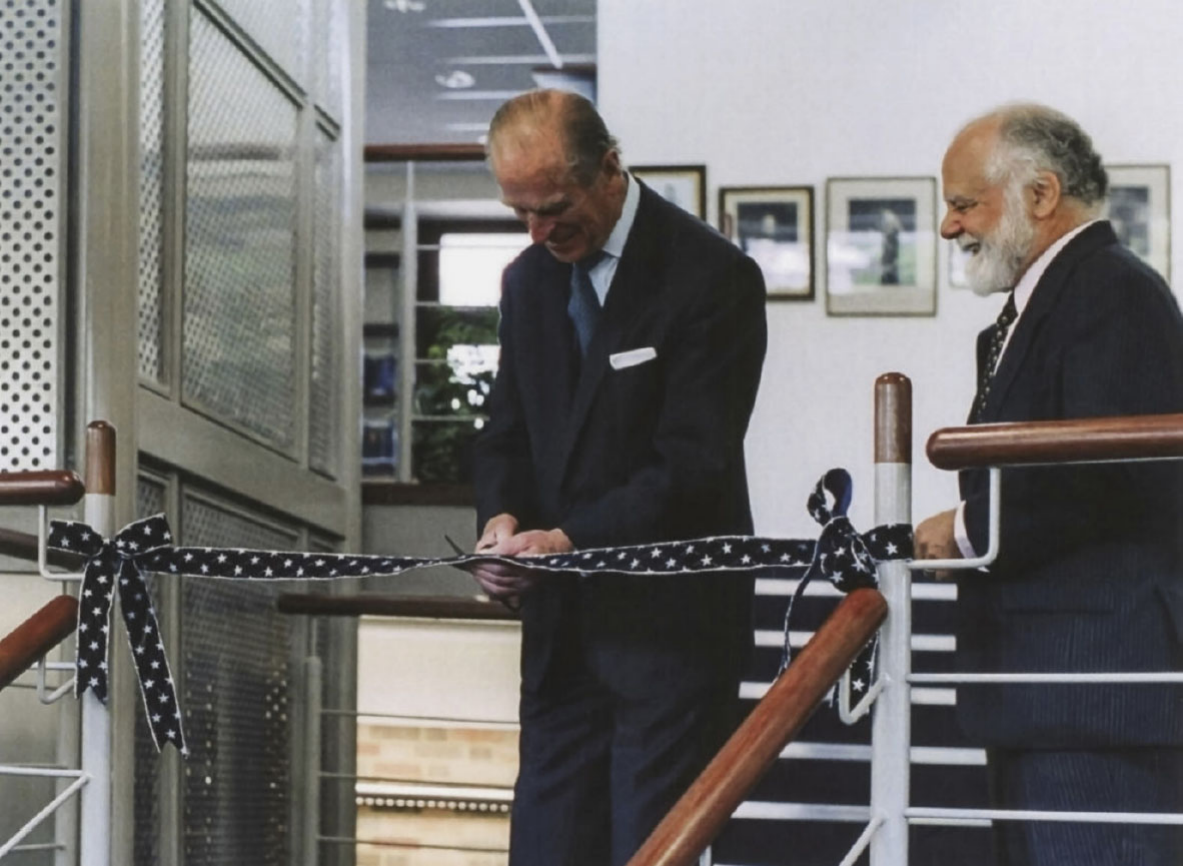
Opening the Corfield Wing of the Institute of Astronomy, Cambridge. Photo: Richard Sword.

When we arrived in Boulder, JILA was housed in two separate buildings. Mine was for theorists, a late-nineteenth-century stone structure with creaky wooden floors. I quickly learnt the different footfalls of my new colleagues, to the extent that I, and no doubt everyone else, was always aware of who else was in the building at any given time. It was a comfortable feeling. Half-way through my stay, JILA was united in a new building: a brick-and-stone-faced concrete tower with a central cavity housing two elevator shafts and a never-to-be-used experimental space that effectively isolates the offices from all but their immediate neighbours. It taught me that effective human communication is horizontal, a lesson from which I benefitted decades later when planning with open-minded architects the design of an extension to the Institute of Astronomy in Cambridge (Figure 3), made possible by a kind donation from my sometime student Nick Corfield.

## The Convective Collective

After JILA I went to New York, as a National Research Council (NRC) Senior Resident Research Associate at the Goddard Institute for Space Studies (GISS), to work with Ed Spiegel. Rosanne and Karen flew via England, so that they could visit family; both JILA and the NRC paid return fares from the UK for me and my immediate family. However, I drove across the country instead, in order to get our vehicle to New York. The journey gave me an appreciation of more than a thousand continuous miles of maize. It also left an indelible memory of a (not-to-be-my-only) near-death experience: I was driving on an open freeway out of a valley in Illinois when an articulated truck coming downhill on the other side lost a front tyre and headed out of control across the unguarded central reservation straight towards me. To try to stop would have been obvious suicide, so I tried to accelerate up the hill in our under-powered station wagon, heavily laden with all our belongings. The response was sluggish, and I braced myself for a mighty impact. The lorry turned onto its side, which fortunately slowed it somewhat, and it missed by a hairsbreadth. The driver was unhurt; both of us were in shock. To calm myself I drove onto more minor roads through Indiana, adding a hundred miles or so to the journey, which later prompted an unpleasant objection from petty NRC bureaucrats who were to reimburse my travel costs. In New York we rented a tenth-floor apartment on Riverside Drive, which Rosanne’s mother had secured for us as the result of a casual conversation with a shop assistant in Cambridge who, it transpired, had a daughter about to return from Manhattan and needed a sublet. By chance, the apartment was just two blocks from GISS.

Being at GISS was another whole new experience. When computing I often worked late into the night, and would shop for groceries on Broadway at 3 am on the way home. In that way I got to know, casually, quite a few of those interesting New Yorkers who always shopped for food at that hour.

It turned out that Ed had invited another DAMTP research student, my exact contemporary Jüri Toomre, to join us. We cemented our acquaintance at GISS, and have been good friends ever since. Ed had encountered a mathematical expansion procedure initiated by Paul Roberts for studying Boussinesq convection, and suggested that the three of us develop it. The idea was to expand the horizontal dependence of the velocity and fluctuating temperature in eigenfunctions of the linearised problem and then project the governing equations onto those eigenfunctions, leaving a set of coupled differential equations for the amplitudes of the resulting ‘modes’, as we called them, with respect to the vertical coordinate and time. The intention was to be able to concentrate on the vertical variation of the thin horizontal boundary layers that were bound to be present at high Rayleigh number (a dimensionless measure of the thermal stratification), in the hope that any associated poorly resolved horizontal variation would be of lesser importance. Jüri, whose expertise was in numerical modelling, studied the temporal evolution of a severely truncated expansion (with 1, 2 or 3 modes), while Ed and I worked on an asymptotic analysis of the steady state of a single mode. Ed and I spent many a long evening discussing the details of the analysis by telephone, each from his own home, feasible because local calls are free in the US. The principal goal was the relation between the Nusselt number, $N$ (a dimensionless measure of the total heat flux), and the Rayleigh number, $R$, for given Prandtl number (the ratio of viscous to thermal diffusion coefficients). We found the going very tough, and for a long while we were unsure whether we had established the relation correctly. The leading order was the product of a power of $R$ and a power of ${\mathrm{ln}}R$, and we dearly wanted numerical confirmation. Evidently, getting the power of ${\mathrm{ln}}R$ would require very high values of $R$, which implied extremely thin boundary layers. Jüri’s time-dependent code used a piecewise-constant mesh, suitable for ensuring precise adherence to certain conservation properties of the solution, but only away from any mesh discontinuity. Somehow that was deemed essential by numerical experts of the day. However, the discontinuities generated artificial disturbances that interfered with convergence, severely limiting the value of $R$ that could be attained. In contrast, I believed that a continuously varying mesh would not only be more stable, but also more informative: a check on the conservation laws *a posteriori* would provide a measure of accuracy. To spur us on Ed had offered two carrots for successful computations at large $R$: dinner with a good wine from his collection (I no longer remember what it was) for reaching $10^{15}$, and Chateau Lafite Rothschild 1959 for $10^{20}$. Because it was I who believed in the efficacy of continuous mesh variation, it was left to me to effect a demonstration. I used centred second-order-accuracy differences, and solved the two-point, non-linear, boundary-value problem (of eighth order for a single mode) by Newton–Raphson–Kantorovich iteration, using a procedure that I had evolved from one that Norm Baker and Rudi Kippenhahn had used to study stellar pulsation (Baker and Kippenhahn, 1965). When I considered the programme to be working correctly, I submitted a job for overnight running, starting with $R=10^{4}$, which I knew was easy, and then successively increasing $R$ by a factor $\sqrt{1}0$ in a FORTRAN do-loop, always using the previous solution, appropriately stretched and scaled, as a trial. With great optimism I set the loop to end at $R=10^{20}$. The next morning I collected the line-printer output: it was 10 cm or so thick, weighing several kilograms.^8^ I turned to the last page to see where the computation had crashed, and was amazed that it had reached the end of the do-loop. I was overjoyed. To celebrate the success, Ed and his wife Barbara had a dinner party for the team and our wives. The first wine was wonderful; then came disaster: someone knocked over my glass of Lafite Rothschild; Rosanne, who was newly pregnant with our second daughter, had already declined, and there was no wine left in the bottle. So I never even tasted the main prize. In recompense, just before I was to leave New York a year later to return to Cambridge, Ed very kindly gave me a bottle of 1959 Chateau Latour.

My procedure for determining the mesh on an independent variable $x$ was to imagine a transformed variable $\xi$ on which the mesh is uniform, choosing $\xi(x)$ such as to minimise the integral of the sum of the squares of the first or second derivatives with respect to $\xi$ of all the dependent variables and however many of their derivatives contributed directly to the (differencing error in the) differential system. I called it mesh stretching. Some years later we presented the procedure at a conference on numerical analysis organised by Bob Richtmyer (Gough, Spiegel, and Toomre, 1975a). Jüri had a brilliant presentational idea to attract the attention of the audience: I gave the lecture, walking amongst the audience, much of the time without looking back at the screen, and Jüri was hidden from view holding the controller of the slide projector – this was long before the days of computer-generated radio-controlled lecture presentations – he knew the talk intimately, and changed the slides as needed with no prompting from me. The audience was evidently amazed, having never seen the like before, and many remembered the presentation long afterwards. Whether they remembered the mathematical content is another matter.

**Figure 4:**
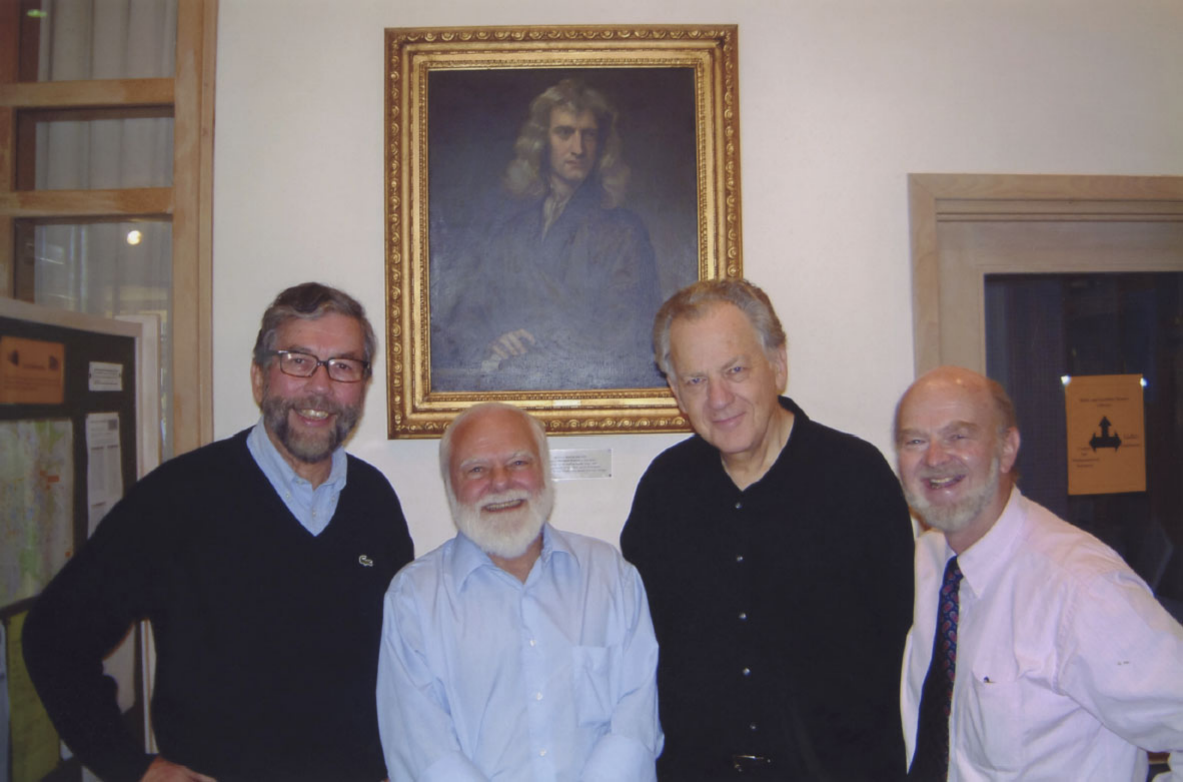
The Convective Collective at the Isaac Newton Institute, Cambridge, in 2004. left to right: Jean-Paul Zahn, myself, Ed Spiegel and Jüri Toomre. Photo: Sasha Brun.

Jüri’s computations revealed a quite complicated behaviour of our admittedly severely truncated expansion. It was evident that, interesting as it was, we were probably not faithfully representing the very-high-Rayleigh-number convection present in stars, which had been our goal. Moreover, the Boussinesq approximation had to be relaxed to admit compressibility; that led to my formulation of the anelastic approximation suitable for describing subsonic convection, more about which I report later. We had also formulated a representation of plane Poiseuille flow using the same expansion technique, and Jüri had carried out some computations; the work was completed with the help of Jean-Paul Zahn, who came to New York at about the time I left, after which communication was too slow for me to be able to make further significant contributions. Nevertheless, someone dubbed us ‘The Convective Collective’ (Figure 4). Ed, Jüri and Jean-Paul were joined by Jean Latour, and used the technique to study relatively shallow convection zones in A stars (e.g. Latour et al., 1976; Toomre et al., 1976), which was no doubt better than using the only other alternative of the time, namely mixing-length formalism. After that, the technique was almost completely abandoned; Jüri sensibly moved on to develop more realistic direct simulations of convection, and we others moved on to other things. The Collective eventually published our work in four papers in the Journal of Fluid Mechanics (Zahn et al., 1974; Gough, Spiegel, and Toomre, 1975b; Toomre, Gough, and Spiegel, 1977, 1982); it was to be thirteen years after my departure that the last of them appeared. Ed and Jüri believe that the inordinate delay was largely my fault, especially since the last of the studies to have been completed – that with Jean-Paul – was the first to be published. Perhaps they are right, although I can think of other possibilities that are consistent with the data.

**Figure 5:**
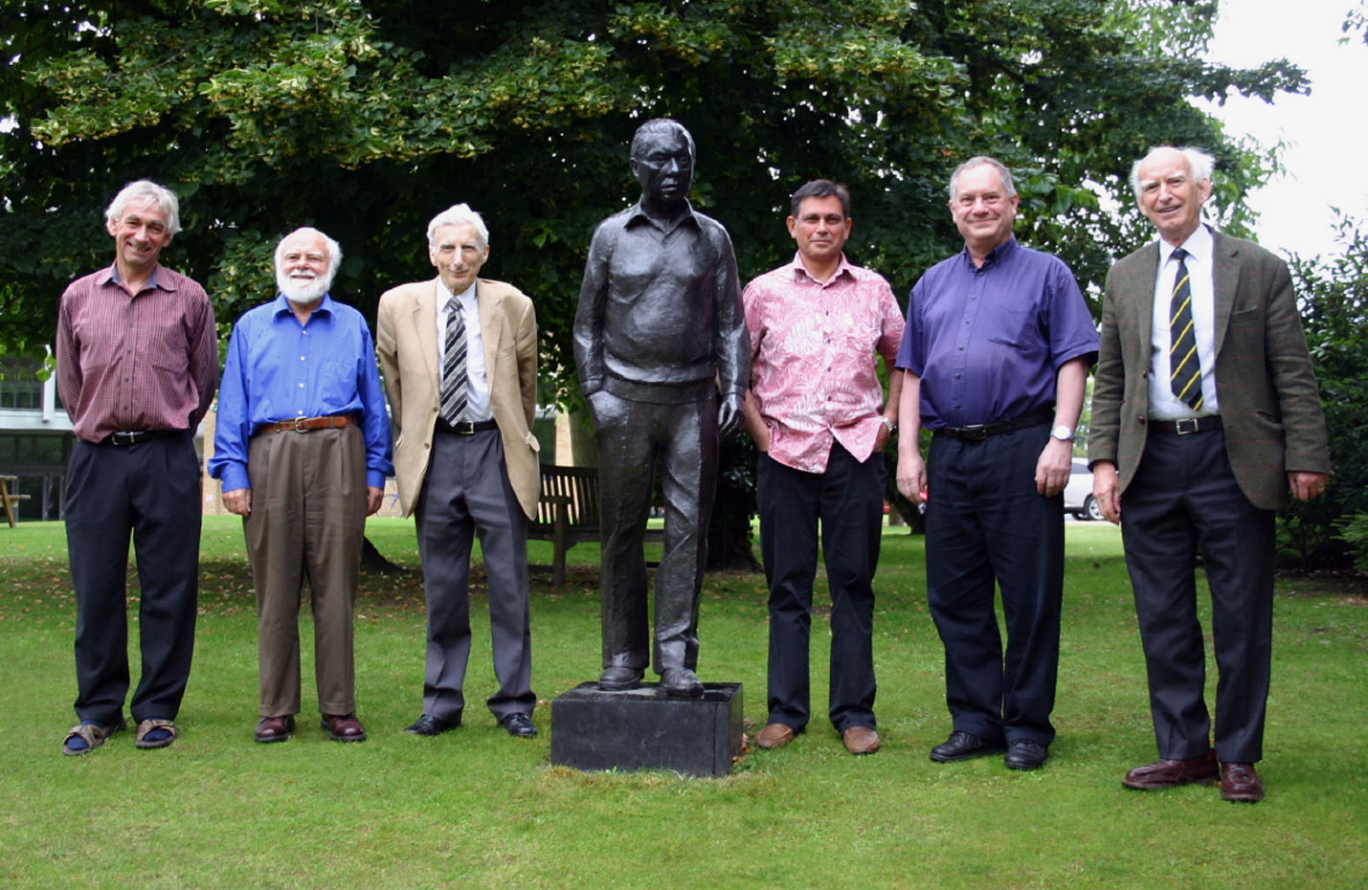
Past Directors of IoA. left to right: Richard Ellis, myself, Martin Rees, Fred Hoyle (Founder of IoTA), George Efstathiou, Rob Kennicutt, Donald Lynden-Bell. They have been succeeded by Paul Hewitt, Andy Fabian and now Richard McMahon. Photo: Amanda Smith.

My experience in New York was both exciting and stimulating. Ed tried to persuade me to remain, suggesting an Associate Professorship in the Astronomy Department at Columbia University. It was tempting, but Rosanne and I had vowed before we left England that any decision about the side of the Atlantic on which we might settle would be made in England. Ed saw this as the symptom of a disease that Cambridge inflicts on most of those who go there; being so inflicted, I needed to seek employment back home. In my second year in New York I was fortunate to be funded to attend a conference in Newcastle. By now our second daughter, Heidi, had been born, and I took the opportunity to take her with me to meet her grandparents. Born in New York, Heidi has dual citizenship, rendering crossing the Atlantic much easier than it is for the rest of the family. Her birth had been overdue, we believed. Recalling the delay of Karen’s birth, after three weeks delay Rosanne and I travelled up and down Manhattan on the IRT (subway) express, sitting immediately above a bogey. It worked a charm. Next morning, in the early hours, Rosanne had gone into labour. The taxi driver who took us to the hospital must previously have had a birth in his taxi, because he drove at breakneck speed paying no heed to traffic lights. On arrival I was presented with paperwork to complete, and as a result I nearly missed the birth. I finished just in time, and was delighted that our Heidi’s first action was to grab the oxygen mask that had been unnecessarily forced upon her, and tried to pull it away. On reflection, I wonder whether her birth was actually one week early.

After the Newcastle conference I paid a visit to the new Institute of Theoretical Astronomy (IoTA) that had been established by Fred Hoyle after I had left DAMTP. There I was reunited with some old friends from student days. Amongst them was Peter Eggleton, to whom I explained how to simplify my mesh-stretching technique to make it easy to use in his computations of stellar evolution. But the man whom I really wanted to meet was Fred Hoyle, to ask him for a job. Alas, he was in London at a meeting, as I learnt from his wife Barbara, who promised to relay my desire. A few days later, walking to the lecture room to deliver a seminar at Queen Mary College in London at the invitation of Ian Roxburgh, I was intercepted by Ian’s secretary who told me there was someone on the phone wishing to speak to me. There isn’t time, I said, I’m about to give a seminar. It’s Fred Hoyle, she replied. So I changed my mind. Fred started by telling me what he had understood from Barbara, then straight out suggested that I arrive on the first of October. I had made no formal application. It had taken Fred a few days to respond in order to take up references by telephone. The offer was initially for just one year, not the tenure that Ed had suggested for Columbia. However, at least my immediate future was now secure. Ed was bemused. Soon afterwards I was also offered an Associate Professorship by the University of Colorado, coupled with a JILA Fellowship, which I also declined for the same reason as the Columbia offer. However, it provided an opportunity for recommending Jüri for the post.

## Anelastic Equations, and Other Unobvious Obvious Results

In New York I thought about how to represent the equations of motion of a deep layer of compressible gas in a form amenable principally to the expansion procedure that Ed, Jüri and I were using to describe thermal convection in a Boussinesq liquid. Ed and George Veronis had written an extremely lucid paper deriving the Boussinesq approximation under conditions applicable to convection at low Mach number in thin layers of gas (Spiegel and Veronis, 1960); and Ogura and Phillips (1962) had presented an anelastic analysis of adiabatic motion that did not account for the thermal boundary layers in complete convection zones. So I set to amalgamating the two approaches, developing an expansion at low Mach number for an arbitrarily deep layer. I thought the outcome to be obvious; certainly not worth publishing. Ed was doubtful because in his Boussinesq analysis with George it had been possible to establish a background state independently of the final solution against which a thermodynamic linearisation was made, whereas I asserted that is not possible for deep fluid layers. Why? An explicit justification was evidently warranted. Every Wednesday Jüri, Ed and I met at the Courant Institute of Mathematical Sciences, where I was a Visiting Member. In the afternoons there would be a specialist seminar to a small audience of interested applied mathematicians and physicists: Steve Childress, Al Glassgold, Joe Keller, Peter Lax, Jürgen Moser, Sol Rubinow, Mal Ruderman, Englebert Schücking amongst others, all of whom could be highly critical in a very constructive way. I was to lecture on anelasticity. Usually the seminars took a little over an hour, but mine went on for three, as I fielded question after question, most of them around the issue of whether an independent background state could be defined. It took me two more three-hour sessions to convince them. Ed’s reaction was that surely I must now accept that the result is not obvious. So I published (Gough, 1969). The paper prompted many invitations to give seminars in meteorology departments.

The process of publishing that work raised an interesting issue. I had already refereed for the *Journal of the Atmospheric Sciences* a paper on a Boussinesq-like discussion implicitly making an expansion that did not conserve energy at each order. Even though the leakage was potentially no greater than the small expansion parameter, so that it could be argued that in some sense the expansion was formally not incorrect, a numerical computation of any truncated representation would be susceptible to gaining or losing energy artificially from the ignored higher orders, which could lead to artificial runaway. Spiegel and Veronis had taken care not to permit that to happen in their work, and I, having been made aware by Ed of the pitfall, had done likewise in my yet unpublished anelastic analysis. I suggested in my referee’s report that the author at least draws attention to that deficiency. I am never ashamed of what I write, and I readily revealed my identity. In this instance I thought that I was being helpful, but the author thought otherwise. Later, when I submitted my anelastic paper to the same journal, the editor, it transpired, sent it to the author of the paper that I had recently refereed, who chose, I presume, to appear not to understand my analysis, and ‘demanded’ changes that would have rendered it invalid. I had to ask the editor to send it to a competent referee, whom I subsequently learnt was George Veronis. He recommended acceptance. The experience made me appreciate how it can be inconvenient for a referee’s identity to be revealed. However, I ignored that until I experienced a much more serious repercussion:

I had refereed for *Nature* the famous paper by Severny, Kotov, and Tsap (1976) on the ‘discovery’ of the 160-minute oscillation of the Sun (and, several months later, the paper by Brookes, Isaak, and van der Raay (1976) on the same subject). It was in July 1975; I had received the manuscript only a few days before departing for a visit to Boulder, and, after settling in, and knowing the speed at which *Nature* likes a response, I sent copies of my report by post not only to the editor but also directly to Andrei Severny at the same time – this was before the days of the internet – explaining, so I thought, why my response was so tardy (I had taken 10 days or so to respond). Andrei and I had previously made friendly acquaintance during my first visit to the Crimea in 1969, and so I was quite upset when I received from him a curt response to my report, and I feared that our relationship had been damaged. Andrei wrote that he had carried out all my recommendations and that now he hoped that the inordinate delay that his paper had suffered would not be prolonged. The following autumn Nigel Weiss received a request from *Nature* to write a *News and Views* article on the two observational papers, which were to appear in the same issue; to my horror Nigel told me that the version of the Russian paper that he had been given had been received by *Nature* the previous March! Andrei must have believed that I had sat on his paper for four months. I have never felt able to set matters right. I complained vehemently to Philip Campbell, then the Physical Sciences editor of *Nature*, that referees should always be given all pertinent information about the history of the papers that they are sent, and he replied with an appropriate promise. Yet I resolved never again to reveal my identity as a referee unless I can anticipate a timely face-to-face meeting with at least one of the authors.

Of the other matters that I mistakenly believed were obvious, I mention here but two. The first is that gravity waves tend to enhance, rather than suppress, vertical shear in horizontal flow, and consequently enhance rotational shear in stars. This was clear to me from the GFD lectures by Francis Bretherton (1965) that I had attended at WHOI. Francis discussed the consequences of critical-layer absorption. It seemed obvious to me that a similar but more gradual process would apply anywhere, not just near critical layers (where the dispersion relation formally predicts zero frequency in the locally moving frame). The reason is simply that the frequency of a prograde gravity wave propagating vertically into a region of increasing velocity is Doppler-shifted downwards in the local frame of the fluid. Therefore, the vertical wavenumber increases, and consequently so does the dissipation rate, enhancing the deposition of (positive) momentum; retrograde waves behave conversely. The consequences can be complicated, and are now thought in particular to explain the quasi-biennial oscillation of the Earth’s atmosphere (Holton and Lindzen, 1972; Plumb and McEwan, 1978). Indeed, I once contemplated whether the process might be directly responsible for the solar cycle (Gough, 1998), an idea subsequently embraced briefly by Jean-Paul (Kumar, Talon, and Zahn, 1999), although I doubt that waves would be generated with sufficiently high amplitude to produce an oscillation period as short as 11, or 22, years. I had hinted at the phenomenon, too obliquely I now realise, in a discussion of internal solar dynamics at a conference in Nice (Gough, 1977a), but it has probably never been noticed. At the time, the only observational evidence for the existence of solar gravity waves was the infamous 160-min oscillation. Its amplitude was too low to be of much relevance, and I estimated that gravity waves generated at the base of the convection zone hardly penetrated into the radiative interior, so I didn’t pursue the matter further in the literature. Had I done so, scientists presenting calculations purporting to show how angular-momentum transport by gravity waves renders rigid the rotation of the radiative interior of the Sun would at least have been obliged to address the opposing view. I hasten to add that Jean-Paul Zahn and his colleagues have addressed the matter since (e.g. Zahn, Talon, and Matias, 1997; Mathis et al., 2008), although I do doubt some of their conclusions (e.g. Gough, 2002). That gravity waves penetrate only superficially into the radiative interior did not appear in the commonly read literature until Bill Press published it in the *Astrophysical Journal* (Press, 1981). I had made my discussion in Nice quite succinct, believing that the reader would be familiar with gravity-wave dynamics. Bill, however, reproduced much of the standard text-book material, which dominated what was presented to be a research paper. My Cambridge friends were astounded. But perhaps we were too old-fashioned, because Bill’s tactic seems to have been the more effective. That became even more apparent to me more recently when I mentioned to Jüri Toomre my thoughts on writing a book on helioseismology with Michael Thompson (Figure 12); Jüri considered it a waste of time on the ground that scientists no longer read books! I doubt him, but I can see whence he came, because Jüri works in an institute that no longer houses a library.

The other apparently not-so-obvious result is that in plane-parallel geometry the frequency $\omega$ of the fundamental gravity wave with horizontal wavenumber $k$ in a vertically semi-infinite fluid under constant gravity $g$ is given by the dispersion relation $\omega^{2} = gk$, irrespective of both the equation of state of the fluid and the stratification of its background state. I should stress that the restoring force producing the oscillation is basically gravity, so the associated resonant mode is indeed a g mode, not a p mode as some have labelled it, because it is uncompressed (even though the fluid may be compressible) and so is independent of the sound speed. The resonant standing wave is the fundamental g mode, which for a star was named f mode by Cowling (1941); its frequency approaches the plane-parallel dispersion relation in the limit of high degree, where the sphericity of the background state has no local influence on the dynamics. There exist very similar gravity modes, concentrated in regions of rapid density variation, that do depend on stratification (e.g. Whitham, 1974), which Colin Rosenthal and I studied more recently in the stellar context (Rosenthal and Gough, 1994), and that can have very similar frequencies. They are sometimes called interfacial modes. In the case of a terrestrial lake, a body of water supporting the atmosphere above (which is normally ignored), such waves having $k^{-1}$ much less than the depth of the lake are called deep-water waves.

It was as a result of the f-mode relation that I witnessed the mental agility of James Lighthill. I must have discussed the relation with someone in DAMTP who later had mentioned it to James. One morning at coffee time, James approached me to enquire of its justification: he told me that oceanographers were well aware of an augmentation of deep-water-wave frequencies by a stable density gradient caused by varying salinity, which can be calculated straightforwardly under the Boussinesq approximation; how therefore could I be right? I responded that what they had calculated was the product of error in the Boussinesq approximation, and started to explain why. After only a sentence or two James, no doubt relating what I was saying to all the facets of the subject with which he was intimately familiar, interrupted with a broad smile and said that of course it is quite obvious that I am right. So was the dispersion relation obvious to all? Not at least to solar physicists. Consequently, I was able to use it privately to assess the accuracy of published frequencies, both observed (e.g. Deubner, 1975) and numerically computed (e.g. Lubow, Rhodes, and Ulrich, 1980). Then one day I came across a publication by Jack Harvey and Tom Duvall (Figure 13) in which the f-mode frequencies fitted the theory perfectly. When next I saw Jack I asked him why his new observations were so much more accurate than the old. With a smile he said that he knew of the dispersion relation tucked away in a review that I had written for a conference (Gough, 1980) – he had been present at my talk – and that he had used it to recalibrate the angular scale on his telescope. As I describe in Section 27, the f mode plays an important role in establishing the orders $n$ of high-degree solar p modes. I record another unobvious obvious result in Section 31.

## Dirtying Hands, and the Solar Spoon

On my return to Cambridge Fred Hoyle triggered my involvement in the solar-neutrino problem. He asked me to carry out a computation of solar evolution with gravitational settling of helium, up to that time universally ignored, thinking that that might reduce the neutrino luminosity $L_{\nu}$. My back-of-the-envelope estimates suggested that the effect of settling would be to augment $L_{\nu}$, not reduce it, but Fred thought that sufficient displacement of hydrogen from the energy-generating core of the Sun might subtly modify the structure in such a way as to counter expectation. Fred also thought, mistakenly, that I was experienced with computations of the kind that would be required; I didn’t dare put him right for fear of jeopardising the renewal of my appointment, for John Faulkner had already left for Santa Cruz and maybe Fred had hired me as a poor replacement. Fortunately, Bohdan Paczyński had left a stellar-evolution code at IoTA for general use. It was cumbersome to operate because it was running on a computer with only 175 kB of legally accessible memory, and therefore had to be run in stages, each stage punching its results onto cards to be read by the succeeding stage. Yet it was so clearly written as to be immediately understandable, and with some advice from Peter Eggleton I was able to modify it to accommodate gravitational settling and add the neutrino-flux computation before Fred might chase me up wondering why I was being so slow.

Before reporting the outcome, I record a very important lesson that the experience taught me. An obvious check that I needed to make was to compare my value of $L_{\nu}$ in the absence of helium settling with previously published values. My computations were made long before the days of the useful reviews that John Bahcall was to publish later, so I had to search the literature for nuclear-reaction cross sections, and adopt from amongst the variety of values those that seemed to me the most likely. I was disappointed to find the resulting neutrino luminosity to be about 20 solar neutrino units (snu), some three times greater than the 6 – 8 snu that appeared to be the preference of the day based on the ‘most likely’ cross sections (e.g. Bahcall, Bahcall, and Shaviv, 1968; Bahcall, Bahcall, and Ulrich, 1969), even though mine was similar to the value that Ray Davis Jr (1964) had adopted in the design of his experiment (actually Ray’s was twice my value, but that was before there had been a dramatic increase – by a factor 5 – in the experimental determination of the $^{3}{\mathrm{He}}$–$^{3}{\mathrm{He}}$ reaction cross section, which reduced the theoretical value of $L_{\nu}$ by some 30%). So I tried varying the cross sections arbitrarily, and the chemical composition, within plausible bounds. I found that $L_{\nu}$ varied enormously, yet oddly enough I could not reduce it below about 6 – 8 snu. Presumably, before there were solar-neutrino data to guide them, other theorists must have been misled similarly. The lesson, at least for me, was clear: to obtain a real appreciation of the results of someone else’s complicated numerical calculation it is necessary to get one’s hands dirty by performing a similar calculation oneself. I learned later, studying analyses of the effect of a magnetic field on stellar-oscillation eigenfrequencies, that the lesson applies to complicated analytical calculations too. The outcome of my exercise was as I expected: the effect of the increased central condensation of solar models produced by the settling was generally to augment, not diminish, $L_{\nu}$, but by only about 10%. That disappointed Fred, which perhaps explains why he never again asked me to carry out a calculation for him.

A second lesson that I learned from this experience is that the relatively smooth modification that arises from a small perturbation such as gravitational settling causes the radiative interior of a solar model to respond almost homologously in global variables such as pressure and temperature, which, unlike density for example, occur most directly as spatial derivatives in the equations of stellar structure. Moreover, the response of the outer convective envelope of any Sun-like star can also be estimated by appropriate homologous scaling, this time with respect to depth rather than radius, and adjusted to match onto the interior (cf. Christensen-Dalsgaard and Gough, 1998). Deviations from homology in more locally determined variables such as density can be estimated by local power-law scaling, which is tantamount to linearising the deviation in logarithmic variables. Indeed, complete linearisation is even better (e.g. Gough, 2003). Therefore, the outcome of introducing some change to the modelling can be estimated without recourse to a full numerical evolutionary calculation. This has been particularly useful in reconciling neutrino-flux estimates from the many perturbations that have been made to solar models. The broad outcome from scaling is not always in agreement with a full calculation, and sometimes I have argued in favour of the former, such as my questioning (Gough, 1992) a result of the Yale Rotating Evolution Code (YREC) that Marc Pinsonneault (1992) had described. It was thinking along such lines that led me to realise that the solar-neutrino problem could not be resolved merely by tinkering with standard solar models, and that if the resolution lay in solar modelling a more dramatic modification would be required. It also led to my deriving a simple formula for the main-sequence variation of the Sun’s luminosity (e.g. Gough, 1977b; Gough, 1990b)^9^ that, according to Roberto Terlevich, in a lecture in Mexico City was flatteringly named “Gough’s Law” (cf. Angulo-Brown, Rosales, and Barranco-Jiménez, 2012).

As soon as the two-year penance of visiting scholars to remain away from the US was over, Ed invited me to Columbia for a seminar. Solar neutrinos were a hot subject, and since I now had some feel for the issues, I announced a pregnant title. I had been pondering the limitations imposed on the nuclear-reaction rates by the constraint that the Sun is in thermal balance, and wondered whether a gravity mode that is rendered unstable to Eddington’s $\epsilon$ mechanism as a result of the accumulation of $^{3}{\mathrm{He}}$ in the core might grow in amplitude sufficiently to break, upsetting thermal balance and mixing the contents of the core, thereby destroying the destabilisation that initially triggered it until adequate further $^{3}{\mathrm{He}}$ accumulation has again taken place. The calculation was in two stages: first the g-mode instability, second the Sun’s response. I had never calculated a non-radial stellar oscillation before, and since I was up against a tight deadline I opted to use Tom Cowling’s adiabatic g-mode eigenfunctions of a polytrope (Cowling, 1941), computing the nonadiabatic effects as small perturbations. Because g modes oscillate on a timescale much shorter than the time for the p–p and the $^{3}{\mathrm{He}}$–$^{3}{ \mathrm{He}}$ reactions to equilibrate, the temperature sensitivity of the total energy-generation rate is an appropriate average of the sensitivities of the two, rather than the much weaker sensitivity of just the p–p reaction that controls the energy generation on the evolution timescale, a situation that, as I learned later, Ledoux and Sauvenier-Goffin (1950) knew well. I estimated that to be adequate to destabilise the modes after sufficient $^{3}{\mathrm{He}}$ had accumulated would take about 250 million years. The presumption then was that the non-linear breakdown of the mode mixed the contents of the core, destroying the $^{3}{\mathrm{He}}$ gradient and thereby quenching the disturbance for another 250 million years. To carry out the second phase of the project I used Bohdan’s stellar-evolution programme, although I had to modify the integration scheme to cope with the extremely rapid temporal variation consequent on the redistribution of chemical species. The outcome was a sudden decrease in the surface luminosity, by about 5 per cent, resulting from an essentially adiabatic expansion of the envelope, followed by thermal relaxation on the $10^{7}$-year Kelvin–Helmholtz timescale, $\tau_{\mathrm{KH}}$, and a temporary substantial reduction of $L_{\nu}$. I was delighted, and of course I told Fred, who surprised me with his reticence. Noticing that the interval between such disturbances more-or-less corresponds with the interval between major ice ages, I interested one of my students, Fisher Dilke, to search the literature to see whether or not the geological records bear this out. The crucial point was that because the Earth is in the throes of an ice age at present, the neutrino flux is currently anomalously low. The story seemed to me to be more plausible than any other I had heard amongst the astrophysical explanations that had been proffered. Now I had material enough to deliver my seminar.

The response at Columbia was enthusiastic. Ed advised me to publish as soon as possible because word would get around quickly. After discussing the geological issues with a colleague, Nick Shackleton, Fisher and I wrote a paper for *Nature*. We called it the solar spoon. Soon after we submitted there appeared a discussion by Willy Fowler (1972) about the response of the Sun to possibly periodic disturbances in the core and their effect on $L_{\nu}$, which explained Fred’s reticence. However, Willy had no idea as to what might drive the disturbances, nor did he appreciate that the modifications they cause to hydrostatic balance produce an essentially instantaneous (i.e. after only an acoustic travel time) response at the surface. The following year Fisher and I submitted an essay on the subject to the annual competition held by the Gravity Research Foundation. It was an impertinent act, because eligible essays should have been about theories of gravity – we simply put the word gravity (wave) in the title. So we were duly astounded when we were awarded second prize. Most interesting of the reactions to our *Nature* paper were from Martin Schwarzschild and from Paul Ledoux. Martin wrote to me declaring his interest in working on the g modes himself; I replied pointing out that Fisher was also working on the subject, and Martin, perfect gentleman as he was, said that he would not compete with a student and would therefore wait a year. Paul, another perfect gentleman, also wrote, saying that he was going to review the work at the IAU Symposium on *Stellar Instability and Evolution* to be held in Canberra the following year.

I was quite surprised later when reading his review (Ledoux, 1974) that Paul seemed to doubt the veracity of Fisher’s and my conclusions, yet he didn’t explicitly contradict us. When next we met I asked Paul why he had written so obliquely, and he told me that he had repeated what he thought had been our calculation but didn’t find the instability that we had claimed. He wasn’t so crass as to point it out explicitly. It took a great deal of discussion before we could establish the root of the disparity: I had used the whole of Cowling’s displacement eigenfunction in the work integral to determine the instability, because normally that is the most natural approach, rather than adopting only its vertical component combined with the pressure perturbation. Paul had used his own eigenfunctions, because he knew that the horizontal component in Cowling’s paper is misprinted. It hadn’t crossed his mind that I didn’t know that too. “It’s pointed out in a footnote in my article with Théadore Walraven on Variable Stars in volume 51 of the Handbuch der Physik”, he told me after we had resolved the discrepancy. So I of course resolved to sleep with that volume under my pillow.

In 1982 a conference on *Pulsations in Classical and Cataclysmic Variable Stars* honouring Paul Ledoux was held in Boulder: Paul was not only a fine scientist and a gentleman – indeed he reminded me of my Italian grandfather – but he was also a wine connoisseur, and was respected by all who knew him. After the meeting I took Paul out to dinner; he wanted to pay for at least the wine, but I forbade it. In retaliation he threatened to have me to dinner at his home and sample the contents of his renowned cellar when next I visited Liège, to which I was certainly willing to acquiesce. Sadly, Paul died before my visit. I learnt of the death from Maurice Gabriel immediately before the October 1988 meeting of the Royal Astronomical Society. It was then normal under such circumstances for the Society to stand for a minute in silent respect for deceased distinguished Fellows, and I hastened to request it of the President, Roger Tayler. At first Roger was reluctant, which was quite uncharacteristic: he then told me of an earlier occasion when the meeting was asked to stand and a small voice piped up: “Am I supposed to stand too?” On this occasion I was able to assure Roger that Maurice was reliable, so the gesture was indeed made.

**Figure 6:**
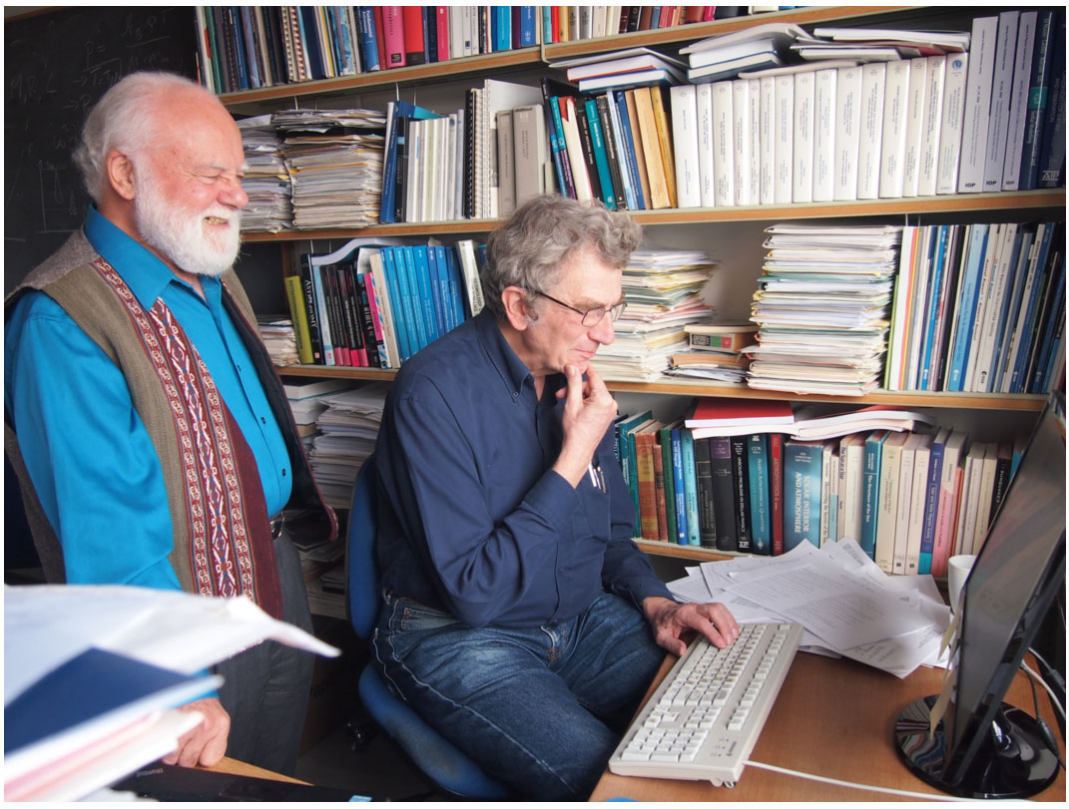
A recent photograph with Jørgen. Photo: Birte Christensen-Dalsgaard.

Paul’s revelation of Cowling’s error has made me wonder whether I would have subsequently embarked with Fisher on the g-mode instability calculation of a realistic solar model had our polytropic calculation been carried out with the correct eigenfunctions. In the event, Fisher and I did not complete the calculations to my satisfaction by the time Fisher was to graduate; most fortunately a new student, Jørgen Christensen-Dalsgaard (Figure 6), arrived on the scene, and with the knowledge that we might now be racing Martin Schwarzschild, he bravely joined in the completion of the project (Christensen-Dalsgaard, Dilke, and Gough, 1974). Those calculations were so-called quasi-adiabatic: linearised nonadiabatic corrections, via a work integral, were made to adiabatic eigenfunctions for the purpose of computing growth rates. Later, for reassurance, we succeeded in computing nonadiabatic oscillations (ignoring perturbations to the turbulent momentum, enthalpy and energy fluxes). To ensure numerical stability, alternate backward and forward differences were used to represent derivatives in the energy-transport equations just in regions where nonadiabaticity is negligible, and that progressed continuously to centred differences elsewhere where accuracy matters, a technique that I had used previously in early pulsation computations with Norm Baker (Figure 12) in New York. We found a severely damping region immediately beneath the photosphere, but its affect on the mode stability could not be trusted because the convective-flux perturbations had been ignored (Christensen-Dalsgaard and Gough, 1975). According to John Cox (1980), those were the first ‘fully’ nonadiabatic global calculations of non-radial stellar oscillations ever to have been performed.

Those calculations marked the beginning of a continuous exhilarating collaboration and a lifelong friendship. Jørgen and his wife Birte afforded me the honour of being godfather to their first daughter, Karen.

## Consequences of the Spoon

The process of publishing the original paper (Dilke and Gough, 1972) was interesting. We had a lot to say, and despite our attempts at concision the length of the paper exceeded *Nature*’s guidelines. The referees reported favourably, the more effusive one even asking for substantially more detail in several places; the editor’s response required us to accommodate the referees’ requests, yet at the same time shorten the paper by 30%. How could that be possible? We wrote back enquiring, and two of the editors offered to rewrite it. The new version was only slightly shorter, by less than 10%. We disliked the outcome, for it had lost much of the essential detail. But evidently it defined an acceptable length. So we rewrote the paper within that length, and, of course, *Nature* had no alternative but to accept. It was four-and-a-half pages long, which *Nature* disguised by splitting it into two, with some thirty pages between the two parts.

I share with Paul Ledoux his attitude towards only hinting at mistakes in the literature. Unfortunately, those hints often go unnoticed, and sometimes I have been wrongly accused of being unaware of pertinent details, or of not having proved a statement that I have written. Yet there have been occasions on which I have spent days justifying a single sentence to myself before committing it to paper. Such hinting is no doubt not the best way to disseminate information, as at times my referees have complained. A notable example was in a joint paper on the convective instability of a compressible atmosphere (Gough et al., 1976b) in which we were bound to address why our critical Rayleigh numbers differed from an already published result by as much as a factor 2. In the original version we pointed out only very briefly, yet precisely, how we knew that the published calculation was wrong; my co-authors wanted the argument to be spelled out in greater detail, but I argued for a softer approach, it being more polite. However, the author of the erroneous paper was our referee, and he thought otherwise. He objected to our claim, confessing his inability to understand its succinct justification. We expanded little by little. It took several iterations before we were explicit enough to leave no room for doubt. We explained the referee’s blunder privately to him, and at least he agreed to our not exposing it fully, for this was a paper about physics, not about the mathematical aspects of singular points of differential equations that was oblivion to him.

Justification of the argument that Fisher and I had advanced for the timing of the last putative overturning of the Sun’s core had not been established. The immediate (i.e. on a timescale $\ll\tau_{\mathrm{KH}}$) response of the Sun is a reduction in the neutrino flux by a factor of about 4, and a reduction in the luminosity by about 5 per cent. Is that sufficient to induce terrestrial glaciation? According to standard solar-evolution theory, in which there is no mass loss, the luminosity has risen from about 70 per cent of its present value in $4.6 \times10^{9} {\mathrm{y}}$, yet the Earth has not been glaciated for most of that time. The disparity of timescales is crucial. I spent a lot of time during the summers of 1972 and 1973 at the National Center for Atmospheric Research (NCAR) in Boulder discussing the matter with climatologists. Notwithstanding the fact that NCAR’s Global Circulation Model (GCM) of the Earth’s atmosphere is designed to operate over only short, meteorological, timescales, I went to see Stephen Schneider, who was in charge, and asked him if the solar constant could be turned down by 5% to answer an important question. He rebuffed me, saying that it would be a waste of valuable resources: “The solar constant wouldn’t be so called if it were not constant”, he retorted. Then I encountered his postdoctoral assistant, Tzvi Gal-Chen, who actually ran the GCM each night. He was intrigued by the proposition; encouraged by Ann Henderson-Sellers and her husband Brian, he illicitly reduced the solar constant, and reported the next day that the Earth had indeed become glaciated. Stephen was furious. I was delighted. Yet the next day was even more interesting: Tzvi told me with some embarrassment that he had forgotten to arrange the start of the next night’s computing from the unglaciated model of two-nights earlier, and that the restored solar constant had failed to melt the ice. Evidently, either the standard solar model is incorrect or the GCM is inappropriate.

The naive assumption that the lower solar constant throughout much of the main-sequence history should have led to a glaciated Earth was discussed in some detail by Sagan and Mullen (1972). It was dubbed the faint Sun paradox. Some scientists believed the only plausible reconciliation to be that the natural tendency for the Sun’s radiative output to increase has been miraculously compensated by mass loss or a decline in the gravitational constant. Sagan and Mullen proposed that changes in terrestrial atmospheric composition, dominated by a decline in the concentration of ammonia most likely due to oxygen released by green-plant photosynthesis, has magically caused the Earth’s albedo to increase in step with insolation. However, there was good reason to believe, as I reiterated recently (Gough, 2015), that the GCM ‘prediction’ of long-term subluminal glaciation was flawed, because it does not account adequately for changes on climatological timescales.

Another consequence of the solar spoon was the interest it sparked in George Cowan, who invited me to Los Alamos, where he was a perennial visitor, to discuss a possible experimental test. According to my calculations the temporal variations of the various neutrino-producing reactions in the Sun differed, and by measuring the abundance ratios of different isotopes of technetium produced by energy-dependent neutrino capture by molybdenum one could obtain two different integral measures of the neutrino flux on Earth, averaged over timescales of 1 and 4 million years (Cowan and Haxton, 1982). Sadly, George did not survive to supervise the project, which in any case was dropped because it transpired that it would have been too expensive to purify the technetium to an adequate degree. An event that I still recall from that visit was my flight in a small plane along the valley leading up to Los Alamos. There were just two of us passengers. At some point I was startled when the pilot turned around to my companion to point out a landmark: it was where on a previous occasion the pilot, who had fallen acutely ill and feared for the safety of his one passenger of that time, had given him the only parachute and told him to jump. This time there were two of us, yet possibly still only one parachute. It strengthened my opinion from student days that experimental science is not for me. However, as I shall report later, that did not stay my curiosity.

Whilst on the subject of flights, I recall also my privilege of having been in a front seat on several occasions. The first was on an early-morning flight from Albuquerque to Alamogordo, on the way to Sacramento Peak. I was the only passenger, and was invited to sit in the co-pilot’s seat. As we were flying alongside White Sands, the pilot started looking around, apparently nervously. “Have you ever seen Trinity?” he enquired. “No” said I. He told me that it was illegal to fly over the missile range, but that the US Air Force appeared not to be firing missiles that day and would not see us; he dipped the starboard wing and flew over the crater. By chance, when I flew home some days later, I had the same pilot. There were about a dozen passengers this time, and again I was invited to the co-pilot’s seat. It was a hot mid-day, and to temper conditions in the cabin I was asked to hold open my door as we taxied down the runway. That was no doubt illegal too, but I was well strapped in, and, once the engine was on full power, to hold open the door would no longer be possible. A third experience was flying home to Boulder from the Grand Canyon with a friend, Katherine Gebbie, in her small plane. On both the outward and the return flights she allowed me to fly the plane, except, of course, when taking off and landing. It was an exhilarating experience. On the way home we went over the Meteor Crater in northern Arizona: Katherine said that all I needed to do was to dip my starboard wing to point to the middle of the crater and we would automatically circle it. One loses height during such a procedure, so after a suitable interval Katherine calmly suggested that I straighten up. However, I made a sign error! The shock of suddenly seeing the horizon vertical when I looked again through the windscreen remains imprinted in my memory to this day: my instinct told me that I would “fall” forever into nowhere, and my stomach almost reached my throat. We were high enough above the ground for me to correct my mistake. After that I did succeed in flying back to Boulder, but I felt extremely queasy until my feet were again on solid ground.

In the light of my recovery from the crater spiral, Katherine suggested that later I take her plane into a downward spin, and recover from it. She did not feel confident to accompany me, but recommended an experienced instructor who she was sure would oblige. I was enthusiastic. But my family were not.

Some time after the publication of the solar spoon, Wojtek Dziembowski (1982, 1983) took up the matter, arguing that because direct diffusive damping is small, pairs of daughter modes could exist that resonate precisely enough with the original g mode to sap its energy via triad interaction, at least in a non-evolving star. He estimated the expectation of the g-mode amplitude, finding it to be too small to have a significant influence on the structure of the Sun. Much later, my student Chris Jordinson and I repeated Wojtek’s calculation, taking into account the temporal structural changes associated with the solar cycle, and merely reported (Jordinson and Gough, 2000) that long-term phase coherence is not necessary to effect efficient energy transfer to the dense spectrum of daughter pairs: as the Sun’s stratification changed over the cycle, different triads successively fell into resonance, rendering Wojtek’s expectation a near certainty. We did not publish the details because by then it was no longer of general astrophysical interest, for it was already clear from helioseismology that substantial mixing of material in the Sun’s core has not taken place. However, there was, and still is, a seismological hint that there has been some slight mixing (e.g. Gough and Kosovichev, 1990; Gough, Kosovichev, and Toutain, 1995).

## The Institute of Astronomy

The original grant that supported IoTA was due to run out in August 1972. There was a proposed merger between IoTA and the university’s Observatories, which eventually took place, and the university, in its unfathomable wisdom, decided without informing Fred Hoyle that he was not to be its Director. When Fred heard of the decision, from a senior administrator in the Science Research Council (SRC) on a flight to Australia, he resigned from his Plumian Chair (actually for the second time, but on this occasion with an artificial irrevocable reason), although he remained in Cambridge for some weeks until at least some interim funding had been promised by the SRC. This was decided on my thirty-first birthday, by chance, but initially for only one year. For the few young IoTA staff who remained (others had seen the writing on the wall and had accepted posts elsewhere) there was a long period of silence from the university. Then, in November 1972, more than three months after our appointments had expired, we received letters (written in the future tense) offering us one-year appointments starting on 1 August 1972 under the condition that we relinquish all employment rights under the law. Being otherwise unemployed, we acquiesced. There followed negotiations between Donald Lynden-Bell, the newly appointed Professor of Astrophysics, George Batchelor in DAMTP, and other university administrators, which ended (after considerable horse-trading) with the establishment of a lectureship joint between DAMTP and the institute, now to be merged with The Observatories, the promise that the next vacant lectureship in DAMTP would be assigned to a (broadly interpreted) astrophysicist, and a permanent new, less senior, research post at the merged institute, together with some long-term arrangements not all of which finally materialised. There were just five of us young temporary staff who had a possibly realistic aspiration to remain in Cambridge, but there were to be fewer imminent posts. I recall having long conversations with Brandon Carter and Stephen Hawking about our futures: Stephen saw a possible solution for Brandon and me as taking up the two lectureships, but, because his declining health already rendered it impossible for him to lecture, he was deeply worried about how he and his family would survive. Brandon and I protested that because Stephen was so much brighter than both of us put together somehow the university was bound to solve his problem, but that allayed none of his fears. In the end Stephen was indeed accommodated, at first by his college; Brandon was appointed to the lectureship in DAMTP and I to the joint lectureship; Peter Eggleton to the research post. There remained Sverre Aarseth, the most senior of us five, for whom was found ‘temporary’ funding that in the end was renewed continually for the three decades or so until his ‘retirement’ (he is still active in research). Subsequently, Brandon moved to Meudon, and Stephen became Lucasian Professor, a post once held by Newton. My appointment was to the post I most wanted: I wanted to maintain links between astronomy and applied mathematics, and now I was to be a member of both departments with the opportunity to do so; I also wanted to teach. A few years ago I was clearing out some filing cabinets and stumbled upon my application for that post, and was amazed at how sparse it was compared with the applications that one encounters today. I suppose that others at that time were similar. Once the merged institute was established, the staff named it democratically Institute of Astronomy (IoA).

Initially, IoA was a purely research institution, so my teaching was entirely in DAMTP. Later, after several years’ negotiation I succeeded in introducing astrophysics as a full-time final-year option in the Natural Sciences Tripos. Together with Jim Pringle, then a recent addition to IoA and also one who appreciated the importance of educating students, we wrote the detailed syllabuses for the 182 lectures, a prerequisite for acceptance by the university. The course was successful, and some years later we were able to add a year’s graduate course. I enjoyed the consequential added scope to my own lectures, although eighty per cent of my teaching remained in mathematics.

By this time I had become a Fellow of Churchill College, despite some opposition to my election. That was before my lectureship appointment. As soon as I had taken up the fellowship, David Kendall, Professor of Mathematical Statistics, admitted that it had been he who had been the opponent, but insisted that it had been my temporary status to which he had objected, not to me personally nor to any of my credentials; he did not want the college to have to go through another search. Fortunately for me, Nigel Weiss, a tenured lecturer in DAMTP, had been sought for advice, and estimated the likelihood of my being appointed to the mooted joint lectureship. That satisfied David. He and I immediately became friends, and remained so until David’s death thirty-five years later.

One of my several imprudences of those early days in Cambridge arose from my finding myself immediately after the return of Apollo 11, by means unbeknown to me to this day (although probably by having recently published a paper with John Bastin in 1969 on the thermal properties of the Moon’s surface layers), the courier of a sample of the Moon’s regolith that I had been charged by NASA to guard with my life and deliver to the geology department in Cambridge for analysis. The date of my travel was not disclosed, so I could safely wait a day or so before delivery. That provided me with the opportunity to hand it to my four-year old daughter Karen, whom I knew could be trusted, to take to school for a day for her teacher to show the class. It would obviously be of supreme educational value to the children. Naturally, Karen returned it home safely. Come to think of it, I would entrust my life to any of my children, so my act was hardly an imprudence.

## On Computing

I have a love–hate relation with computers. The hate arose from my attending a computer course as an undergraduate. The first half of the course was numerical analysis, which I found to be inordinately boring: studying complicated algorithms to enable one to achieve certain ends with supreme accuracy from a minimal number of operations on a hand calculating machine. The second part used EDSAC II, the university’s home-made electronic computer. In a one-hour session each member of the class had to write a simple programme that was then punched onto tape and submitted to the machine. Usually there was time for only one submission, so the programme had to be correct. In the first two weeks we wrote in machine language; that was long-winded but straightforward, because every instruction had precisely the same syntax: subject-verb-object. I managed a few calculations. In the second two weeks we wrote in a high-level language, EDSAC autocode, which was supposed to be easier and quicker. But I didn’t succeed in getting a single programme to run, because each time I had slipped up by having forgotten a comma or used a colon instead of a full stop. Things don’t change.

When I became a research student I had to solve an ordinary differential equation to determine the convective stability of a polytrope pervaded by a uniform magnetic field. By now the university had purchased a commercial mainframe computer that accepted FORTRAN. I acquired a primer, went to an outdoor swimming pool – it was summer – and spent the day reading it. Then I wrote my programme. I cannot remember how long it took to debug, but I do remember that FORTRAN turned out to be enormously more accessible than the autocode. Once I was sure that the programme was running correctly, I submitted the culminating job of my student career: solving a differential eigenvalue equation for a sequence of controlling parameters. It took about twenty minutes to execute, during the night of course, and for each solution I had printed only one line, containing just the defining parameters and the eigenvalue. The next morning I retrieved my one page of line-printer output, on which was written a stern note from the operators to the effect that I should have printed much more from so much computing.

It was not until I went to New York that I computed (seriously) again. I wrote a programme to solve a partial differential equation describing thermal conduction in the Moon, with complicated radiative conditions at a rough upper boundary that in some places reabsorbed heat radiated from elsewhere on the surface. My interest in the subject had been aroused by John Bastin, whom I had first met at JILA. John was keen to explain lunar infra-red observations, many of which he had made himself. He believed that the key was surface roughness on a centimetre scale, so we set out to demonstrate it (Bastin and Gough, 1969), and to do so before the first Moon landing. We became good friends; later John took on the responsibility of being godfather to our second daughter, Heidi. With the Moon landing came what experts considered to be the surprising discovery of high-Q lunar seismic waves. I did some calculations of propagation through the regolith, accepting that the lack of erosion, and the lower gravity, led to a material of particles more rigidly connected than in terrestrial soil, and therefore less dissipative. Unfortunately, there were insufficient selenoseismic data for me to draw structural conclusions.

Computing in New York was very different from what I had experienced in Cambridge. Now I had the use of a world-class machine. Matters then became even more serious with the delivery to GISS of an IBM 360/95, the biggest computer in the world, and the only one of its kind. It had 1 Mb of memory (later augmented to 3 Mb) and took a month or so to install. Then, the IBM engineers defied the staff to make it crash, offering a prize of 50 cents to the first. I took my Moon programme downstairs, and crashed the computer straight away. It was some weeks before the system error was identified and corrected. Then the challenge was repeated, and I caused a second crash, with the same programme. I was now proudly richer by one whole dollar. The second error was repaired more quickly, and after the third challenge my programme ran without further incident. The computer had cost six million dollars to buy, I believe, and even more to manufacture, so I was somewhat disappointed with the $1 value that IBM had assigned to its reliability. IBM made one more 360/95, to satisfy a prior commitment, but never another.

During my stay at GISS I also wrote a general non-linear-ordinary-differential-equation-solving package, which I called NRK.^10^ The original purpose was to solve the single-mode convection equations being studied with Ed and Jüri. It evolved from the programme used by Norm Baker and Rudi Kippenhahn to study stellar pulsation, using a procedure invented by Leon Lucy to iterate simultaneously on eigenvalues and centred second-order-accuracy finite differences representing derivatives. My generalisation incorporated integral constraints, and could cope with internal and boundary singular points. It was later adopted by Dan Moore in the Mathematics Department of Imperial College as a teaching facility, and induced Dan and his colleague Jeffrey Cash to write a fourth-order-accuracy, finite-difference formula (Cash and Moore, 1980) that could easily be incorporated into it. Subsequently, Jørgen Christensen-Dalsgaard and I generalised NRK to solve initial-value, time-dependent problems with an application to stellar evolution principally in mind (Christensen-Dalsgaard, 1982);^11^ it remains the heart of Jørgen’s stellar-evolution code.

On my return to Cambridge I should have had to manage with only the 175 kB of legitimately accessible computer memory of the IBM 360/44 at IoA, in enormous contrast to the 3 MB at GISS. That required seriously rewriting NRK to economise on space. Now, matrices are frequently overwritten, rendering the programme very difficult to read. Such manoeuvres are unnecessary today, but after having tested the programme exhaustively it would be madness to attempt simplification: if it works, don’t fix it. There was just one procedure that I did have to remove: to generate more memory space, I overwrote almost the entire operating system, after first having written it to disk, leaving me free to use almost the entire 200 kB of the machine subject to leaving intact the read and write controls for disk transfer. In those days the operating system was unprotected, and I could accomplish that dangerous act in FORTRAN. Nick Butler, the computer manager, reacted explosively when I suggested it to him, but I did so only after the event, which I had achieved without crashing the system. So Nick and I remained friends. By then Rosanne had just given birth to our third child, this time a son, Julian, to whom Nick became godfather. Julian is the only one of our children to become a scientist, now researching in bioinformatics.

The experience I gained in circumventing many of the limitations of the computer caused me to be recognised as some kind of (renegade) expert. I found myself spending much of my day advising others, to the extent that I was often frustrated by how little time remained for research. In 1977 – 1978 I had sabbatical leave in Nice, and on my return the IoA had moved to a DEC computer running the VMS operating system. Although the new system was enormously more intuitive, I took great care then, and forever afterwards, to make quite sure that I understand operating systems barely enough to carry out the relatively straightforward tasks with which I am confronted, and no more. The IoA now has an excellent professional computer service, which is a great improvement for everyone.

## The Dawning of Helioseismology

In June 1975 I organised a conference, funded by IBM, on astrophysical fluid dynamics. Participation was by invitation. The normal one-week agenda were spread over two weeks: mornings only, leaving the afternoons free for unscheduled personal discussion. I was not unduly concerned over possible lapses in conversation because all the participants had been carefully picked. One was Martin Schwarzschild, who, I learned, had an enviably robust constitution: I bumped into him walking through my college grounds late on the Sunday morning before the meeting was to begin; he informed me that he had just arrived from Princeton, that he was about to go to bed for a few hours before my welcoming reception that evening, and that then he would be fully prepared for the fortnight to come: “I will have caught up with my sleep”, he said, “I need 56 hours a week, but it doesn’t matter when I get them”. How wonderful to be that flexible. One afternoon during the meeting I was discussing convection with Martin and Ed Spiegel over tea in the famous tea-gardens in Grantchester, a village just south of Cambridge, when Martin expressed his delight in seeing a finch, one not seen in the US, which he misnamed. “No, it’s a chaffinch”, I countered, pointing out the two characteristic white wing-bars. Little did I know that Martin was an expert ornithologist. In fact, he was to spend a week bird-watching in Iceland on his way home after the meeting. Had I realised I would never have dared correct the great man for fear of making a mistake myself. Later, Ed told me that Martin, somewhat surprised, had consulted his books as soon as he returned home, and had found me to be correct. As a result I had gained his respect. It’s a pity that it was not for my astrophysical insight.

Ed told me about an interesting man from Arizona who was visiting Paris and who had, Ed had heard, made some fascinating observations of oscillations of the Sun. Immediately I invited him to come to Cambridge to address the conference. The advantage of having a conference with so loose a schedule is that there is room for such last-minute insertions. The man was Henry Hill. He arrived a couple of days later, on 19 June, and addressed the meeting with a stunning description of an extensive spectrum of what appeared to be acoustic oscillations. Immediately I asked Jørgen to run our g-mode computer programme at higher frequency. I was too busy with the conference organisation to do it myself; in those days I had no secretarial assistance. The next day Jørgen gave a talk comparing Henry’s frequencies with our theory: it seemed very plausible that Henry was observing low-degree p modes that penetrated into the core of the Sun. The prospect was thrilling. Coupled with a spectrum of high-degree p modes that were rumoured to have been observed by Franz Deubner (1975), it seemed not unlikely that in the not-too-distant future frequency measurements would be extensive enough and precise enough for the stratification of the Sun to be determined right to the very centre. That was, for me, the dawn of helioseismology. Should I now be optimistic and risk spending time trying to develop helioseismic inverse theory, in a manner similar to that being pursued by geoseismologists, anticipating much wonderful data? I talked about it with my friends. Mike McIntyre (Figure 12), particularly, encouraged me to write an anticipatory paper to *Nature*. I was reticent because there were yet no results. But later I received the paper by Severny, Kotov, and Tsap (1976) on the 160-minute oscillation of the Sun to referee for *Nature*, and took that as a cue. In consultation with Jørgen, I wrote the paper that summer whilst participating in an Advanced Study Program at NCAR, and sent it in (Christensen-Dalsgaard & Gough, 1976). The NCAR library doesn’t have a copy of the authoritative Greek lexicon by Liddell and Scott (1889), just a small dictionary that translates $seismos$ imprecisely as earthquake. So I coined the word heliology instead. Our paper took four months to appear, during which time I was sent a paper to referee by Brookes, Isaak, and van der Raay (1976), also on the 160-minute oscillation, and was able to incorporate it; all three papers were published together. On returning home I consulted Liddell and Scott to learn that I could have adopted my favoured term helioseismology. So I used that term thereafter, although not at first in print, because it was opposed by Jüri who believed that American grant-awarding politicians would not understand such a term. I started thinking about how to analyse seismic data to address the two most important issues in heliophysics: the gravitational oblateness and its impact on General Relativity, and the so-called solar-neutrino problem.

The following spring I pondered the implications of the discrepancy between the computations of high-order acoustic oscillations by Ando and Osaki (1975) and Franz Deubner’s (1975) solar observations. The discrepancies were relatively small, so I could apply perturbation theory, using a crude polytropic approximation to the outer layers of the convection zone to estimate the adjustment required of the theoretical model of the Sun. That provided a measure of the outer stratification: the jump in what is essentially entropy across the upper convective boundary layer. Fortuitously, Nigel Weiss and I had just established the relation between that jump and the depth of the convection zone in an investigation of the differences between various representations of mixing-length theory (Gough and Weiss, 1976). By combining the two calculations I was able to infer that the Sun’s convection zone, which, as I discuss later, was favoured to be quite shallow by those trying to rationalise the neutrino problem, is actually some 50 per cent deeper than in the preferred models of the time. I reported that conclusion the following summer at IAU Colloquium 36, held at l’Observatoire de Nice, offering 200 – 250 Mm for my estimated depth, based also on hints of lower-frequency oscillations which have not been conclusively confirmed. Several people commented that Peter Gilman in particular, who was not at the conference, would be pleased because it was only if the convection zone were deep that he could produce equatorial acceleration in his models of the solar convection zone. Writing up my presentation was a nightmare, because the principal editor, Philippe Delache, desirous of rapid publication, telephoned me, and presumably all the other authors, two or three times a week demanding to be told exactly what progress had been made. I was teaching nine hours a week, and had various other commitments, so finding time was not easy. Sometimes, I was moved to invent progress that I had not yet made, which meant that I had to work even harder before the next phone call. Philippe must have been on the phone almost continuously that autumn, but he succeeded in getting my manuscript, handwritten, well before the end of the calendar year. He arranged for it to be typed in Nice, but never showed it to me for fear of undue delay. The outcome was many more errors in the photocopied published paper (Gough, 1977a) than I feel comfortable with. Nevertheless, I truly admire Philippe for his dedication. Soon after I sent in my manuscript I heard through the grapevine that Roger Ulrich was also trying to calibrate a model of the convection zone. Perhaps he too had an estimate of the depth. So I wrote to him, for it might not have been too late to add his result to my discussion. However, Roger replied that he did not yet have a value. So far, mine appeared to be the only seismological inference about the interior structure of the Sun. Quite soon afterwards Roger, in collaboration with Ed Rhodes and George Simon (Rhodes Jr, Ulrich, and Simon, 1977), did obtain an estimate: 180 – 270 Mm, which was consistent with mine.^12^ At that time I did not know Philippe very well. That deficiency was corrected a few years later when we shared a hotel room during a conference in Tenerife. Knowing that I, like he, appreciated good food and wine, Philippe had brought with him his copy of *Physiologie du Goût* by Brillat-Savarin (1825), which we greatly enjoyed discussing in the evenings.

IAU Colloquium 36 came on the heels of IAU Colloquium 38 on stellar convection, also in Nice, the two sandwiching the IAU General Assembly in Grenoble. It was during the former that our second son, Russell, was born. I missed the phone call from Cambridge, which was taken by Ed Spiegel. The first I heard was when Ed announced publicly what he considered to have been the most productive event of the meeting. I was absolutely delighted, of course, but sad that I had not been present at the birth, in contrast to being present for our other three children.

During that time in Nice my friendship with Jean-Paul Zahn, which had started in Woods Hole in 1968, blossomed. Jean-Paul had invited my family and me to spend a sabbatical year at the Nice Observatory, where he was Director, which, after the little aggravation that I now describe, I was able to accept. The original idea was to have stayed after the Nice IAU Colloquia in 1976. That was some seven years after my return to Cambridge, which exceeded the statutory minimum six that was necessary to qualify for sabbatical leave. However, my application for leave was refused on the ground that I hadn’t been in the university’s employ for long enough. On enquiring as to why simple arithmetic seemed not to work, I was told that the employment had to be continuous: I had been a member of the university’s staff, at IoTA, from 1969, but only until, but not including, midnight on 31 August 1972; then I was at IoA and DAMTP, but only from, yet not including, midnight on 31 August 1972. The university’s administrators had engineered my employment to be in open, not closed, intervals of time, a distinction that, I believe, is not explicit in the Statutes and Ordinances. It was to spite the memory of Fred Hoyle. I was furious. However, it is possible to mortgage future years of service, provided that at the time the applicant is not negotiating a post elsewhere and that the mortgage is supported by the relevant heads of department. Support from Donald Lynden-Bell at IoA would be assured, but I was doubtful about George Batchelor, who had not been on friendly terms with Fred. So I was surprised when he agreed without a moment’s hesitation. I asked him why. He answered that he was happy to do so because I would not be applying for leave again for at least fifteen years. “How can that be?”, I asked. “You’ll find out”, George replied with a smile. Of course he was right. Rosanne and I now had four children whose ages spanned more than a decade, and for many years to come there was always to be at least one of them at a critical phase of education. The sluggishness of the university administration to grant my leave delayed the visit to Nice by a year.

**Figure 7:**
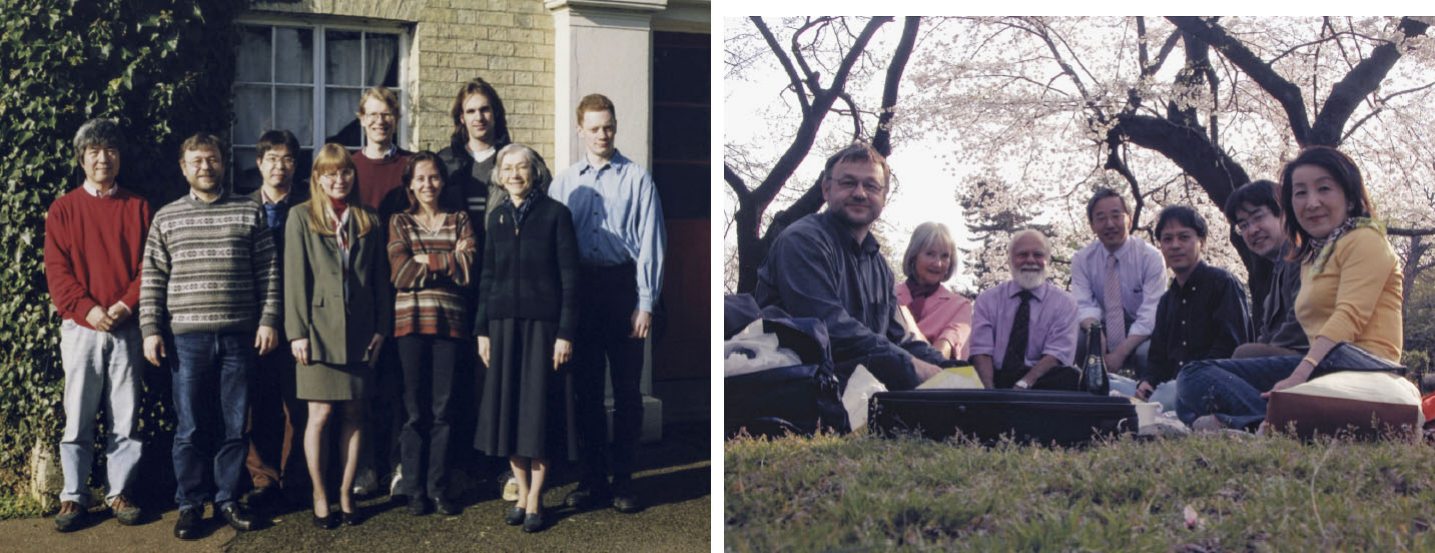
left: My research group at the end of the last century. Left to right: Umin Lee, Guenter Houdek, Masao Takata, Di Sword, Mike Montgomery, Pascale Garaud, Marcus Brügen, Eva Novotny and Chris Jordinson; right: Picnic with our Japanese friends: Günter Houdek, Rosanne, myself, Hiromoto Shibahashi, Takashi Sekii, Masao Takata and Minako Shibahashi.

To avoid suggesting further ill of my university, I hasten to refer to another occasion, one that was intended to be a term’s sabbatical leave in 2005, taken at the University of Tokyo. Within a month of arriving I became quite incapable of walking, as a result of a prior spinal injury; I spent a month in bed being treated as an out-patient at a local hospital, followed by two months as a resident, followed by some weeks in the University of Tokyo’s main hospital, which ended in seven hours of anterior surgery transplanting a piece of bone cut from my ilium to fuse two lumber vertebrae. I was given a single dose of morphine immediately afterwards; thence nothing. An apparently minor complication retarded my recovery, exacerbating the pain. Rosanne asked for permission to bring me a bottle of armagnac, in the hope that it would distract my attention, but was refused on the ground that the other three patients in my ward would want some. I offered to share it, of course, but the refusal was upheld, without further justification. Otherwise, from my perspective the treatment was faultless. However, the whole experience caused me to redefine what I perceive to be levels of pain, and I have found difficulty 13 years later with another serious illness, from which I am still recovering, to put my levels of pain on a scale of 1 – 10 in such a manner as to be understood by medical staff outside Japan. The reaction of the University of Cambridge to my Japanese experience was both surprising and heart-warming: without any request from me I was informed that my sabbatical leave had not been awarded for the purpose of spending time in hospital, that my time in Tokyo had been redefined as sick-leave, and that therefore I was still free to take a term’s leave. My subsequent interactions with my Japanese scientific friends, especially with Takashi Sekii, Hiromoto Shibahashi and Masao Takata (Figure 7), have all been both productive and enjoyable.

Back to Nice: Soon after I arrived I received by surface mail a request from Canada to instruct where I (D.O. Gough) wanted my belongings to be delivered. It transpired that Ian Gough (D.I. Gough), whose post at the University of Alberta I had declined more than a decade earlier, was to take sabbatical leave at Churchill College, where I am a Fellow. At the time there were no more than a hundred of us fellows, covering the whole range of academic disciplines, and we received only a handful of visitors per year. What an odd coincidence that one of them should have been Ian, and at the very time that I was to be away. The college porters, not yet aware of Ian’s impending arrival, had naturally forwarded the request to me. Of course I sent it straight back, but the diversion caused some delay in Ian’s delivery. About a decade later, Ian was awarded the Chapman Medal of the Royal Astronomical Society, and I received many letters of congratulations, including one, to my surprise, from the President of the Society!

Unfortunately, Jean-Paul and I spent precious little time working together: Jean-Paul was overwhelmed with administration. However, I was able to renew my acquaintance with Uriel Frisch, with whom I had many an interesting discussion, often on turbulence, and often also with Annique Pouquet, who worked in Uriel’s group. Mainly I worked with Gabrielle Berthomieu, Janine Provost, Arlette Rocca, and my student Alan Cooper who had accompanied me from Cambridge, together with Yoji Osaki who was also taking sabbatical leave at the observatory. We undertook a very thorough investigation of the reliability of the seismic calibration of the convection zone, investigating its sensitivity to everything we could think of. The outcome was an improved estimate, 200 Mm, of the depth of the convection zone. We were only half-way to completing a paper enumerating our tests at the time we dispersed; progress slowed and other events overtook us. There appeared to be little interest in the details amongst the scientific community, so the paper never appeared. However, we did at least present a summary of the results at a conference organised by Henry Hill and Wojtek Dziembowski in Tucson (Berthomieu et al., 1980).

To conclude, I mention two memorable incidents that occurred during our stay. The first illustrates a pleasant aspect of French attitudes. It concerns my French bank account. Because I was non-resident the account was external, and I was told by the bank manager in no uncertain terms that I could not pay cheques into it, nor allow it to be overdrawn. I had been appointed ‘Astronome Titulaire Associé des Observatoires de France’ and, because the French bureaucracy were predicted to take several months to start paying me, the observatory gave me a loan. To my surprise I received a salary cheque after only a couple of weeks, with a covering letter pointing out that a cheque had been adopted to obviate inordinate delay. I took it to the bank, where, notwithstanding the manager’s prior warning, it was readily accepted because it was drawn on a Government account. So I repaid the observatory’s loan. A week later I was summoned to the bank, on Friday evening, where the manager explained that he had been unable to deposit the cheque and that now my account was overdrawn, which was illegal. “What should I do?” I enquired. The manager, smiling, replied: “Nothing can be done before next Monday, so have a good weekend”.

The second incident was much more serious. We have four children, and Rosanne was to attend a part-time course at the university to improve her French, so we took with us an 18-year-old au-pair girl to help in the family. For her privacy I shall call her Susan. She was very beautiful with long fair hair, which made her stand out from the Mediterraneans. Moreover, she had led a sheltered life at home, and now felt liberated. On one of her evenings out she failed to return home. We were very worried, and phoned the police, who merely tried to convince us that this was quite normal for girls of her age. But we knew that Susan was different. The following night she did return: she had been kidnapped by an armed gang who operated along the coast between Nice and Marseille, and who intended to sell her for prostitution. Miraculously, Susan had succeeded in convincing them that she lived with an influential English family who would surely have them captured, but that if she were allowed to return home for just one night she could assure the family that she was leaving voluntarily, and there would be no repercussion. A rendevous was arranged for the following day. We were the only English family living in a small settlement in the hills overlooking Nice, Gairaut la Cascade, and were therefore immediately identifiable. So I drove the whole family up to the observatory for safety, where we could stay in the guest house. For her protection we decided to get Susan back to England immediately, and to obviate revealing her identity in Nice I bought her a ticket under the name of Jane Anderson to fly to Paris, where she would be met by Françoise Praderie, a trusted astronomer, who would give Susan a ticket for a flight to London, necessarily under her real name. The next day it was reported in the local newspaper that the kidnapping had been witnessed by an (unarmed) off-duty policeman who had no chance of intervening. So, after careful negotiation, Jean-Paul organised a meeting with the police, and Susan’s escape was postponed. It was the chief detective of the entire Côte d’Azure who took on the case, and with the help of information provided by Susan, the gang was captured a few days later and held in custody; we all returned to Gairaut. Later, after the detective had interviewed Susan again, he took us all to the airport in an unmarked car; we entered the airport through a small gate in the peripheral fence and drove straight onto the runway to a plane bound for England, with no explicit border control. The next day the incident was headlines in both French and English national newspapers, because Susan’s escapade had led to the capture of a long-sought dangerous gang. The gang were subsequently jailed for twenty years, so we were safe. To end the story, I needed a refund for Jane Anderson’s ticket, but was told by Air France that although they knew that the purchase had been made with my credit card, reimbursement could be made only to the ticket holder. I explained that Jane was no longer in France, and eventually persuaded the agent to agree to refund me if I produced a letter of authorisation from her. As I was leaving, I mischievously asked how they would know that the letter was actually from Jane Anderson. “Ah”, they said, “you are English; therefore we trust you”. Yes, I could be trusted, for I had learnt an important principle from Jean-Paul the Director: ‘Honesty in the large’. Susan has remained a life-long friend.

## Developing Inverse Theory, and Some Interactions with Observers

I was keen to be able to understand the implications of the much more extensive seismic data that were likely to become available. Merely calibrating theoretical models was too crude an instrument, and I started thinking about how to make inferences about the structure and kinematics of the Sun in a manner that did not depend directly on the complexity of assumptions that were required to manufacture theoretical models. The seismic oscillations are essentially adiabatic resonant acoustic-gravity waves whose physics is well understood: except in the turbulent near-surface layers of the Sun, propagation depends only on inertia (mass) density, and the pressure providing the restoring force, together with the relation between (adiabatic) perturbations of the two. The pressure gradient and density are related hydrostatically via the Poisson equation determining the gravitational potential. Thus, given an equation of state, only pressure and density, or any function solely of them, are seismically accessible. It is clear that there is no redundancy. Therefore, no intrinsic aspect of the system, such as the veracity of the equation of state, could possibly be established with seismic observations alone. Moreover, the physics of acoustics is very simple, so if an investigation were to be carried out with due diligence, the conclusions must be robust.

The situation that was about to be faced is very similar to that encountered by geoseismologists, except that it is less complicated, at least in principle, because the Sun is fluid everywhere. I sought advice in Cambridge’s Department of Geodesy and Geophysics, as it was then called, just next door to the IoA. Most helpful was a friendly student, Kathy Whaler, now Professor of Geophysics at the University of Edinburgh, who patiently taught me the rudiments of inverse theory. She introduced me to papers by Backus and Gilbert (1967, 1970), Franklin (1970) and Jackson (1972), also Wiggins (1972), Gilbert and Dziewonski (1975) and Bob Parker (1977a,b), whom I had known when we were both students in Cambridge, and Sabatier (1977a,b). Interestingly, I had already heard a lecture by Freeman Gilbert when I was a Fellow at the GFD programme at WHOI in 1965, which I had found interesting conceptually but had not retained any of the details. The principle was to set up parametrised functional representations of the seismic variables defining the background state, typically as expansions of kernels (Fréchet derivatives, sometimes called data kernels) in integral constraints that represent the observations;^13^ and then to minimise the difference in some suitable function space between theory and observation subject to the requirement that no demand be made to force agreement to be closer than is dictated by observational error, for otherwise spurious structure would be introduced. The outcome is called spectral expansion. The procedures to determine it were originally designed for linear constraints; nonlinearity can be accommodated iteratively via successive linearisation. The process of limiting spurious structure produced by data errors is called regularisation. As a quite separate matter, Backus and Gilbert (1968) had made a study of the resolving power of gross Earth data, represented as the maximum degree of localisation that can be achieved by linearly combining the data kernels, again subject to appropriate regularisation. In my opinion, these maximally, or optimally, localised averages (abbreviated by Jørgen Christensen-Dalsgaard and Michael Thompson to OLA) are obviously the preferable way to present an inversion of the data, for they are relatively simple to comprehend, at least where they are well localised. Where they are not, interpretation is more difficult, because the data provide only scant information about the behaviour sought. Interpreting a spectral expansion in such circumstances is even more difficult, because its relation to the functions sought is usually considerably less local: each point-wise function determined by a linearised iteration is actually an average of the seismic variable it represents, usually with substantial distant sidelobes. Kathy is open minded, and readily accepted my opinions, despite having to tell me that geoseismologists rejected OLA as a means of representing the data because of a misleading tendency to view the averages as point-wise values, even though they may not actually satisfy the imposed constraints. But why think of them as point-wise values? And why should one prefer just one of the (infinitely) many functions that happen to satisfy the data within their uncertainties? Some decades later I learned that geoseismologists have now ‘discovered’ that OLA is a useful representation of inversion, although they do not refer to it as such.

I undertook to invert some artificial seismic data of the kind anticipated from observation, first computing OLA of sub-photospheric flow from rotational splitting of high-degree p modes (Gough, 1978a). That demonstrated that accurate inferences were extremely likely to be accessible. A second inversion, computed with the help of my student Alan Cooper, provided the density distribution throughout a solar model inferred from low-degree mode frequencies, obtained by linearising the difference between the putative Sun and a reference model; we adopted both OLA and spectral expansion, in order to compare the two. The outcome was not published until several years later (Gough, 1984b). It illustrated an important conclusion that I had announced earlier at a CNRS conference (Gough, 1978b) simply from inspecting data kernels, namely that a reliable inference of conditions in the Sun’s core, where the nuclear reactions are taking place, really requires g modes.

I was still inexperienced at publishing articles for conference proceedings. Whilst at Nice I received a letter from the editorial office of CNRS telling me that all the contributions bar mine for the 1978 conference had been received, and that I was holding up publication. So I quickly finished the article and sent it off without even waiting a night to cogitate. The next day I found a dreadful error, fortunately typographically trivial (computer typesetting was far in the future). I phoned the editorial office in Paris, and arranged for the correction to be made. Then I casually asked, since I was feeling so guilty, when publication was likely to occur. “Quite soon, we hope”, came the reply, “We have nearly half the papers now”. Many years later I was again being harassed for being late, this time by Hiromoto Shibahashi, who also told me that I was holding up publication because I was the last. I duly sent in my contribution, asking Hiromoto whether mine really was the last contribution that he had received. “Yes”, he replied immediately, “and now I shall write to all those who have not yet submitted to ask them to hurry up”. I have a reputation for being late for sending in articles, yet, so far as I know, I have never actually been the last.^14^ I also have the (partially valid) reputation for not replying to e-mails.

After returning from Nice I experimented with an alternative inverse approach based on asymptotic theory, either by approximating exact integrals for the (linearised) mode frequencies (Gough, 1984c), which I called Duvall’s law (Duvall Jr, 1982), or deriving resonance conditions from ray theory (Gough, 1986). I realised that the formula can be transformed into Abel’s integral equation for the quantity $w=\omega/L$ in terms of $a=c/r$ (where $\omega$ is the frequency of a mode of degree $l$ and $L=\sqrt{(}l(l+1))$ or $l+1/2$, and where $c(r)$ is the sound speed at radius $r$), which I recognised from the work of Brandt (1960) on galactic structure to be invertible to yield $a$ as a function of $w$; I was not yet aware of an analogous geoseismological analysis by Brodskiı̌ and Levshin (1979). Although the method is approximate, it has the advantage of having retained non-linear terms in the structure of the background state and of not being dependent on a reference solar model. It is also quick and easy to apply. Moreover, it provides analytical formulae that enable one to appreciate the manner in which different aspects of the star influence the oscillation frequencies. This led me do design signatures of solar properties from appropriate combinations of frequencies that can be deployed directly for precise model calibrations. Detractors have protested that asymptotic methods are too inaccurate to be useful, which can be true if they are used inappropriately; also, that any of the signatures so found could equally have been obtained by studying more precise numerically computed eigenmodes; that too may be true, but the reality is that most, if not all, of the signatures that have been used to calibrate specific properties of solar models have actually been derived from asymptotic analysis. Moreover, because analytical approximations are usually so very simple to use, there have been times when they have been more reliable than ‘realistic’ numerical computations (e.g. Gough, 1992). Indeed, because of my interest in convection, I have used asymptotics to set a 5% upper bound on $\nabla-\nabla_{\mathrm{ad}}$ in the body of the convection zone where mixing-length theorists take it for granted that it must be tiny (Gough, 1984c).

By now, others were starting to invert helioseismic data, and I was invited to review the procedures in the 100th volume of *Solar Physics* (Gough, 1985). In 1987 I was permitted a post-doctoral research assistant, which seeded funding for extension into a wonderful group which peeked in the late 1990s (Figure 7).

As an aside, I mention my biggest disaster with asymptotics. I had visited Wojtek Dziembowski in 1981 at the Copernicus Institute, where we derived a sequence of second-order differential equations for high-order acoustic stellar oscillations that are amenable to asymptotic analysis and that incorporate the perturbation to the gravitational potential. Post-Cowling approximations, we called them. Somewhat later, in 1991, I was invited to give NASA’s Wernher von Braun Lecture in Huntsville. Rosanne and I went by way of Miami, to take the opportunity to visit the Everglades before they disappear, and there my briefcase was stolen. It contained the post-Cowling derivations. I have never succeeded in reproducing them. I wrote to Wojtek for copies of my notes, but all he had were the final expressions that he had used for computing the eigenfrequencies, which were not in an easily interpretable form. I had recorded the first post-Cowling approximation in my lectures on stellar oscillations in the 1987 Les Houches summer school (Gough, 1993), but the better, second, approximation eludes me to this day. I began to suspect that I had inadvertently made a mistake originally, but the numerical solutions to Wojtek’s version of the equations are more accurate than anything I have been able to derive since, which makes me wonder.

An important quantity needing to be ascertained is the Sun’s helium abundance, $Y$. Already there were indications of a relatively high value from the depth of the convection zone that had been inferred from high-degree p modes (Gough, 1982), but that relied upon the near-surface structure of just the outer layers of theoretical solar models. Greater, global reliability required supplementation with modes of low degree that penetrate to the core. Jørgen and I had already persuaded a student at IoA, Guy Morgan, to compute a sequence of solar models having different initial values $Y_{0}$ of $Y$ (and therefore different initial values $Z_{0}$ of the heavy-element abundance $Z$) and that were presumed to have been dirtied by in-falling metal-rich material during main-sequence evolution such as to yield the present-day photospheric abundance. We computed the frequencies of low-degree modes in anticipation of future observations (Christensen-Dalsgaard, Gough, and Morgan, 1979), and were therefore delighted when, at a conference in Tucson organised by Henry Hill and Wojtek Dziembowski in 1979, George Isaak announced that his team had measured a spectrum of frequencies in their whole-disc observations (Claverie et al., 1980), with mean separation 67.8 μHz. Jørgen and I were able immediately to infer that what had been observed were groups of modes of alternately odd and even degree, each having mean separation 135.6 μHz, and that $Z$ appeared to be just a little lower than 0.02, the most popular value of the time, and $Y$ a little lower than 0.25.

At that time, Eric Fossat and Gérard Grec, with whom I had become friends during my sabbatical year in Nice, were planning with Martin Pomerantz to go to the South Pole where they could gain longer continuous whole-disc observations of the Sun. On their return they showed me their stunning preliminary results, which resolved the separate contributions from modes of differing degree. I wanted their observations to get high visibility, and suggested publication in *Nature*. Eric was reluctant. So I tempted him with the possibility of the front cover; afterwards I phoned Philip Campbell, the Physical Sciences editor at the time, telling him that he might secure the important paper if the front cover were offered. The deed was done. Now, individual frequencies were available (Grec, Fossat, and Pomerantz, 1980), and a more detailed analysis was possible. Jørgen and I found that we could not reproduce all the observations by interpolating between our dirty models, and concluded that better account of the atmosphere was required; our efforts revealed that the best-fitting model was less helium deficient than we had first suspected in Tucson. A subsequent calibration by crude least-squares fitting of raw frequencies – more sophisticated signatures of the kind to which I alluded earlier had not yet been adopted – strengthened the conclusion that $Y \simeq0.25$ (Christensen-Dalsgaard and Gough, 1981), especially when the high-degree calibration was also taken into account. However, a model with low helium abundance fitted the low-degree (but not the high-degree) data almost as well.

In November 1981 Henry Hill visited me in Cambridge to show me his latest data on the oscillations of the Sun’s periphery. He produced a spectrum on a scroll of paper about 30 m long that we unrolled along the corridor of the IoA, revealing several prominent peaks in the g-mode region. The resolution was fantastic, and we were able to measure what appeared to be rotational splitting frequencies of grave low-degree multiplets. The non-uniformity of the spacing agreed precisely with what one would expect from a solar angular velocity that varied only weakly with position. I recall measuring the frequency separations one-by-one with Henry, and being astounded, as each one agreed perfectly with the values I had computed from theory. I was convinced. Somewhat to my surprise, however, Henry justified the authenticity of his data by telling me that he had no access to a theorist, so he couldn’t possibly have invented them; I would never have dreamt otherwise. Henry and I then decided to work on bounding the Sun’s internal angular velocity $\Omega$ in order to set constraints on the oblateness of the Sun’s gravitational potential, a subject that Henry had already been investigating from direct measurements of the shape of the photosphere. We agreed to publish the outcome together in *Nature*. However, when we came to try to write the paper we could not agree on how to present our results. I had concluded that the data were not inconsistent with General Relativity, whereas Henry, who confined his thinking to a much more limited range of possible variations of $\Omega$, believed that with 95% confidence they contradicted it. We would have to explain carefully the evidence for and against, but Henry was intransigent; a compromise was out of the question, so we had to publish separately, I alone (Gough, 1982), and Henry with his student Randy Bos, together with his postdoc Phil Goode who all along had been carrying out oscillation calculations for them (Hill, Bos, and Goode, 1982). The following summer I was travelling to the US on a Continental Airlines flight when I was surprised to see on the cover of the inflight magazine a picture of Einstein crying. Inside was a highly inaccurate article that had been instigated by Henry, as I learnt later from Irwin Shapiro, in which Henry complained about scientists from universities more prestigious than his, namely Bob Dicke from Princeton and me from Cambridge, being taken more seriously than he: Henry had previously distanced himself also from Bob over the direct oblateness measurements. Not only this inflight article, but also one similar that was published in the Chicago Tribune (Weingarten, 1982), revealed how hurt poor Henry was. Yet that was only because Henry himself was not prepared even to contemplate possibilities beyond his own prejudices. And, unfortunately, he engendered similar distancing from other colleagues. Happily, after a cooling-off period, our relationship was restored, although not to the same degree as before.

I sometimes wonder whether there was an underlying difference between the dispute with Henry and the disagreements I have had with others. The latter have usually ended with a wager, to ensure no personal hard feeling, the stake being some form of alcohol, usually an imperial pint of English beer. Henry, however, was teetotal, so no such wager was possible. Besides, he regarded the dissipation of potential tension by such means as a trivialisation, which certainly it is not. On occasion I wager against greater stakes, such as two bottles of pre-1970 Grand-Cru Bordeaux, which I lost to George Isaac and paid up at a college feast to which I invited him especially for the hand-over: in the 1990s George had announced that he would soon be able to measure oscillatory solar velocities as low as 1 mm ${\mathrm{s}}^{-1}$, so I (having estimated g-mode amplitudes of a comparable magnitude) had perhaps foolishly wagered against his subsequent claim that g modes would not be discovered by the end of the century. Other such wagers are pending, the most serious being with Jack Harvey. That wager, made more than three decades ago, is based on a principle similar to that with George: I bet that a particular one of Jack’s tentative observations would turn out to be correct, he that it would not. The stake is determined operationally and depends on the circumstances under which the wager is decided. Moreover, it is potentially susceptible to influence by others, and therefore all the details are a tight secret, kept in a sealed envelope in the custody of a mutually trusted friend. The cost is almost certainly an increasing function of time, so both Jack and I are becoming quite nervous.

When Henry was visiting me that November I was able to calculate rotational splitting frequencies of a solar model quite quickly because I had already carried out a similar investigation arising from the assumption that temporal variations in Dicke’s and Goldenberg’s oblateness measures (Dicke, 1976) had arisen from dissipation of seismic oscillations in the atmosphere (Gough, 1981a). I had corresponded with Bob during the writing of that paper, which engendered an invitation to Princeton, where we spent an entire week in his office discussing physics. Sometimes, one or both of his students, Jeff Kuhn and Ken Libbrecht, joined us. It was a most exhilarating experience. I had been warned beforehand that Bob was an acerbic character, but in my experience he was quite the opposite. To be sure, he would present his cases forcefully, but he enjoyed a forceful rejoinder. He always responded logically and cheerfully to any counter-attack, and was amenable to both listening to an opposing view and being open to persuasion by it. We had a wonderful time.

## Boulder: Detecting Horizontal Flow

By the end of 1973 Jüri was well established at JILA. He invited me to visit Boulder, during which time we could start writing up the work on modal convection that we had carried out with Ed Spiegel in New York. The visit was too short to complete even one article, and it took several more visits before publication ensued. The visits became a regular event, thanks to Jüri’s kind financial assistance, and we were able not only to complete the writing, but also to carry out a modal analysis of ‘doubly diffusive’ convection, which I discuss in Section 23.

By the time that task was complete, I was already almost fully occupied with helioseismology, and not unnaturally I discussed it with Jüri. By now I was visiting Boulder with Rosanne and our children for what was becoming a usual month in the summers. Jüri was deep into direct numerical simulations of convection, and we resolved to consider how that work could be seismically tested. We were not expecting imminent observations of oscillations with wavelengths much shorter than the dominant convective scale, so we wanted to study advective frequency shifts of seismic waves whose horizontal scales are comparable with the convective scales. We weren’t in a position to address the dynamics properly, because to calculate the response of the convection to the seismic waves, which my experience with classical pulsators had suggested might be necessary, requires a tractable theory of convection, which we didn’t have. So, we replaced non-linear convective modes with linearised wave modes; even that was quite complicated. There was a very large blackboard in a lecture room near by on which I could carry out the calculation, in continual discussion with Jüri who assiduously caught my many mistakes. We had hoped to present the results at an upcoming conference in the Crimea that Andrei Severny was organising. Unfortunately, progress was slower than we had anticipated – that seems to be the story of my life – so at the very last moment we hastily carried out a simple calculation in the high-wavenumber limit, and were able at least to lay down the principles of seismic inference of macroscopic flow (Gough and Toomre, 1983). The problem is linear, unlike structure inversions, and is therefore much easier to carry out. The following year, together with Jüri’s student Frank Hill, we applied the technique to solar data to estimate radial variations of subsurface velocity, detecting evidence of temporal variations that we tentatively suggested might be giant cells (Hill, Gough, and Toomre, 1984).

The high-wavenumber limit is not appropriate for determining all aspects of horizontal background flow ${\mathbf{U}}({\mathbf{x}},t)$, and one must be concerned about the effect of shear on the seismic spectrum, and the fact that the flow varies with time. Perhaps the simplest approach is to analyse waves over discrete patches of the Sun’s surface that are sufficiently small for the shear to be negligible and over sufficiently short times for the temporal variation also to be negligible, and then piece the results together. The signal to use is then simply a Doppler shift associated with the average $\overline{\mathbf{U}}$ over the patch. It can be estimated from constant-frequency sections of the three-dimensional power spectrum, whose power to a first approximation is concentrated in rings displaced by an amount proportional to $-{\mathbf{k}}\cdot\overline{\mathbf{U}}$, about which I had lectured at a summer school in Les Houches. I discussed it with Frank, who took up the idea and was the first to use it on real data (Hill, 1988). An obvious drawback of the procedure is that over small areas one cannot determine wavenumbers ${\mathbf{k}}$ accurately. It is surely better to use larger areas, but then one must be able to cope directly with the variation of $\mathbf{U}$. I first studied the possibility of measuring the variation of the phase of the wave, and together with Keith Julien and Jüri showed with artificial data that by regarding a packet of collinear waves as a single entity one could invert the phase for ${\mathbf{U}}$, at least when ${\mathbf{U}}$ is stationary, removing interference effects by using the fact that they travel with the (non-zero) group velocity (Julien, Gough, and Toomre, 1995). Then, Bill Merryfield, Jüri and I attempted to generalise the procedure to two horizontal dimensions (Gough, Merryfield, and Toomre, 1998); here we came badly unstuck, because the group velocity of a unisonous packet of almost collinear acoustic waves is zero, and therefore interference cannot be distinguished from variation of the background state. Jüri, quite sensibly, had taken a pragmatic approach, and invested a great deal of effort in bulk ring displacements (called ring-diagram analysis by Frank) in small patches, principally with his research assistant Deborah Haber, and made much headway in mapping large-scale convective flows (e.g. Haber et al., 1999). It has now become a standard procedure. I was still concerned about accuracy, and had foreseen that larger patches, though more expensive to analyse, should be the way forward. Then, with Brad Hindman, who was then another of Jüri’s assistants, and Michael Thompson, one of my former students, Jüri and I investigated power spectra of two-dimensional ensembles of waves propagating across a large-scale shearing flow (Hindman et al., 2005), confirming my suspicion that to a first approximation the outcome is simply a Doppler shift of the dispersion relation by an amount corresponding to the uniformly weighted average velocity $\overline{\mathbf{U}}$ over the patch. So, the time was ripe for larger patches to be treated at least in the manner of the small patches, except that now they should overlap in order to permit a horizontal inversion to determine from $\overline{\mathbf{U}}$ the variation of ${\mathbf{U}}$ over scales much smaller than the size of the patches. Not only should the result be more accurate than using small patches, but by being able to access lower wavenumbers the outcome would permit deeper penetration below the surface of the Sun. I wrote out an inversion procedure and carried out an error analysis, and Jüri organised his student Ben Greer, supervised by Brad, to do the computations. The intention was for us all to write a joint paper, but that hasn’t happened.

Jüri had embraced helioseismology, and he made enormous contributions through training students, and encouraging major projects such as GONG and MDI on SoHO. He made it possible for me to visit JILA almost every summer for more than 35 years, which was extremely rewarding, both intellectually and socially. In 1986 I was made a Fellow Adjoint of JILA, which was largely the result of Jüri’s influence.

## Hurried Investigations

I have already mentioned various hurried investigations: the pulsating mixing-length theory at Woods Hole, the expansion in Boulder of high-wavenumber acoustic waves perturbed by horizontal flow, the solar spoon in Cambridge; and perhaps I should include the solar-diameter measurement in Indonesia that I describe in Section 18, and that, having taken well under 48 hours from final submission to being publicly available, was without doubt my fastest publication. What these investigations had in common is that they were each carried out in the face of a deadline determined by external circumstance. Here, I add to the list one against a deadline that was self-imposed: Ed Spiegel was visiting Cambridge, and at coffee time one Monday morning he remarked to Jim Pringle and me that the publishing of much of the work on ‘hot topics’ carried out at the Institute of Astronomy, and in other ‘high-profile’ astronomical institutions too, was a race against perceived competitors, and that the kudos gained from being the first engendered an air of superiority. Was that fair? Could we join the club, even though our interests were hardly ‘hot’ and we had no desire to race others to obvious goals? We decided to put it to the test: to invent a research project, complete it, and submit by Friday’s post a paper that would be accepted for publication in ${\mathit{Nature}}$. That gave us four-and-a-half days. We decided on likening solar supergranulation to seiches in lakes, and promptly repaired to a local village hostelry to spend the rest of the day studying the sloshing of beer in a glass. We spent the next day playing with orders of magnitude, in keeping with the activities of our non-mathematical colleagues, and on Wednesday we carried out experiments trying to confine seiches with wire structures meant to represent fibril magnetic fields. That didn’t work, so we rejected the analogy – here we were forced to adopt a form of integrity appropriate to my chemistry scholarship examination – and on Thursday we drafted a paper. We revised it on Friday morning and put it in the post to *Nature* by 4 pm. It was accepted without modification (Gough, Pringle, and Spiegel, 1976a).

In contrast, I mention also my most protracted publication. It was written with Takashi Sekii (Figure 12), demonstrating the kind of trap into which the unwary might fall by not taking due account of correlations between data errors in inverse analyses. The referee for *Monthly Notices* was very slow, and by the time his report had arrived both of us were occupied with other matters. Consequently, we were even slower. The first report demanded addressing a hardly pertinent circumstance, to which we responded with a paragraph written entirely in the conditional mood, thereby deliberately rendering it hard to follow, as it should have been. In his report on our revised version the referee commented that it was fortunate that he hadn’t yet deceased. The paper finally appeared almost seven years after the original submission (Gough and Sekii, 2002). Roger Tayler, the Editor-in-Chief, told me that our delay was the longest the journal had ever experienced. Now, the journal imposes a maximum author response time, so unless that policy is changed Takashi and I will hold the dubious record for ever.

## On Becoming an Observer

Having spent a great deal of time assiduously observing Tom Duvall’s and Jack Harvey’s data, I considered myself an observer. “Oh no you’re not”, retorted Jack, “You haven’t got your hands dirty”. So I told him the following story:

It was a cloudless afternoon in Cambridge when I received a telephone call from Jack Miller,^15^ a friend in the Department of Geodesy and Geophysics. A bright object had been spotted in the sky, and John had been contacted by the local newspaper enquiring what it was. Was it a UFO? At that moment, obviously yes. John was at home, on the other side of Cambridge. I went outside and saw the object immediately. Then I sought an observer,^16^ but none was to be found; the weather was too good to be in the office. I was able to find the key to one of the telescopes, and by now had attracted a growing group of student theorists, all as capable as I of using a telescope. We looked at the object, which quite clearly was a stratospheric balloon. How big was it, and how high was it flying? The telescope had no angular scale, so we went back outside, and I held up my thumb to estimate the balloon’s angular diameter. John gave me his view of its position relative to the Sun, and also the location of his home. I too estimated the balloon’s position relative to the Sun as seen from the Institute of Astronomy (which was confirmed by the students), and from which I could calculate the height of the balloon by triangulation. The answer was about 10 km beneath the ground behind me! I am sufficiently astute to have realised that that result could not have been correct. The students checked my calculation and could find no error. “How big is a balloon?”, we asked ourselves. Nobody knew. So I guessed a diameter of 50 m, and from my estimate of the angular size and the inclination we declared the balloon to be 15 km above the ground. I gave the information to the local newspaper, emphasising the uncertainty. However, what was published the next day omitted the caveats. Surely my preference for my second inference showed that I was more of an observer than a mathematician. But Jack would have nothing of it. My hands weren’t dirty enough.

As a sequel to this story, the newspaper subsequently tracked down the origin of the balloon. It had been launched from Bristol by Peter Fowler to measure cosmic rays: when over Cambridge the balloon was actually 100 m in diameter, and its height was 30 km. So at least my angular ‘measurement’ was fine. I had another success years later when I was invited to be a ‘resident astronomer’ on a Caribbean cruise to see Halley’s comet. I was quite unfamiliar with the southern sky where the comet was located at the time, and although I had tried to learn the pertinent constellations I was horrified to observe that for nearly a week the sky was almost completely overcast. In fear of being lynched by the passengers I went to the bridge to learn the course the ship was sailing, and calculated the direction in which my on-board telescope should point. Lo and behold, when the comet appeared through a gap in the clouds it passed periodically through the cross-wires as the ship rolled, and the passengers were satisfied.

In June 1983 I received another pertinent telephone call, this time from John Parkinson^17^ at University College London, asking me if I would go to Indonesia in five days’ time to help measure the diameter of the Sun. I was to take the place of Richard Stevenson, who had contracted glandular fever. Teaching was over, and thanks to my dear wife Rosanne, who was to forego being taken to a good restaurant in celebration of an anniversary of the day we met, I was able to oblige. The objective was to determine the location of the southern edge of the shadow of the Moon during a total eclipse of the Sun; John would locate the northern edge. By so doing the uncertainty in the direction of the light from the Sun due to refraction by the Earth’s atmosphere is reduced by a factor of about 400. The method had first been carried out by William Herschel; John wanted to find out whether the Sun had changed size since then, and had persuaded the popular journal *New Scientist* to sponsor the endeavour. Months before the eclipse, John had organised some twenty high-school students to be at my disposal; I was to place them along a 2 km north–south line between two villages not far from Surabaya and have them observe the eclipse to determine which of them experienced the full shadow and which did not. My trip was to last a week, Sunday to Sunday, because Garuda Indonesia Airlines were offering a substantial fare reduction for travelling on those days. The eclipse was to occur on Saturday, so I would have nearly a week for vacation of my choice provided that I guarantee to get to Surabaya by Friday.

Actuality turned out to be different. At Jakarta Airport I was met by a frantic John who insisted that I take the next flight directly to Surabaya; a problem had arisen because the government had decreed that no Indonesian could watch the eclipse, for fear of being blinded: their only choices were either to watch it on television or go to the mosque to pray for the dragon to spit out the Sun. The Mayor of Surabaya, who was also the Minister for Tourism, was enforcing the law. My task was to plead a special case. From the manner in which John informed me of the predicament I wondered whether he had known this all along. Thus, my experience of Indonesia was not to be the vacation that I had expected: almost the entire week was spent in an intransigent mayor’s office.

As I was the only westerner in my hotel, my presence aroused interest. Word of my negotiations got about, and at dinner on Thursday evening I was approached by a very pleasant young lady offering help. She was a journalist from Malaysia. She suggested accompanying me the next morning with her photographer, ostensibly to interview the mayor for a feature on tourism in Indonesia that she pretended to be writing, and then wait until my business was over so that we could share a taxi home. She pretended to speak no Indonesian, which wasn’t the case, so her interview was conducted in English. Next came my marathon, copiously photographed, which unbalanced the mayor. Eventually, the mayor did concede that his staff might help, but that they needed a substantial payment, which should (of course) be channelled through him. Occasionally his secretary would come in to give him messages, in Indonesian of course, and call him away temporarily. Whilst out of earshot, my companion could report that the mayor was being phoned by the Prime Minister who condoned his trying to extract from me as much money as he was able, but to ensure that in the end I would be allowed to carry out the observation. That cemented my tactics. Came the evening when all the staff were about to go home, the mayor grudgingly gave me permission to try to persuade some of them to help; as I was introduced to the shop steward, Andrini Daniharkati, dozens were queuing behind her hoping to be chosen!

I had already hired a bus, bought crates of refreshment, and had with me a stack of *New Scientist* T-shirts to distribute as mementoes. Off we went the next day to a small village near Bangil, some 90-minutes drive from Surabaya. I paced out a line in the heat of the noon-day Sun, only a few degrees from the equator, and distributed my team along it, providing them with suitably fogged film and teaching them how to use it. I told them what they should expect to see, and instructed them very carefully on what they were to report, for, to keep the peace, I had to lead them all to believe that they would witness a total eclipse. And so, fortunately, they believed that they had. Afterwards they were not to move until I returned to interview them. I was at the northern end of the line, just over a hillock, out of sight of everyone. An old man appeared from a primitive hut, the head of a village near by, who, it transpired, had a degree from Cambridge, and then came a small boy about three years old. I gave them both some fogged film, and when I went to show the boy what to do he immediately demonstrated that he knew; he had been following me and watching me instructing the others. Then came the eclipse; it was my first. It was wonderful. Even better in all respects than I had expected. Just the three of us, peacefully alone with barely perceptible chanting from a distant mosque praying for the dragon to spit out the Sun. One thing that I hadn’t expected was that as it got dark the mosquitos started biting.

**Figure 8:**
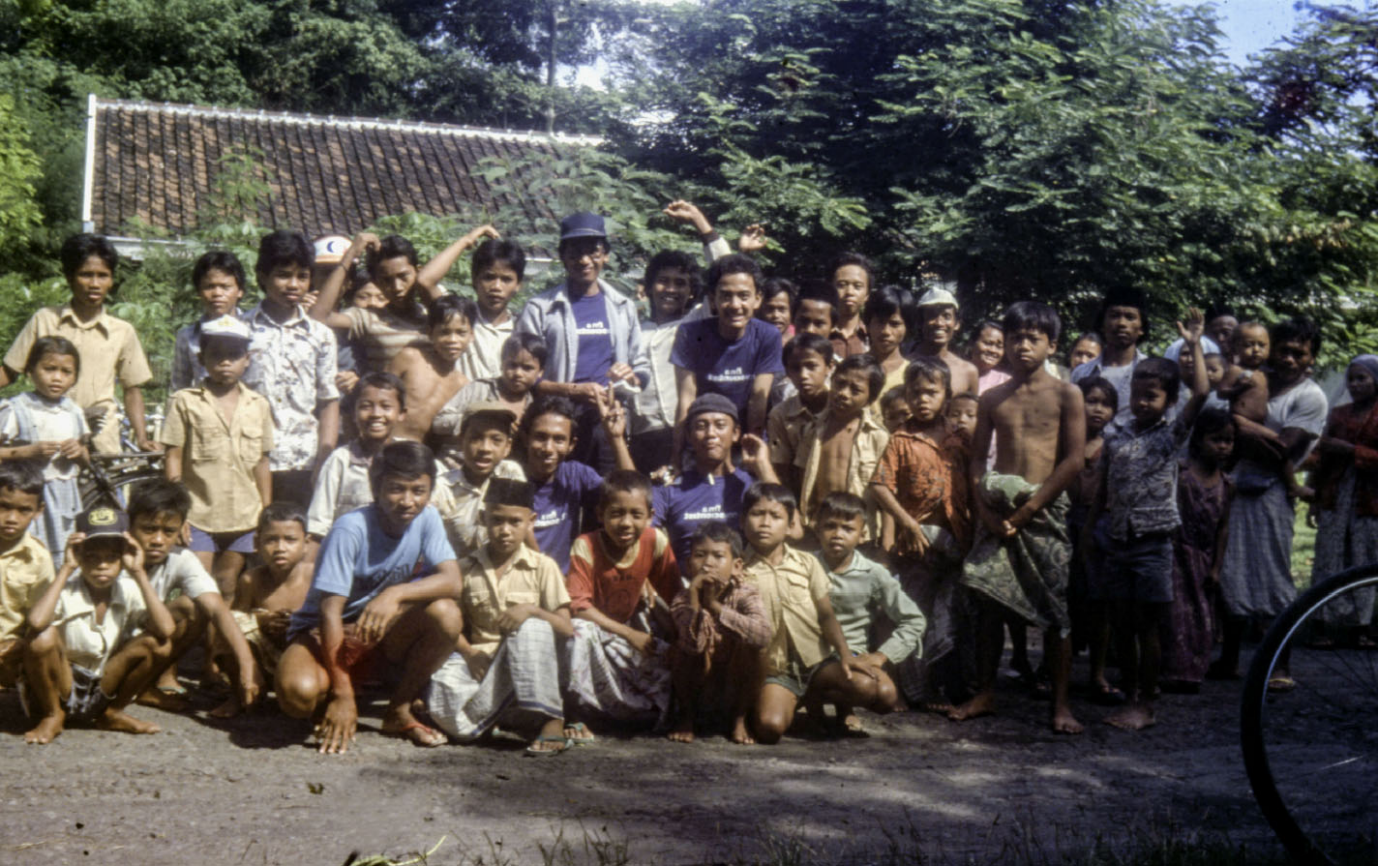
My attempt of a photograph of the three faux Bangil warriors who feigned to attack me, here obscured by the local villagers who were quick to ensure that they were also, and exclusively it seems, in the picture.

After the eclipse I was entertained by the Headman to tea, and about an hour later started my walk south. The reports were superb, and made it quite clear where the edge of the shadow had been. The road ended at another village, more accurately a hamlet, beyond which was a field and then forest. As I was talking to the last member of my team, surrounded by expectant onlookers who seemed to have appeared out of nowhere, I was distracted by three wild Madurese, dressed only in loincloths and warpaint, carrying tall shields and brandishing spears, running towards me out of the forest, and shouting loudly. I was probably terrified. I had no time to consider my position, but I suppose I must have reasoned that either they were seriously belligerent or they were not, and if the former there was no escape. So I dropped my notebook and ran towards them, waving my arms and shouting. They turned heel and fled. The villagers were delighted that I had prevailed. The ‘warriors’ later returned, laughing, and I waved my camera as a request to take their photograph. They were delighted. But so too were the onlookers. Before I could angle my camera, the entire village had assembled around my subjects who were now lost from view (Figure 8).

The next day, Sunday, John and I met in Jakarta and mapped out our report for publication; John and the *New Scientist* writers had already prepared the background material before the trip. John was not returning immediately to London. I wrote up the article (Gough and Parkinson, 1983) on the overnight flight, and delivered it to the *New Scientist* offices by noon on Monday, together with photographs that I had taken. The issue was printed and delivered to newsagents throughout the country by Wednesday morning.

I hadn’t realised before that observers can publish so quickly in other than IAU telegrams. Nor had I been aware of the tribulations and dangers that they face in order to observe. Nevertheless, I enjoyed the experience enormously. And I was rewarded: the next time I met Jack Harvey he welcomed me into his club.

## A Paper Unpublished

There have been many, but I select this one because it is typical of the wonderfully relaxed Mediterranean way of life. I was in Eric Fossat’s office in Nice and spied a long scroll of paper that turned out to hold a highly resolved spectrum of the South Pole observations, extending to the Nyquist frequency. I saw an obvious high-frequency peak that neither Eric nor Gérard had noticed. So I measured it and learned that it corresponded to a period of 160 seconds. How interesting! That was surely material for *Nature*, who had published the 160-minute observations. I surmised that the low-degree modes averaged out the relatively small-scale turbulent fluctuations in the outer layers of the Sun, and were resonating as a chromospheric mode. That would provide an important atmospheric lengthscale. So Eric, Gérard and I repaired to the patio of a pleasant cafe overlooking the sea, and with some good wine we wrote a letter to *Nature*. How civilised!

Having been bitten in the past, Eric wanted to check the frequencies, and frequency combinations, of all the moving parts in his instrument, before we submitted the letter. I went back to Cambridge and polished the text. Then, I received a message from Eric saying that no damning instrumental frequency had been found, yet insisting that nevertheless he wanted his name to be removed from the letter. We could hardly proceed without him. However, our day had not been wasted, because we had enjoyed an extremely pleasant day by the sea discussing physics. Moreover, Eric’s wisdom was later confirmed by the absence of our frequency from all subsequent observations.

## An Interesting Conference

I have attended many interesting conferences. Here I pick one, not entirely at random. It was a meeting on solar oscillations held in Catania in 1983. The invited speakers enjoyed wonderful Sicilian hospitality, which provided the luxury accommodation of a spa in Acitrezza, a village outside Catania overlooking the rock said to be the home of Cyclopes. As a result we missed the excitement of a bomb that exploded in the foyer of the hotel in which the other conference participants were housed. The bonus for us, however, was that we were instructed merely to deposit the keys to our apartments at the gate house on departure. An interesting morsel of knowledge I acquired was that because Sicily had been invaded over the millennia by so many aggressors who usually established oppressive governments, it was necessary to support an unofficial organisation to protect the common people from them.

Our day off was a trip to Siracusa, where we saw remains of ancient Roman architecture often built on foundations of older Greek buildings in considerably better condition. Our coach was escorted by six armed police on motorcycles. While they were waiting in the car park before our departure I walked over to them to ask why they were there, but received no more than a scowl. Our host, Lucio Paternò, knew nothing about them. I thought that perhaps they were to protect us. However, Lucio learned several days later that they were for protecting the Sicilians from us! It was known that Wojtek Dziembowski, from Poland, then on the other side of the Iron Curtain, was among us, and they feared that we might be a new aggressor. However, they did turn out to be useful, because on the way home we encountered a 5-kilometre stationary traffic jam, held up by rock blasting for widening the road. The police halted the blasting and then escorted us past the jam on the wrong side of the road, enabling us, and no doubt more pertinently them, to get home in time for dinner.

In Siracusa we visited the beach where Archimedes is said to have studied geometry by drawing in the sand. The myth of his burning Roman ships with sunlight focused by concave mirrors is obviously faulty; more plausible is that he frightened them with the glare, possibly even blinding some.

**Figure 9:**
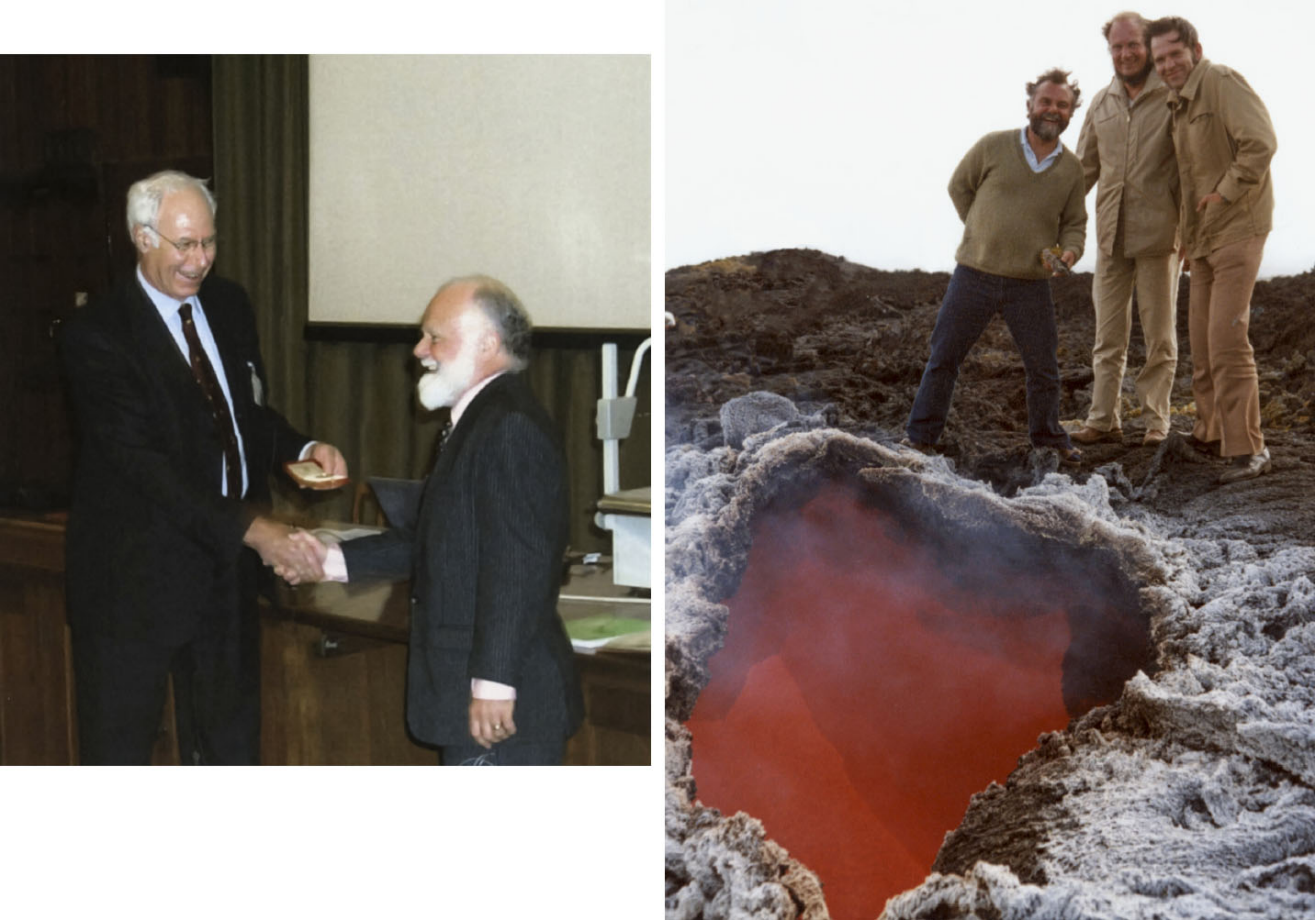
left: Nigel Weiss, President of the Royal Astronomical Society, handing me the gold medal. right: with Franz and Wojtek, after our emergence from a fumarole in Mount Etna.

Wojtek, Franz Deubner and I were taken up Mount Etna soon after an eruption. I was wearing thick-soled shoes, but still my feet were almost burning when I stood by the edge of the fuming crater. Mindful of the fate of Empedocles, I demurred to too close an approach (Figure 9). Instead I opted for a fumerole near the summit, and succeeded in (blindly) leading Franz into it. The sulphurous fumes were choking, and on emerging we were taken to a local volcanologist’s hut who plied us copiously with hooch; I suspect that its only medicinal function was to make us less aware of the pain. In the hut I spied an iron cup attached to a rod some two metres long, and asked its purpose. I was eager to try it, and after a great deal of persuasion the reluctant owner acquiesced under condition that I guaranteed not to lose it. It was to fish for lava. At close quarters the radiation from the lava river, flowing at about a metre per second, was intense, so my control was not as good as usual. I had imagined that after plunging the cup into the lava it would feel like being in thick treacle, but I was quite wrong: I was horrified to discover that it seemed rigidly imbedded in solid moving rock. I had only a split second to change my tactics, and fortunately I did succeed in withdrawing the cup. The lava was already solid as I pulled it from the flow. I now have the trophy amongst other memorabilia on the mantlepiece in my study at home.

**Figure 10:**
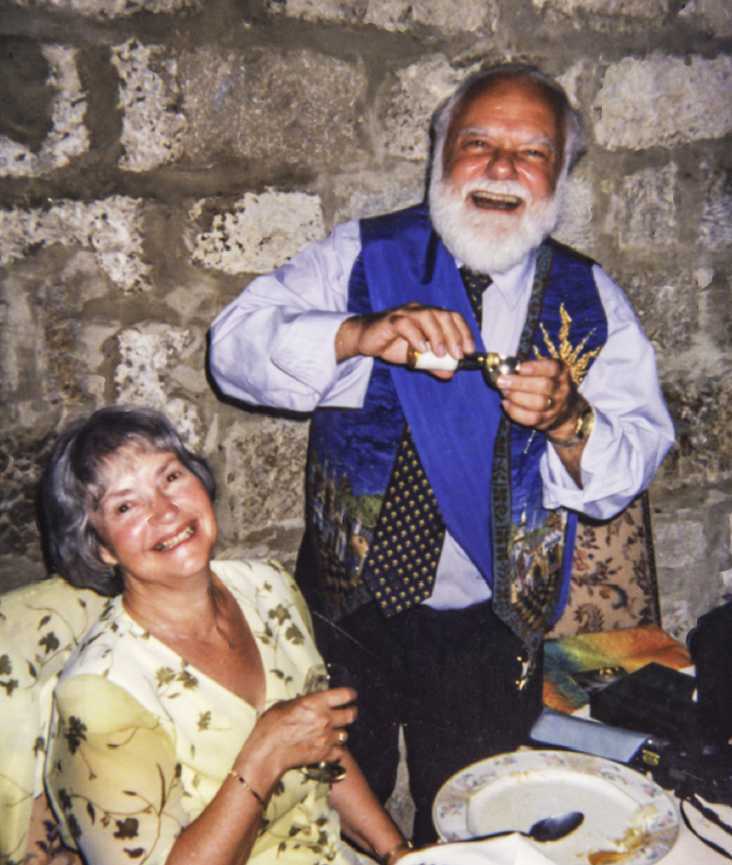
With Rosanne at the Stellar Astrophysical Fluid Dynamics conference dinner at Chateau de Mons (photo: Gérard Vauclair).

**Figure 11:**
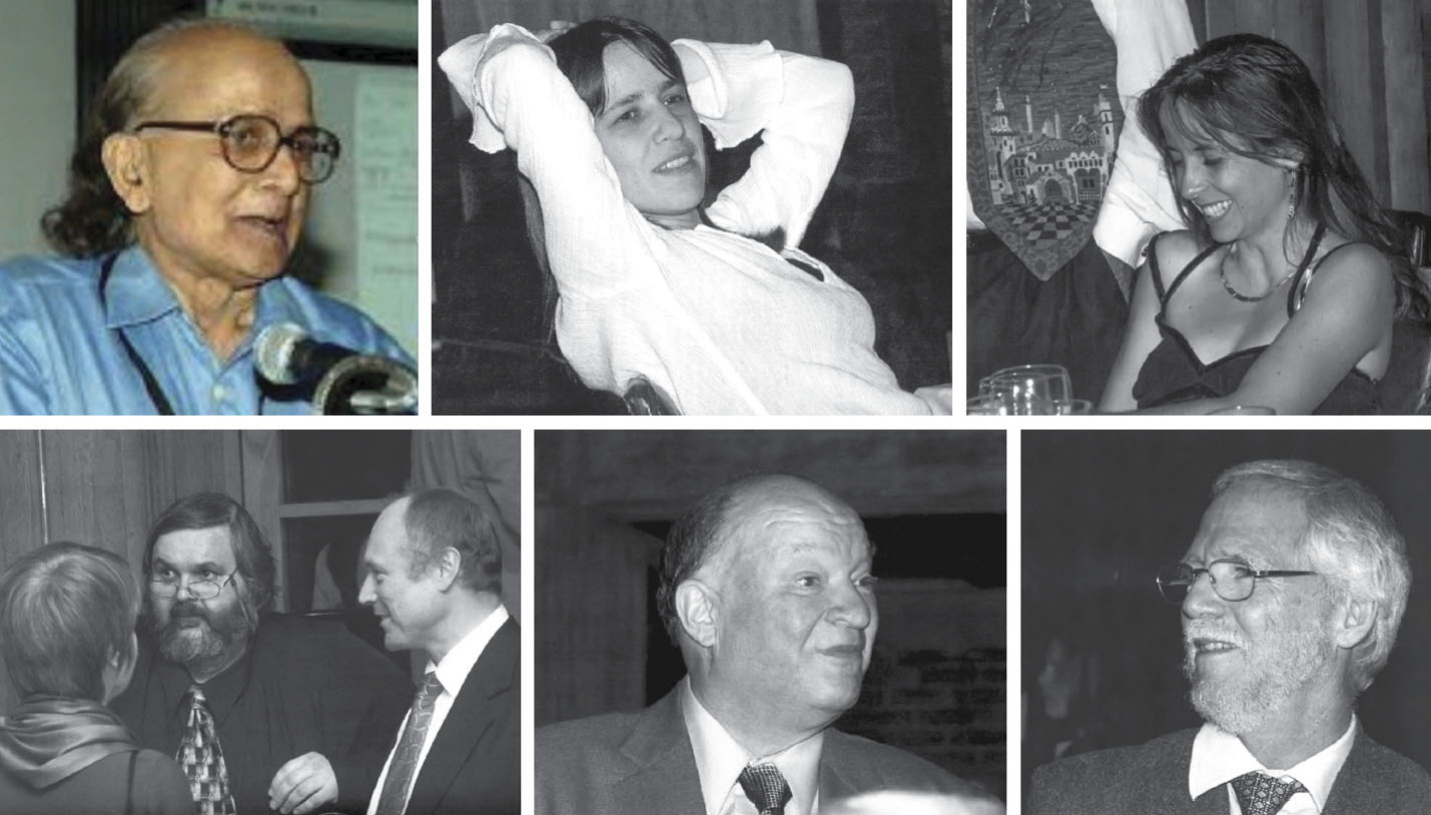
Top row, left to right: Kumar Chitre lecturing in Mumbai; Margarida Cunha and Pascale Garaud at the meeting on Unsolved Problems, Bottom row: John Lattanzio addressing Diana Roxburgh with Chris Tout, Werner Däppen, and Don Kurtz, also at Unsolved Problems.

**Figure 12:**
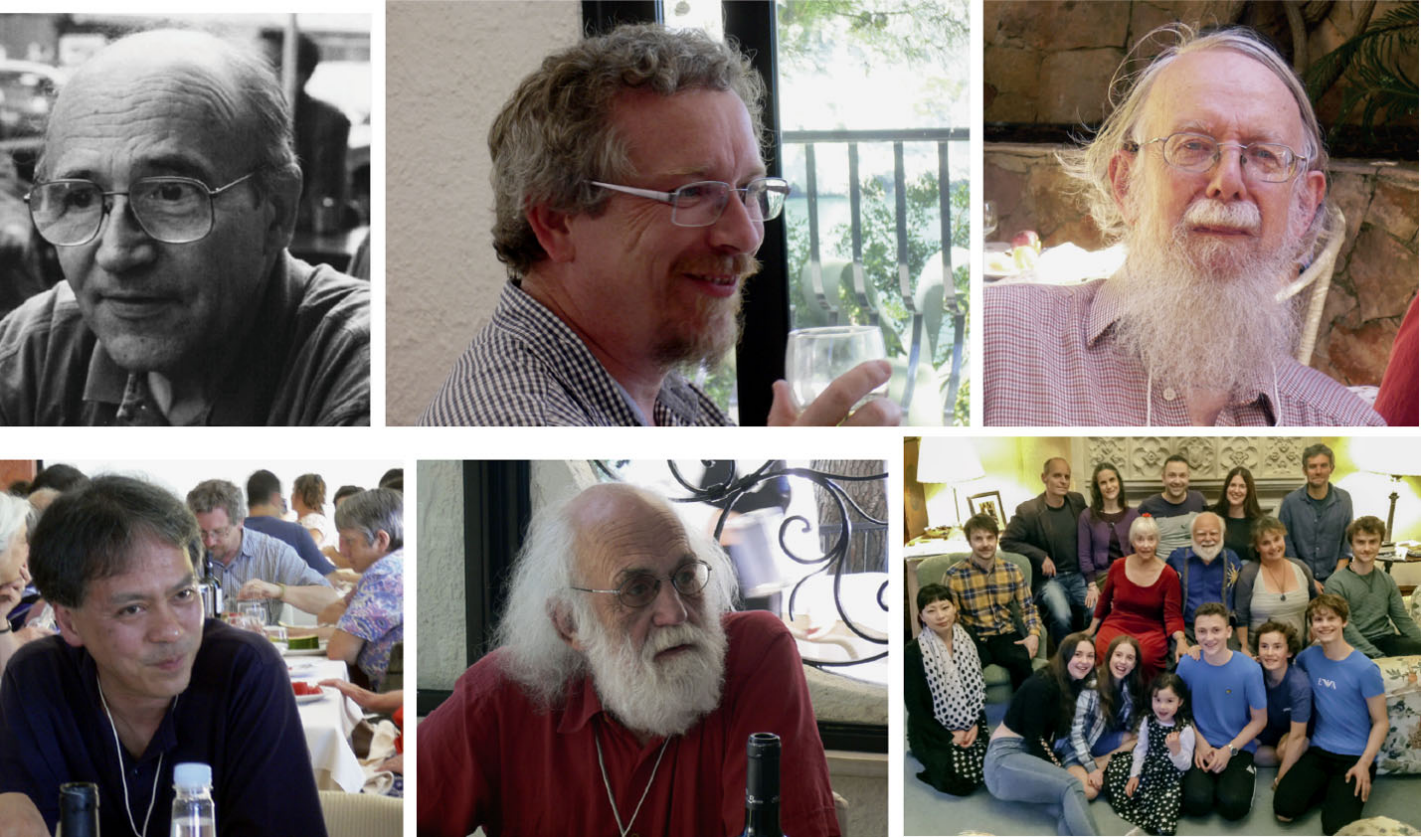
Norm Baker, collaborator, friend and godfather to my son Julian; Michael Thompson, Mike McIntyre, Takashi Sekii and Claus Fröhlich at the Gargano meeting on Waves in Physics; My family: standing: Jaimie and Kim McCabe, Russell and Tess Gough, Dom Clear; seated on chairs: Julian Gough, Rosanne, myself, Heidi Rose, Marcus McCabe; on the floor: Yurim and Caitlin Gough, Cara McCabe, Juno and Sam Gough, Mahni Clear, Callum McCabe.

Also, most memorable, were three conferences held in my honour. The first, entitled *Stellar Astrophysical Fluid Dynamics*, was to celebrate my 60th birthday. It was organised by Michael Thompson and Jørgen Christensen-Dalsgaard, who edited the proceedings (Thompson and Christensen-Dalsgaard, 2003), and Sylvie Vauclair. It was held at the Chateau de Mons in Condom (Figure 10), where I was invested, no doubt as a result of Sylvie’s influence, as a Mousquetaire d’Armagnac, the bibulous remnant of the guardians of Louis XIV, and I was taken to my favourite armagnac cellar, in Vic-Fezensac, where I was entertained by Marcel Trépout’s granddaughter (and subsequently given by the conference organisers armagnac distilled in my birthyear). In addition, Wasaburo Unno and Hiromoto Shibahashi kindly dedicated their paper on solar-cycle global warming to me, and Mike McIntyre played some piano music called DR which he had composed for the occasion based on Rosanne and my telephone number. The second conference, in 2007, was in Cambridge, organised by Chris Tout and Günter Houdek (Figures 11 and 7) with the help of several others (Stancliffe et al., 2007) on *Unsolved Problems in Stellar Physics*, which concentrated mainly on my interests. It was timed to anticipate my formal retirement, but was not, I was assured, valedictory. There have been enormous advances of late in our understanding of the mechanisms operating in stars, and this meeting opened up a yet wider arena pleading for our attention. Margarida Cunha and Pascale Garaud (Figure 11) organised the third conference, to celebrate my 70th birthday, entitled *Waves and Physics*. It was held in a beautiful resort on a high cliff in Gargano, overlooking the Adriatic sea. The meeting was less formal than the previous two, and devoted much time to the fundamental physics pertinent to stellar fluid dynamics. After the meeting, my family (Figure 12) took me across to the Amalfi Coast, to the village of Minori where my maternal grandfather was born. My relatives, whom I had never met, were extremely welcoming, and arranged a big gathering in a restaurant owned by a friend; they spoke no English, and we no Italian (except for my daughter Kim who had learnt Italian during the year explicitly to arrange our visit), but, as in any Italian community, communication never presents a problem. In addition, the KASC (Kepler Asteroseismic Science Consortium) meeting entitled *Seismology of the Sun and the Distant Stars 2016*, organised by Monteiro, Cunha, and Ferreira (2017) in the Azores, marked my 75th birthday, looking back on a conference with essentially the same name that I organised in 1985. Unfortunately, I was forbidden to attend for medical reasons. However, I was able to record a welcoming address that was projected at the start of the meeting.

## The Solar Tachocline

It was Ed Spiegel who aroused my interest in the tachocline – actually to make me aware that a tachocline in the Sun must even exist. We had both spent time in Woods Hole with experts in the dynamics of rotating fluids, much of whose knowledge had rubbed off particularly on Ed, who, unlike me, was intimately familiar with Ekman flow and the Holton layer – a boundary layer between a rotating stratified fluid in contact with a differently rotating rigid horizontal surface. When I was in New York, Ed was pondering the early spin-down of stars like the Sun. He adopted the common assumption that convection tends to make rotation rigid, thereby transmitting the magnetic torque imposed by the solar wind to the radiative zone beneath, inevitably via a thin Holton boundary layer: the original concept of the tachocline (Spiegel, 1972). My work with Donald Lynden-Bell in Cambridge had led me to believe that rotation causes Reynolds stresses to be anisotropic, and be largely responsible for the Sun’s equatorial acceleration, but I hadn’t contemplated seriously the implications for the radiative interior (Gough, 1966). So, in New York I was concerned about Ed’s boundary layer, although I had neither the time nor, I suspect, the ability to pursue it. Then, two decades later, Ed and Jean-Paul wrote the seminal paper on the subject (Spiegel and Zahn, 1992), which renewed my curiosity. After a great deal of discussion with Mike McIntyre, we both wrote the germ of an alternative idea that laid the foundation for subsequent advances, particularly by my friend Pascale Garaud, who started as my student, and her colleagues (e.g. Garaud, 2007; Garaud and Garaud, 2008; Garaud and Acevedo Arreguin, 2009). I have written recently in more detail on the matter (Gough, 2019), so I refrain from elaborating here, except to point out that a seismological investigation with Takashi Sekii to provide evidence for its veracity is underway.

## On Playing Devil’s Advocate

As a research student my work on magnetic inhibition of convection in sunspots naturally spawned an interest in its global manifestations: the dynamics of the solar cycle and the structural variation induced by the suppression of the thermal flux. With regard to the solar cycle, I should point out that I grew up in a den of dynamo theorists. I was therefore intrigued by Walén’s counter-suggestion of a magnetically restored torsional oscillation (Walén, 1947); after all, as Leon Mestel often impressed upon me, all stars must harbour large-scale magnetic fields. I therefore challenged the dynamo theorists to a competition: we both give a twenty-minute talk deriving the 22-year cycle period from ‘first’ principles to any group of (astrophysically unaware) physicists, I, admittedly with tongue in cheek, by estimating the strength of the Sun’s field from interstellar observations via the Batchelor (1950) vorticity-magnetic analogy,^18^ the dynamo theorist whatever he or she wanted to present. Nobody ever accepted the challenge.

In principle, a testable disparity between the hypotheses is that a magnetic oscillation would behave like a clock and therefore basically maintain phase, although the surface manifestation by sunspots would lag by an amount made random by the turbulent convection. On the other hand, the dynamo is normally thought of as being controlled within the convection zone, producing field reversals each induced by some mechanism with a stochastic element, permitting phase to wander. Bob Dicke (1978) and I (Gough, 1978b) independently carried out statistical analyses addressing the matter. Bob found no indication towards the dynamo model, I was unconvinced by the clock; the three-century sunspot record is not long enough to distinguish (Gough, 1990a). One might hope that there is some other feature that might distinguish between a primordial and a mainly dynamo-generated field, but that seems elusive (Byington et al., 2014).

During a sabbatical leave in Cambridge, Phil Goode and I highlighted a problem that the coherent magnetic oscillation hypothesis would need to overcome, namely that phase mixing can lead to serious damping. However, the process is negligible sufficiently close to an O-type neutral line, where it might just be dominated by differential gravity-wave dissipation akin to the driving of the quasi-biennial oscillation of the Earth’s atmosphere, described earlier in Section 9.

More convincing, in my opinion, is the tight phase coherence in the weaker oscillatory component revealed by Leif Svalgaard and John Wilcox (Svalgaard and Wilcox, 1975) in a half-century record of the geomagnetic field, possibly mirroring the rigid rotation of the Sun’s radiative interior, with a sidereal period of $26.70 \pm0.06$ days (Gough, 2017a). That, however, is quite different from an internal oscillation.

I must mention an interesting meeting with Jim Barnes and Peter Tryon in the time-keeping department of the National Bureau of Standards (NBS), as it was then, in Boulder, Colorado. They could generate qualitatively the behaviour of the sunspot cycle from appropriately Fourier-filtered white noise (Barnes, Tryon, and Sargent, 1980). Aside from the (arbitrary) timescale, the filter depended crucially on only one parameter, which, when chosen appropriately, reproduced both the variance of the intervals between successive maxima and the variance of the magnitudes of those maxima, together with grand minima. Moreover, it generated an apparently random time series when the actual sunspot record was passed backwards through the algorithm; when that time series was used as input to the forwardly running algorithm it reproduced the original record, of course, and when run beyond, with no further input, it generated the best, in some measure, prediction for the future. I was so excited that I hastened up the mesa that overlooked NBS to the High Altitude Observatory (HAO) to report the result to the dynamo theorists. On arrival, I was stunned by a complete lack of interest, because, I was told, the NBS exercise contained no (explicit) physics. But that was why it was so interesting: if a dynamo theorist were to offer sunspot predictions as evidence for the theory’s veracity, the theory must surely perform demonstrably better than the NBS algorithm. Some years later, HAO ran, no doubt with tongue in cheek, an open competition to predict the next sunspot maximum. There were so many entries that several of them necessarily had to be close,^19^ even were they to have been produced by monkeys with typewriters, so being close provided scant information. The HAO entry, however, was informative.

Of profound interest in the solar cycle are the dynamical processes that cause it. Four decades ago, there was interest in the ratio $W \equiv\Delta{\mathrm{ln}}R/ \Delta{\mathrm{ln}}L$ as sunspot coverage changed, where $\Delta R$ and $\Delta L$ are the radius and luminosity variations. Originally it had been thought that once $\Delta R$ had been measured, the variation of $L$ could be inferred from a theoretical value of $W$. Little was it realised that $R$ would be more difficult to measure than $L$, and indeed today both $R$ and a reliable value of $W$ still elude us.^20^ Werner Däppen (1983) and I (Gough, 1981b) had independently carried out computations for various kinds of putative structural perturbations, suggesting that $W$ increases with the depth of the perturbation. Two decades later, improved observations suggested a location some $0.02 R$ beneath the photosphere, superficial by the standards of some heliophysicists, but, according to Peter Foukal (e.g. Gough, 2002), too profound for the ubiquitous school of solar physicists having a superficial realm of investigation. The issue remains unresolved. What we do have now is a good measure of the temporal variation of the total solar irradiance (Willson, 2014), and Peter and his colleagues have provided a plausible explanation of how the angular variation of radiation from active regions is responsible (Foukal et al., 2006). As I presented soon afterwards at a celebration in Davos of Claus Fröhlich’s seventieth birthday, that variation is responsible for the (temporal mean) solar luminosity being of order 0.15 per cent higher than that normally inferred by assuming the Sun to radiate spherically symmetrically.

## Semiconvection

Semiconvection is a macroscopic buoyancy-driven transport process in stars that takes place in regions that are linearly stable to local adiabatic perturbations. It can occur in the presence of a stabilising composition gradient when the temperature gradient would otherwise be unstable. It was originally discussed by Roger Tayler (1954), and then by Martin Schwarzschild and Jüri’s uncle, Richard Härm, who encountered a contradiction when applying standard procedures for determining the edge of a convection core in a massive star (Schwarzschild and Härm, 1958): when a helium-rich core is predicted to expand because the stratification in a surrounding shell has become convectively unstable, the natural assumption is that the newly unstable region is mixed with the core, leading to a reduction in the opacity-producing hydrogen abundance by the introduction of extra helium, and a concomitant lessening of the temperature gradient, rendering that shell no longer unstable. The outcome is typically presumed to be only a partial mixing of hydrogen at too slow a rate to transport significant heat. But what is the resulting stratification? Typical assumptions have been that the composition profile is such as to make the region neutrally stable to either adiabatic abundance-preserving perturbations (the so-called Ledoux criterion) or to perturbations in which the contribution to buoyancy from the abundance perturbations is simply ignored (a misapplied Schwarzschild criterion).

Compositionally variable convection occurs in the terrestrial oceans, and has been studied extensively in the laboratory. It was originally called thermohaline convection, and often still is, although Stewart Turner (1973) proposed the term double-diffusive convection to accommodate situations in which the buoyancy-producing agents are not necessarily heat and salt. Then linearly stable, non-linear convection can ensue if the amplitude of a perturbation is large enough. George Veronis (1965) explained how that could be: if a layer of fluid is provided with boundaries from which a stabilising gradient of salt and a destabilising gradient of heat can diffuse, after an imagined total mixing of a stably stratified state, destabilising heat diffuses into the layer faster than stabilising salt, and convection is maintained. There are other possibilities. An initially isothermal layer of water, stably stratified with salt, when heated from below can develop stacked layers of convection separated by stable diffusive interfaces. Nowadays these are often called staircases. If they could be understood, a smoothed version would no doubt be adequate for stars. The interfaces can be characterised by the stability parameter, $\lambda= \Delta_{S}\rho/\Delta_{T}\rho$, namely the ratio of the contributions to the density jump across an interface due to jumps in salinity $S$ and temperature $T$. An interesting outcome is the ratio $\chi$ of fluxes of heat and salinity. Laboratory experiments with large $\lambda$ and large Rayleigh number suggest that $\chi$ is insensitive to both; it is simply the square root of the ratio of the microscopic diffusion coefficients for salt and temperature.

It would be wonderful if that result could be extrapolated to stars: knowing the heat flux supplied by the nuclear reactions would then provide a robust determination of the flux of helium (replacing salt), and so permit an estimate of the stratification in a semiconvective zone. Indeed, the thinness of the individual ‘stairs’ would even render the Boussinesq approximation valid, adding confidence to the result. Jüri and I once set out to investigate the matter (Gough and Toomre, 1982), using the steady-state, single-mode representation of Rayleigh–Bénard convection that we had studied in New York with Ed Spiegel. There were more than a single class of solutions, which rendered our results uncertain. We did find a solution class with near-constant $\chi$ at the Rayleigh numbers achievable in the laboratory, but when extrapolated to stellar conditions there was substantial variation. So, we were unable to offer a recipe to stellar modellers.

Considerable further progress with astrophysical applications in mind has been made since that time, mostly with numerical simulation by Pascale Garaud and her colleagues (e.g. Mirouh et al., 2012; Wood, Garaud, and Stellmach, 2013) (see also Zaussinger and Spruit, 2013). But, so far as I am aware, there is no published simulation with compositionally dependent diffusion coefficients directly pertinent to massive-star evolution. Some time ago my student, Guillaume Bascoul, with my guidance, carried out some two-dimensional numerical simulations with a fluid whose thermal conductivity is solute-dependent broadly similar to a hydrogen–helium gas mixture. Initial conditions were motionless, and we enquired whether when perturbed the system evolved to a state that was closer to Ledoux or to Schwarzschild neutrality. There was no clear simple outcome. In particular, the qualitative form of the evolution was dependent on how the initial state had been perturbed. So, in keeping with my earlier work with Jüri, we found no usable recipe for stellar-model builders.

## Classical Stellar Pulsation

In New York I entered a collaboration with Norm Baker (Figure 12) to study the effect of convection on the pulsational stability of stars in the neighbourhood of the classical instability strip in the HR diagram, using my newly formulated mixing-length approach, as I called it at the time. It was a spare-time activity, because I was working principally with Ed and Jüri on Boussinesq convection, and Norm on stellar pulsations with his new postdoctoral associate, Wojtek Dziembowski. That was when Wojtek and I first met, and we became lifelong friends. Naively, Norm and I thought that using the local version of the convection theory would be simpler than the nonlocal version to which we intended to graduate in the future. But we were quite wrong: for us even the equilibrium stellar model was insoluble, because the buoyancy term in the equations governing the eddy dynamics introduces a nasty fan singularity (cf. Eberhart, 1976) into the governing equations via the turbulent pressure, rendering them well-nigh impossible to solve numerically. Bob Stellingwerf and I worked on the problem for a while without success; Bob wanted us to publish but, having failed to solve the problem, I declined, leaving Bob to proceed alone (Stellingwerf, 1976). Norm and I cut our losses by artificially replacing the gas-pressure gradient in the buoyancy term with the total pressure gradient, as had Henyey, Vardya, and Bodenheimer (1965) before us, which obviated the singularity. We then found that convection appeared to stabilise the pulsations of RR Lyrae stars in the vicinity of the observed red edge of the instability strip (Baker and Gough, 1979). Later, with my student Neil Balmforth and postdoc Bill Merryfield, evidence was found that pulsationally induced variations in the turbulent pressure play a significant role in the destabilisation of Miras (Balmforth, Gough, and Merryfield, 1990; Houdek and Dupret, 2015), as I had suggested in my PhD dissertation, although I gather that matter remains controversial.

The work with Norm was carried out in widely separated bursts, mainly during short visits that I made to New York in the decade following my postdoctoral experience, which is why it took so long to publish. I used to stay with Norm in his apartment in Minetta Street in Greenwich Village, sleeping in a front room overlooking a night club called the Fat Black Pussycat; at the end of the road, 50 metres or so away, was a small supermarket, which took noisy deliveries very early every morning, always waking me up. Years later, I saw the film *Serpico*, about a policeman who lived next door to Norm. I was intrigued when in the film the NY police raided the night club early in the morning, and I could see at the edge of the screen the lorry whose winch had habitually awakened me. Norm and his wife, Doris, owned a farmhouse in upstate New York, which we visited together on occasion. They had a well into which some debris had fallen. I intended to climb down to remove it, but Doris was adamant that it was too dangerous, particularly because she and Norm had no way of fishing me out should I have fallen. However, the challenge was overwhelming; I had never climbed down a well before. I realised that I could ease my way down with my back against one side of the wall and my feet against the other – in my youth I had climbed a closely separated pair of pine trees in that manner – so Doris had little choice. However, she insisted that I take with me a lighted candle, to which I agreed: a lack of oxygen was more likely than a surfeit of methane. I reached the water unscathed, and have returned to tell the tale.

## Rapidly Oscillating Ap Stars

In the spring of 1982 Donald Lynden-Bell returned from one of his frequent visits to the University of Cape Town, and reported to me his meeting with a bright young observer who had discovered a new class of oscillating star. It was Don Kurtz (Figure 11), who was subsequently to become a very good friend. His discovery was of rapid oscillations of Ap stars (Kurtz, 1982), magnetically spotted chemically peculiar stars that appeared to be supporting just a few high-order, low-degree acoustic oscillations with a common axis inferred to be aligned with the magnetic field. The phenomenon immediately raised some interesting questions: What drives the oscillations, and why are so few modes excited? In particular, why are there oscillations of HD83368 with frequencies in a 2:1 ratio? Related to that is to ask what properties of the star are necessary for the oscillations to occur; for example, are the anomalous abundances necessary to support them? Also, how does the magnetic field distort the oscillation eigenfunctions, and what limits the oscillation amplitudes? And why are the oscillations aligned with the field?

Soon afterwards, Noël Dolez visited Cambridge from Toulouse, and we embarked on addressing the first and the last questions. Although we knew that a sufficiently strong, principally dipole, magnetic field would induce a tendency to align axisymmetric oscillations, we were concerned that it would be unable to resist Coriolis precession, particularly when the axis of the field lay almost in the equatorial plane, as some observations had indicated. We did, however, learn that the eigenfunction of an appropriate high-order mode could resonate with the classical driving region, and, moreover, that a mode of twice the frequency could resonate similarly, providing a hint of the 2:1 frequency ratio discovered by Don in HD83368 (Dolez and Gough, 1982). However, we did not actually find pulsational instability, partly because, it transpired, opacities of the day were deficient.

A sequel to this occurred at a conference in Catania the following year. Motivated by Don’s observations, my student Peter Taylor and I had evaluated the splitting of p-mode degeneracy by a combination of rotation and a magnetic field. I was to present the results in a session immediately following an introductory talk by Wojtek Dziembowski. To my horror, Wojtek gave what was essentially my talk, reporting on an analysis he had carried out with Phil Goode (Dziembowski and Goode, 1984). I had to think fast, and started by describing the situation in which I now found myself (to the amusement of the audience). Playing for time I asked Wojtek if I could borrow one of his transparencies for illustration. He obliged, of course, but when I saw it projected for the second time I realised that it contradicted mine. Now the audience was even more amused. I pointed out, half jokingly, how ‘scientific truth’ is decided democratically, and submitted our works to the vote. I voted for Wojtek, whereupon Wojtek responded by voting for me. No one else stirred. Eventually Jørgen Christensen-Dalsgaard, the session chairman, stood up and explained how as my student he had come to appreciate intimately how good a theorist I was, and consequently voted for Wojtek. By then I had recovered sufficiently to give a talk on a physical ‘explanation’ of what Peter and I had determined (Gough and Taylor, 1984). Yet our disagreement with Wojtek was unresolved. It was some time later that an error was revealed (Goode and Dziembowski, 1984), fortunately in time for appropriate revision for the Catania proceedings, and thereby informing about the value of democratic truth.

There followed a spate of detailed investigations into the acoustic frequency splitting brought about by a combination of rotation and a rigidly imbedded magnetic field (e.g. Dziembowski and Goode, 1985; Shibahashi and Saio, 1985b,a; Takata and Shibahashi, 1994, 1995b; Bigot and Dziembowski, 2002). One of the highlights, in my opinion, was a comparison by my student Margarida Cunha (2001) of the observed frequency distribution of the roAp star HR 1217 observed by Kurtz et al. (1989) with a theory (Cunha and Gough, 2000) that had evolved from earlier work with another of my students, Michael Thompson (Gough and Thompson, 1990), leading her to predict the existence of a ‘missing’ mode. A search with the Whole Earth Telescope, led by Don Kurtz (Kurtz et al., 2002), followed, and the mode was found.

There has been little interest in the geometrical implications of Coriolis precession in the presence of equatorially aligned fields, nor in the reason for the presence of magnetically aligned modes and the absence of unaligned modes, all of which I have regarded as essential for an understanding of the roAp phenomenon. These issues relate to the stabilisation of convection by the magnetic field in the spots (Gough, 2011) facilitating element segregation and enhancing overstabilisation of aligned modes (Balmforth et al., 2001), precessing or not (Gough, 2012). I have spent quite a long time investigating with my postdoc Mike Montgomery the little-studied mode distortion (but see, e.g. Takata and Shibahashi, 1995a), concluding that it is probably too weak to be important. As for the nonlinearity that limits the oscillation amplitudes, which is too tiny to modify the driving well beneath the photosphere as it does in classical pulsators, and too great for solar-like stochastic forcing to be plausible, I have concluded, tentatively, that it must be energy leakage by wave steepening in the atmosphere (Gough, 2013), extreme cases of which had already been observed (Kochukhov and Ryabchikova, 2001; Shibahashi et al., 2008).

## Some International Collaborations

**Figure 13:**
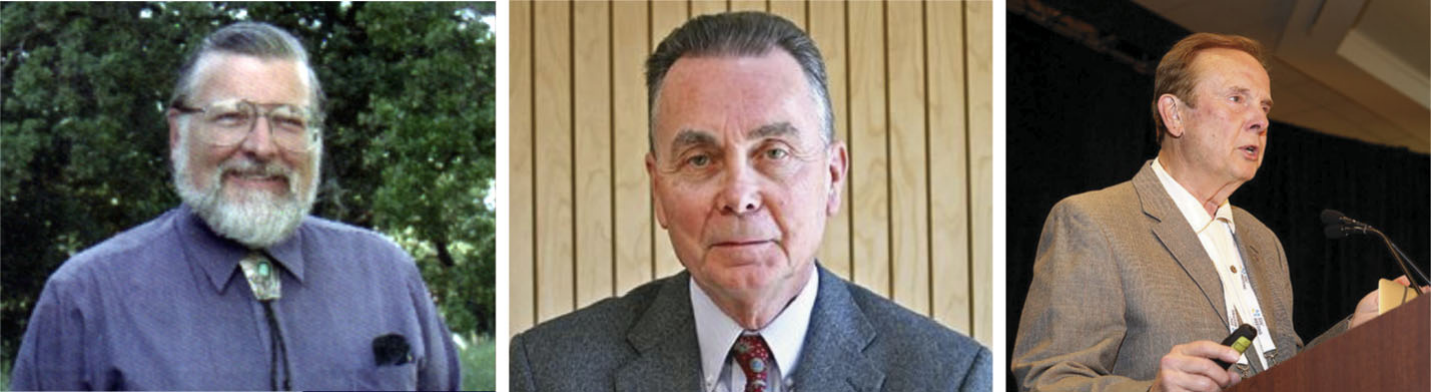
From left to right: Phil Scherrer on his 60th birthday; Jack Harvey on the occasion of his award of the Arctowski Medal of the National Academy of Sciences; Tom Duvall, delivering the George Ellery Hale Prize lecture to the American Astronomical Society.

Aside from interactions with my students and postdocs in Cambridge, collaborations have been mainly abroad. Many were continuations of work started in Cambridge. Most notable amongst those to which I have not already alluded have been with Kumar Chitre (Figure 11), Hiromoto Shibahashi (Figure 7) and Phil Scherrer (Figure 13).

I first met Kumar at a winter school at the Royal Greenwich Observatory, when I was a student and Kumar a lecturer at the University of Leeds. We jelled immediately, and on most days talked well into the night. Subsequent meeting was rare until my first visit to India in 1984. Then, we started a long-term collaboration of mainly scientific discussion, with a few publications emerging on the way, mainly with the help of Antia at TIFR. That work has been concerned mainly with solar rotation and the possible role played by a large-scale magnetic field. I visited TIFR several times, and in recent decades Kumar has visited me almost annually at IoA. I was appointed to Distinguished Visiting Professor at the University of Mumbai in 2015, where we were joined by Bhooshan Paradkar, continuing a development of an approximate eddy-ensemble treatment of convection in rotating, arbitrarily shearing flow. Very sadly, Kumar died in January 2021, and progress has halted, I hope only temporarily.^21^ It was a great blow to have lost a friend of almost an entire lifetime.

Japanese collaboration started with Yoji Osaki during our contemporaneous sabbatical leave in Nice. It was probably as a result that I was invited to the celebration of Wasaburo Unno’s retirement from his ‘first life’, as he left the University of Tokyo for his ‘second life’ at the University of Kyoto. There I met Takashi Sekii, whom I invited to Cambridge to work on seismological inversion. I should mention also my research student Heon Young Chang, from South Korea, who studied ways of analysing seismic data while recognising putative temporal variation in the background structure of the star (e.g. Chang and Gough, 1995). Takashi was very helpful in this endeavour, dissolving communication impediments by teaching me how in eastern cultures what is meant is not necessarily what is said, but rather how it is said. Umin Lee added to my visitors from Japan, and later Masao Takata. After my visitors’ appointments had expired I enjoyed several fellowships in Tokyo, making it possible to spend time also with Hiromoto Shibahashi, with whom I shared an interest, together with Don Kurtz, in the dynamics of rapidly oscillating Ap stars. Sadly, my last visit to Tokyo was a disaster. Michael Thompson was visiting simultaneously, and we shared an office. On returning from a stressful trip to NCAR, where he was Deputy Director, he said he was feeling unwell. I responded saying he looked unwell. “Thanks”, he responded. A few hours later he was dead. We were all deeply distressed; we had lost a very good friend. I left behind two unfinished long-term investigations: seeking with Takashi a seismic signature of putative magnetic penetration through the tachocline, and with Masao the acoustic radius of the Sun and its possible variation with the cycle. I pray that in time both will see the light of day.

In the late 1970s I visited John Wilcox and Phil Scherrer at Stanford University to discuss their seismic observations. That led to a new collaboration, which was sadly diluted by John’s tragic premature death. I was considering a professorial appointment at Stanford, but ties in England prevailed. Nevertheless, my contact with Phil, and his wife Debbie, was maintained, not least through my involvement at the European Space Agency in arguing for SoHO, and as a consultant to NASA supporting MDI, for which Phil became PI. My relation with Phil, both scientific and social, developed substantially, and with Phil’s generosity led to annual visits to Stanford that I enjoyed immensely, and continued until I contracted severe sepsis in 2018. The visits also afforded Rosanne and I opportunities to experience the environs, especially Napa and Sonoma Valleys, and to go to nearby UC Santa Cruz to see my friends and former students, Doug Lin and Pascale Garaud.

It was in part my living in the UK, an environment in which, in the early days of helioseismology, showed rather limited interest in my work, that stimulated my collaborations internationally. Neverthless, I have interacted more generally with some local colleagues, and sometimes we have been moved to go to press. I have already mentioned Jim Pringle. Another example is a collaboration with Andy Fabian questioning a paper suggesting that the purported 160-minute oscillation of the Sun is driven by gravitational radiation from Geminga (Fabian and Gough, 1984). We had a refreshing excursion from our domain of comfort, briefly entertaining an idea that the Earth’s atmosphere might have modulated a spectrum line of ozone, leading to a false Doppler interpretation of the Geminga observations. We worked hard, and consulted Brian Thrush, a distinguished atmospheric chemist, who assured us that the idea was not obviously ridiculous. However, Philip Campbell at $\mathit{Nature}$, an atmospheric physicist himself, sent our paper to a referee who flatteringly reported that our idea was very interesting, highly original and well argued, but that he would apprise us of a pertinent chemical reaction rate (one that we had sought unsuccessfully ourselves) which he was sure would cause us to withdraw our suggestion. His information did not disprove our proposition, but it made it highly unlikely. Accordingly, we withdrew.

Another local collaboration was with Neil Balmforth and Chris Tout on the pulsations of Arcturus (Balmforth, Gough, and Tout, 1991). Chris was subsequently the kind instigator of my ‘retirement’ conference at IoA.

## Further Helioseismology: The Neutrino Problem and General Relativity

As I have already mentioned, of the major unresolved issues concerning the Sun in those early days, there were two, perhaps one could say three, that stood head-and-shoulders above the others: the so-called solar-neutrino problem, associated with which was the matter of the helium abundance, and the test of the theory of General Relativity from orbital measurements of Mercury and spacecraft, the latter by radar ranging. I summarised the contributions that helioseismology made to those issues in my welcoming address (Gough, 2017b) to the conference in the Azores on *Seismology of the Sun and the Distant Stars II*, and I unashamedly repeat some of that here.

Establishing that the convection zone was deep by the standards of the day implied, according to theoretical models of the Sun, that the zero-age-main-sequence helium abundance $Y_{0}$ was relatively high – of the order of 25 per cent by mass – which was comforting because an abundance low enough to reproduce Ray Davis’s neutrino counts in a standard solar model would have required facing the problem of explaining how it could be substantially lower than what was believed to be the cosmic value immediately after the Big Bang (see e.g. recent by Peimbert, 2008). However, because there was evidently a serious problem with the production of neutrinos in the Sun, the theory of stellar evolution, at least as applied to the Sun, was evidently fragile. A deep convective envelope was only an indirect and questionable indicator of the conditions under which neutrinos are being produced in the energy-generating core. The least-squares calibration of solar models that Jørgen Christensen-Dalsgaard and I carried out using the frequencies of low-degree p modes obtained from full-disc Doppler observations was really quite crude because it paid no regard to what aspects of the frequency spectrum related specifically to the core (Christensen-Dalsgaard and Gough, 1981). It should be said that in any case the outcome was ambiguous, because there were two preferred solutions, each having different mode orders $n$ assigned to the peaks in the power spectrum: one had low $Y_{0}$, the other high $Y_{0}$. The latter was superior, but far from perfect. Knowing $n$ was needed to settle the matter.

Earlier we had found evidence for $Y_{0}$ not being unexpectedly low from what are now called the large and small frequency separations determined from the full-disc observations of Grec, Fossat, and Pomerantz (1980), the former providing a measure of the acoustic radius of the Sun, the latter a signature of the core. To a first approximation, neither requires knowing $n$. Jørgen and I compared the observations with new theoretical values obtained by taking the influence of the Sun’s atmosphere more carefully into account (Christensen-Dalsgaard and Gough, 1980). We concluded that there was no reason to believe that the Sun is helium deficient. Nevertheless, some people, such as George Isaak (1980), adhered to a contrary opinion that was based on an earlier analysis of the Birmingham group’s observations (Claverie et al., 1979).

Later, together with Werner Däppen (Figure 11) and Michael Thompson (Figure 12), an almost direct measurement of $Y$ in the convection zone yielded a value $0.233 \pm0.003$, consistent with a subsequent evaluation of $0.234 \pm0.005$ by Dziembowski, Pamyatnykh, and Sienkiewicz (1991). This value is less than the estimates of $Y_{0}$, the difference, as suggested by Dziembowski et al. and demonstrated by Christensen-Dalsgaard, Proffitt, and Thompson (1993), being accounted for by gravitational settling over the main-sequence life of the Sun.

The reason that mode orders $n$ could not be identified observationally is that the full-disc spectra did not extend to sufficiently low frequency for it to be possible to extrapolate reliably to $n=0$; fortunately, at least the degrees $l$ were known, as Jørgen and I had shown from the pattern of the small frequency separations.

It was Tom Duvall and Jack Harvey who came to the rescue (Duvall Jr and Harvey, 1983): I had persuaded them to make crucial observations of modes of intermediate degree that connected the full-disc data to the high-degree modes whose lowest-order branch (f modes) in a $k - \omega$ diagram, which, as I mentioned in Section 9, is essentially independent of the internal structure of the Sun, and whose order $n$ could therefore be identified unambiguously. The modes were then traced to sufficiently low $l$ to permit extrapolation to $l=0$. The outcome completed the low-degree seismic calibration, selecting the solar model with the higher $Y_{0}$. That preference was reinforced by observations having low spatial resolution carried out by John Wilcox and Phil Scherrer. They were differencing Doppler measurements from an outer annulus and the central disc, but initially had been unable to resolve individual frequencies until Jørgen and I pointed out that they were sensitive to more values of $l$ than are the full-disc observations, and therefore required observations of longer duration to disentangle them: full-disc Doppler observations are sensitive principally to modes with $l = 0,\,1,\,2 \,{\mathrm{and}}\,3$; John and Phil extended the range to $l=5$ (Scherrer et al., 1983).

My interaction with Jack and Tom, and with John (until his untimely death) and Phil, continued. Those early days were the most exhilarating in my scientific life, Jack and Tom, particularly, directing observations towards answering questions that I posed rather than pursuing what they could measure the most accurately, and I carrying out calculations stimulated by their new observations in the hope of shedding more light on the matters in hand, all of which, of course, raised new questions: it was textbook scientific method in action. It has been fascinating to investigate the ramifications of the new preliminary data, with the understanding, of course, that the outcome could not be relied upon until the analysis of the data had been confirmed. Working at the border of credibility, with appropriate caution, is essential for advancement.

Tom and Jack refined their observations – or perhaps just the analysis of their pre-existing data (Duvall Jr, 1982; Harvey and Duvall Jr, 1984) with a view to measuring the acoustic stratification of the entire Sun. They had imaged the Sun with a cylindrical lens whose axis was aligned with the axis of the Sun’s rotation, thereby extracting just the zonal and near-zonal modes that provide a weighted longitudinal average. It was now possible to invert the data to obtain a direct estimate of the sound speed throughout all but the innermost regions of the Sun. With the help of Jørgen, who provided theoretical solar models with which to compare the seismological analysis, together with considerable additional insight, a quite detailed picture of the solar interior was emerging (Christensen-Dalsgaard et al., 1985). In particular, it convincingly ruled out solar models with low $Y_{0}$, although, interestingly enough, that inference was not immediately appreciated by the solar-neutrino community who appeared to be unaware of the robustness of conclusions derived from such simple physics as acoustics. An interesting suggestion arising from the work – only a suggestion at the time because it concerned much more complicated physics – was that under conditions near the base of the convection zone the opacity incorporated in the theoretical models was about 20% too low. Five years later that suggestion was verified. The body of helioseismological evidence now augmented the most compelling evidence towards locating the resolution of the solar-neutrino problem: it lay in nuclear or particle physics, and not in the theory of stellar structure and evolution.

In a stroke of genius Tom Duvall (Duvall Jr and Harvey, 1984) thought of rotating their cylindrical lens by $90^{\mathrm{o}}$. Although that did not collapse the solar image precisely onto lines of longitude, it almost did so in the equatorial regions where the sectoral-mode amplitudes are greatest. In that way, Tom and Jack were able to measure rotational splitting. We were joined by other theorists, Wojtek Dziembowski, Phil Goode and John Leibacher, to infer the Sun’s angular velocity almost to the core (Duvall Jr et al., 1984a). Because the inversions, primitive as they were, were relatively straightforward, publication (Duvall Jr et al., 1984b) came before that of the sound speed. By far the dominant feature of the inference is that the interior of the Sun is rotating almost uniformly at very roughly the photospheric equatorial rate, and not a great deal faster, as many theorists of the time suspected. Indeed, it immediately settled a contentious issue with Bob Dicke (1964, 1967), who had argued, hardly convincingly (Bretherton and Spiegel, 1968), from his inference of the high oblateness of a measure of the visible solar disc, that the Sun’s core rotates with a period no greater than a terrestrial day or so, and that the associated oblateness of the gravitational potential supported his scalar-tensor theory of gravity against General Relativity. A value of the quadrupole moment $J_{2}$ of the Sun’s exterior gravitational potential, $2 \times10^{-7}$, was calculated from our seismological inference; it is not inconsistent with General Relativity. Because the Sun’s angular velocity is inferred from frequency splitting by advection of seismic waves, which depends linearly on the (small) angular velocity, and not on the much smaller, quadratic, centrifugal force, the inference of the value of $J_{2}$ is much more robust than Bob’s estimate, not to mention the difficulties that had confronted Bob in interpreting the apparent shape of the photosphere.

At Wojtek Dziembowski’s recommendation, I welcomed Sasha Kosovichev to work with me in Cambridge during the following decade. Our aim was to advance our overall knowledge of the Sun’s interior, with particular emphasis on the hydrostatic stratification (e.g. Gough and Kosovichev, 1988, 1990; Gough, Kosovichev, and Toutain, 1995) and its implications (Kosovichev et al., 1992), and we also addressed several less popular issues such as enquiring whether the Sun rotates about a unique axis throughout (Gough and Kosovichev, 1993; Gizon, Appourchaux, and Gough, 1998).

I had a wonderful journey through those early days of the development of the subject, and I have enjoyed sharing it with so many of my friends. Many astronomers and physicists were impressed by what had been achieved, and I was honoured by the award of the James Arthur Prize by Harvard University in 1982 and the William Hopkins Prize by the Cambridge Philosophical Society in 1984 for establishing the discipline of helioseismology. I used the prize money to buy a new hi-fi music system, which I enjoy to this day. Subsequently, together with others, I was named by the Queen as a Pioneer to the Life of the Nation.

## Broadcasting

Sharing one’s knowledge, and one’s excitement in acquiring it, is, in my opinion, one of the duties of any scientist who, ultimately, is supported by the rest of society. To this end, aside from public lectures and lectures to enthusiastic schoolchildren, I have made many radio broadcasts and several TV appearances. Specialist lectures are also important, and I mention here just one: a Colloquium Ehrenstestii in 2004 at the Instituut Lorenz in Leiden. On approaching the modern building I noticed that it leans dramatically at such an angle that had I been running towards it at the typical speed of a free electron in the core of the Sun, relativistic aberration would have made it appear vertical. I mentioned this in my lecture, to such audience delight that my remark was incorporated for a while on the title page of their website. After the lecture I had the honour of signing the wall of the old institute building, joining an array of the most distinguished physicists of the twentieth century.

Early in my career, radio interviews felt an almost solitary activity: the British Broadcasting Corporation (BBC) rented a tiny windowless soundproof room in Cambridge with a dedicated telephone line to London. An interviewee had to collect the key from the caretaker of a local theatre, and (with some trepidation) activate the equipment in the hope of establishing contact with the main studio. It often required intervention by engineers in London, yet it was always successful in the end. The hi-fi outcome impressed on me the quality of standard telephone when nobody else is sharing the line. Subsequently, the BBC set up a local radio station in Cambridge, and now there are friendly staff on hand to organise everything, and offer refreshment too.

Television programmes are more challenging. My first was about solar variation, in a documentary series called Horizon (Nova in the USA). The producer told me that he was very rigorous about presenting the science correctly. In trying to explain how internal properties of the Sun can be established from seismic frequencies, I intended to demonstrate how the spectrum of the sound from an oboe (which has a conical bore) is expected (at least according to elementary theory) to differ from that of a clarinet (which has a cylindrical bore). One of my daughters and her schoolfriend came to be filmed playing the instruments (what nepotism!). To my dismay, when a trial demonstration was carried out, the real oboe’s spectrum had twice as many frequencies as expected. This was only a day or two before the final filming. However, I found that if the oboe were played with the main body of the instrument removed from the mouthpiece, the spectrum looked theoretically perfect. Uneasily, I reported my findings to the producer, and was relieved when he declared that it was more important for an analogy to demonstrate the correct science than it is to demonstrate the analogy correctly. For me the most memorable phase of the production was the final dubbing, when I witnessed the stunning skill of the dubber in replacing, for example, the sound of the motor driving a telescope on Mount Wilson with that of the rumbling of Mount Vesuvius played at 3.2 times the correct speed, producing a more plausible impression as the camera moved.

TV can be hazardous. I well recall being filmed from the top of a tower at Churchill College. The location is open to the elements, and the filming was on a severe winter’s day: I was wearing a sleeveless shirt appropriate for basking in the summer Sun, that Sun being provided by a powerful lamp precariously mounted on a flimsy scaffold erected in short order by the BBC when it was discovered that the sky in Cambridge was overcast that day. The filming took several hours, and I was left with a very bad cold from which it took long to recover. Yet sometimes interesting new knowledge can be acquired from being interviewed. For example, my first interview in India was by an elegant lady who sat on my left with the camera to her left. It made me realise how common a configuration that must be, for my interviewer was able to have her notes on her thigh, concealed from the camera by her sari, which is always draped over the left shoulder, and in this case also over her left knee.

Perhaps my most memorable experience was at the end of a day filming at the Institute of Astronomy. The film crew had retired to my office, where they enquired of the purpose of two large springs on my filing cabinet. They represent the electrostatic force produced by protons, the protons being modelled by strong magnets to which the springs are attached. I used them to demonstrate the interactions initiating the p–p chain in a star. I would throw one at the other; typically, they would bounce apart, but if aimed sufficiently accurately (which required some practice) the magnets might collide, locking together and ejecting a positron (which had been loosely attached with another spring to one of the magnets). The producer was intrigued. There was just film enough remaining in the camera for a final couple of minutes (for preferred programmes the BBC used film instead of the more common electronic detectors because it has much higher resolution), and the producer wanted to use it. However, I was out of practice. I recall vividly having the cameraman and his assistant, the producer and the boom operator, all crammed into one end of my tiny office, I nervously about to make my only allowable attempt at an ‘accurate’ collision – oh, additionally the Institute Director of the time, Martin Rees, who had been passing by and had wheedled his way into my office, was playfully jumping up and down and pulling grotesque faces in a (fortunately failed) attempt to distract me. Amazingly, the collision succeeded, and the cameraman even successfully followed the emitted positron as it rolled across the floor; the associated neutrino moved too fast to be seen.

The BBC supply many foreign TV companies, often with its unused footage. So occasionally I hear from distant friends years afterwards of TV appearances about which I was quite unaware. Lectures sometimes leave a similar legacy. I was once surprised whilst wandering in the Kruger National Park to encounter a young lady whom I did not know yet who knew who I was: she had attended my Bishop Lecture at Columbia University a few years earlier. Subsequently, I encountered out of the blue a man who remembered my cooking spaghetti at my Wernher von Braun Lecture to NASA, and another who recalled entering the auditorium for my Halley Lecture at the University of Oxford to the blaring of Gong.^22^ I am always careful in such circumstances not to enquire whether the scientific content of my talk had been remembered.

## Interpreting Helioseismological Inversions

Inverting seismic data is not just mere technicality. It is also art. It requires judging what aspects of the formal results to believe, for in reality they are all subject to errors in the data. If one really knew the statistics of the errors one could go a long way towards achieving reliable inferences, but alas observers often pay too little attention to what many of them perceive as a less profitable aspect of their work. Perhaps they are right. I recall here an important early exercise with artificial data to mimic inferring the internal angular velocity throughout the Sun. It was presented by Jørgen and me at a conference organised by Roger Ulrich (Christensen-Dalsgaard and Gough, 1984). The purpose in those early days was to convince the audience of the promise of helioseismology, and persuade more people to join us in this new and exciting discipline.

There were three stages to our exercise: first, Jørgen secretly invented an angular velocity $\Omega$, assumed to depend only on radius $r$, and from it, together with a model of the spherically averaged Sun, computed the frequencies of various sets of seismic modes to which he added random errors, and made the frequencies available to me without even a hint of $\Omega$ or the errors in the data; next, I inverted the frequencies and plotted my inferences on a transparency,^23^ and informed Jørgen of the scale, but nothing more; finally, Jørgen produced a transparency of the exact $\Omega$ on the same scale as mine. Jørgen and I did not communicate our results to each other before we made our public presentation at the conference. First, Jørgen explained what he had prepared, then I presented my inferences, finishing by showing my transparency containing optimally localised averages of my inferred $\Omega$, together with an indication of the resolving power and my estimate of the reliability. I remember my nervousness, for we had never before performed such an exercise, even in private. To be sure, I had carried out inversions of artificial data, but alone, so I had always known the true answer. This time was quite different. I was now acutely aware of the intellectual processes required for deciding what can be said from uncertain results. Finally, Jørgen overlaid the exact $\Omega$. Fortunately, his random errors were uncorrelated, and my inferences were correct. Our audience appeared to be impressed.

This exercise had taught me more than I had anticipated. So I decided to share it with my friends. That occurred first within GONG, the acronym for the ground-based seismology team: Global Oscillation Network Group. I had encouraged the original proposal to the National Science Foundation for a ground-based network of helioseismic observatories (although I did not contribute to writing it) and, after it was successful, set up an inversions team to develop methods for analysing the anticipated data. The first big exercise was in the nature of a hunt: a hare being chased by a pack of hounds. As hare, I computed a static solar model in hydrostatic equilibrium, and invented a latitudinally varying internal angular velocity $\Omega(r,\theta)$. I then computed a set of p modes that I thought GONG was likely to acquire; I supplied the eigenfunctions and eigenfrequencies, the latter with added uncorrelated random errors, together with the equilibrium model, to the dozen or so members of the team who wished to participate as hounds. I had taken great care to ensure high numerical precision – of order one part in $10^{6}$ – so that the hounds would not suffer uncertain numerical error. The hounds were told what I had done, but not how $\Omega(r,\theta)$ had been chosen nor the variance of the errors that had been added to the frequency data; they were also told the equations that I had used. The task of the hounds was to announce their inferences: their estimate $\Omega_{\mathrm{e}}$ of $\Omega$ and its uncertainty, together with a commentary on what aspects of their estimates they believed to represent the actual angular velocity.

The form of the angular velocity was chosen in the hope of sorting sheep from goats. For example, had a hound chosen to represent the latitudinal variation as a (truncated) series in Legendre functions, which was tempting, then they would have had to have kept many terms, because the coefficients declined with degree for the first half-dozen terms, chosen deliberately to lull the unwary into believing that their expansion had almost converged, but then they rose again, contributing to a qualitative modification to $\Omega_{\mathrm{e}}$. I might point out that finding an appropriate function $\Omega$ having that property was more difficult than the task that I had posed to the hounds.

There was a broad spectrum of responses, no doubt because in many cases this was amongst the first inversions the hounds had ever performed. I report here just the worst and the best. The worst was by a hound who had not even attempted an inversion. That didn’t matter. What did matter is that he purported to have checked the integration of the equilibrium equations – they contained a local mixing-length formalism with a well-defined, non-zero mixing length, which of course leads to stratification that is not differentiable at the convection-zone boundaries – he had (mis)used a sophisticated high-order integration routine that was valid only for highly differentiable functions. That led to a relative error of order $10^{-4}$, which I had already estimated before the meeting as being characteristic of common integration schemes that fail to incorporate correctly the mathematical structure of the system. (In anticipation I had taken care to provide the hounds with enough information to account for the discontinuity, should any have so chosen.) So, it was quite plausible that the hound in question really had carried out his computations in the manner that he had said. His big mistake, however, was to be so confident in his own work as to condemn vituperatively what he considered to be my irresponsibility in providing a model whose numerical inaccuracy was 100 times greater than what had been stated! I was bemused by his arrogance, but his accusation was beyond the pale. At the other end of the spectrum was a Great Dane, namely Jesper Schou. His graphical depiction of his inversion suggested that my model convection zone was not spherical. He didn’t mention it in his presentation, so I questioned him at the end. He responded that it was no doubt an artefact of his imperfect procedure. Actually, the model was indeed aspherical in the manner that Jesper had depicted, but Jesper’s confidence was not as great as his mathematical prowess. It demonstrated that careful judgement is an integral part of inference.

Another exercise worthy of mention was a blind attempt by Takashi Sekii to carry out a (linear) rotational-splitting inversion of some artificial data that I had constructed, which I used for illustration in a course I was giving in a summer school at the Astrophysical Institute of the Canaries. I told Takashi in advance that errors had been added to the theoretical eigenfrequencies, and suggested that he might estimate their magnitude. But I did not reveal that the errors were correlated. Takashi came up with a ‘solution’ that he sent me immediately before my relevant lecture, providing no time for me to see it in advance. It was many of his estimated standard errors from the correct result. Furthermore, the magnitudes of the errors had been considerably underestimated. The reason is that Takashi had assumed that they were uncorrelated, as was, and still is, the fashion with real data. So, the exercise provided another important lesson in interpretation. Takashi and I subsequently published a paper on the matter (Gough and Sekii, 2002), but it seems not to have been persuasive enough to influence observers. To be fair, the discrepancies in our paper were not as great as the original (Gough, 1996), because we designed the correlations to represent what we thought might arise in reality; the original correlations were deliberately tailored to yield wildly erroneous inferences. As had been the case for the first GONG hare-and-hounds exercise, designing the frequency errors was more difficult than the mathematical phase of the inversion process, but evidently not the interpretation.

I found these and other analogous exercises to be most rewarding, mainly because they were as much social activities as they were scientific investigations. They encouraged the enormous progress that was achieved at the end of the last century. It was heart-warming that my involvement was recognised by my election to Fellowships of the Royal Society and of the Institute of Physics. I was also made a Foreign Member of the Royal Danish Academy of Sciences and Letters, which I’m sure was a result of Jørgen’s influence.

## A Preparation for Asteroseismology

It is evident from the successes of helioseismology that valuable information can be obtained from the properties of the oscillations of the distant stars. Only modes of low degree can be identified, but the coefficients in the (smooth) asymptotic high-order frequency formula and the asymptotic oscillatory behaviour of frequency differences provide important diagnostic signatures that can be used for calibrating structural properties such as helium abundance $Y$ and its spatial variation without undue contamination by extraneous properties. Calibrations can be carried out with accurately computed theoretical frequencies; they do not depend directly on the accuracy of the asymptotic formulae, contrary to the opinion of some critics. Such calibrations have been carried out with Günter Houdek (Figure 7) for the Sun too, with $Y$ and the main-sequence age $\tau_{\mathrm{ms}}$ principally in mind (Houdek and Gough, 2007, 2011), the latest results being $Y_{0} = 0.250 \pm0.001$, $\tau_{\mathrm{ms}} = 4.602 \pm0.002$ Gy, ignoring systematic errors, although this is not the last word. My interest in an accurate estimate of the latter was to address whether the Sun is older or about the same age as the oldest meteorites. Sufficient accuracy has not yet been attained.

I have not contributed a great deal to asteroseismology in recent years. That has been Jørgen’s domain. Good general signatures are elusive. Nevertheless, I am delighted by the enormous effort that is being expended by others (cf. Chaplin and Miglio, 2013). The radial variation of angular velocity has led to important dynamical questions. Moreover, structural calibration using signatures that are not ideally appropriate, such as applying main-sequence asymptotics to red giants, appears to have contributed significantly to our general understanding, which is very encouraging.

Nearly three decades ago several of my friends brought to my attention a review, written by another friend, of high-resolution spectroscopy, in which she condemned the use of the first $e$ in asteroseismology. I had coined the term after extensive discussion with Franz Deubner, who appreciates Attic Greek. My critic erroneously equated the Greek $ast\overline{e}r$ with $astron$, both of which mean star, with somewhat different connotations, and confused it with (the negation of) the suffix stereo (from $stereos$, meaning solid). My informants insisted that I publish a rebuttal. I was reluctant, but eventually succumbed to mounting pressure, and published a letter in $\mathit{{The \; Observatory}}$ justifying that letter $e$. Almost immediately afterwards I received a letter from Societas Oxometricalis of the University of Sydney awarding me the degree of DSc in Oxometry. After consulting Liddell and Scott (1889) I felt honoured for being recognised for having made a sharp point (oxometry, I inferred, being derived from $oxys$ meaning sharp or clever). Encountering Ron Bracewell during my next annual visit to Stanford University I told him the story, surmising that he might be interested, having come from Sydney himself. It transpired that Ron was actually a founding member of the Oxometrical Society, and knew of my award. He laughed heartily, exclaiming that my inference was quite wrong: “Oxometry is about ox”, he said, “and ox is bull: you are an expert in bull!”

## Electron Screening

My interest in the solar-neutrino problem naturally led me to investigate thermonuclear reaction rates. I had no intention of getting involved with nuclear cross sections, but the statistical mechanics seemed to be worth looking at. Central to the calculation is the electrostatic barrier penetration probability of two colliding nuclei, which is influenced by screening due to relatively fast electrons. Long before, Ed Salpeter (1954) had evaluated by Debye–Hückel theory the electron-density distribution around an isolated nucleus imbedded in an otherwise statistically uniform high-temperature, non-relativistic electron sea. Thence, he computed the barrier-penetration probability of an incident bare nucleus for the purpose of evaluating the reaction rate. Surely it would be better to consider the penetration of a screened nucleus, thought I. Together with a new research student, Marcus Brüggen, we set out to do so. We calculated the screening cloud around a pair of nuclei; at the level of approximation that Ed had adopted it was simply the sum of the two spherically symmetric Debye clouds. Thence, we evaluated the free energy of the system to use as the potential $V$ in Schrödinger’s equation. Our *modus operandi* was what Jüri and I had adopted long ago for our waves-on-waves calculation: I wrote on the blackboard, now in the IoA lecture room, with Marcus commenting and catching my many errors. The board was some 12 metres across, sufficient to hold the entire calculation because I write quite compactly. We arrived at the ratio of two complicated several-term algebraic expressions. I then set to dividing one into the other in the manner of arithmetic long division, and ended up with a simple result. “How did you know to do that?” asked an astounded Marcus. How could I possibly have seen from their mathematical structure that the denominator would divide exactly into the numerator? The answer is that I could not. I am a physicist, and my intuition dictated the structure of the answer: the two expressions had to divide, provided that no error still lurked in their derivation. Marcus had seen to that. Yet I admit that it was with some trepidation that I carried out the exercise; I really wanted Marcus to learn an important lesson.

The outcome of the calculation was a surprise: Ed’s result was unchanged. That couldn’t possibly be an accident. There had to be a simple explanation. We found one. Although the target nucleus and its screening cloud both exert a force on both the incident nucleus and its screening cloud, because at our leading level of approximation the incident cloud remains symmetrically distributed about its nucleus, it exerts no net force on it, and therefore makes no contribution to the electrostatic interaction of the incident nucleus with the target. Of course it modifies the equation of state, but that was not within our realm of endeavour at the time. The outcome was quite obviously obvious.^24^

The following year a paper appeared in the *Astrophysical Journal* in which it is stated that the screening contribution to the nuclear interaction energy when both Debye–Hückel electron clouds are taken into account is 3/2 times that computed with only a single cloud. At first glance that might seem plausible. But because Marcus and I had studied the matter we happened to be acutely aware that it is wrong. The erroneous claim had arisen from a specious argument purporting that the electrostatic force on a screening cloud due to the other screened ion, evaluated in Ed’s linearised (spherical) approximation, is all transmitted directly to the nucleus it surrounds rather than to the surrounding electrons; in reality, unnoticed by the authors, it contributes to an exchange of heat between the screening cloud and the surrounding isothermal plasma as the distance between the nuclei varies. We wrote to the leading author explaining the error, and received an aggressive reply accusing us of being wrong without even addressing our argument. So perhaps our result was not obvious after all. Marcus and I sent a rebuttal to the *Astrophysical Journal*, which was sent to the second author of the original paper to referee. The report mimicked the leading author’s response to our letter, and demanded outright rejection with the threat of publishing an attack should his demand not be met. We responded with a more detailed exposure of the flaw in their reasoning, but the referee was intransigent. In the meanwhile Virginia Trimble, acting chief editor whilst Helmut Abt was indisposed, had kindly sent our paper (Brüggen and Gough, 1997) to Hugh DeWitt, an expert in the subject, for an opinion, and he agreed with us. That closed the matter. The threatened attack never appeared.

What was needed now was to calculate the polarisation of the screening clouds. The Debye theory appears as the leading term in an expansion in a small parameter, which leads to a screening cloud whose density diverges at the nuclei (assuming them to be of zero size), yet is integrable. The expansion is formally asymptotic: taking it to the next order leads to non-integrable divergence. In reality, that is prevented by Pauli exclusion, which is ignored in the classical Debye approach. So, Marcus and I undertook an approximate quantum calculation, adopting a confined-atom model, which required serious calculation on Marcus’s part (Brüggen and Gough, 2000). I don’t know if anyone has read it. However, I do know that Marcus’s PhD dissertation was read, for it was awarded the Keith Runcorn Thesis Prize by the Royal Astronomical Society in 1999.

Before leaving this topic, I mention that some years earlier there had been controversy over the use of screened potentials for calculating nuclear reaction rates. Strictly speaking, one should first calculate the quantum-barrier penetration probability of colliding nuclei perturbed by the presence of the neighbouring charged particles in the plasma, which I formally write as the operation ${\mathcal{A}}$, and then average the outcome, operation ℬ, over the statistical distribution of all the perturbing particles. What is actually calculated is ${\mathcal{AB}}$, which some argued yields a different result. Visiting the Harvard Physics Department in 1993 to deliver the Loeb Lectures, I discussed with Shelly Glashow and Sidney Coleman whether ${\mathcal{A}}$ and ℬ commute. The answer eluded us. Then, late in the evening of my last day, as I was preparing to depart for home, Sidney phoned me in my hotel to explain that they do. His explanation was long and esoteric, too difficult for me to grasp properly. However, after mulling over what little I could fathom, I came to realise in my simple physical way why he was right. As a consequence I realised that it is right to use screened energy levels of bound species in the chemical picture of the equation of state, which I describe below, notwithstanding spectroscopic evidence that energy levels appear to be unperturbed by surrounding charged particles.

## Equation of State

Werner Däppen (Figure 11), who spent a couple of years in Cambridge in the late 1970s, has worked extensively on studying equations of state for stellar physics (e.g. Nayfonov and Däppen, 1998; Nayfonov et al., 1999; Baturin et al., 2000). They fall into two categories. The first is derived from the so-called chemical picture in which the stellar material is considered to be an assembly of ions, atoms and molecules, together with photons, whose structures and spatial distributions are influenced by the presence of their neighbours, calculated in a piecemeal manner. The second is the physical picture, which is simply an ensemble of nuclei, electrons and photons, and possibly other elementary particles, whose distributions are evaluated by expansions (about some ideal distribution) of the governing statistical mechanical equations that ideally should produce bound species automatically. It might seem that the latter is more reliable, but the expansions are extremely complicated to carry out, and in any case may not converge. In practice, a hybrid approach is usually adopted (Perez et al., 2009; Däppen, 2010), adding to the physical expansion of hydrogen and helium the heavier chemical species. Central to the chemical picture is the evaluation of the partition functions for individual species, which requires evaluating a statistical sum over energy levels of internal states. Before Werner and I first met, I had assembled such contributions in the manner of Fontaine, Graboske Jr, and van Horn (1977), making analytical approximations to the (screened) hydrogen and helium partition functions of Rogers, Graboske, and Harwood (1970) and treating (relativistic) electrons similarly to Eggleton, Faulkner, and Flannery (1973), adjusted to ensure that Maxwell relations were satisfied precisely.^25^ That equation was used first in the early investigation of the sensitivity of five-minute oscillations to envelope structure that I carried out in collaboration with Gabrielle Berthomieu and colleagues (Berthomieu et al., 1980) during my sabbatical year in Nice. Of the issues that needed to be addressed was how to define ground states of individual species within the mixture; it seemed to me that one must ensure that marginally ionising electrons must all have the same energy, despite that not being the generally accepted norm. When Werner arrived in Cambridge we investigated with the help of Jørgen the consequences of various assumptions under solar conditions, but never established a consistent approach on which to agree.

**Figure 14:**
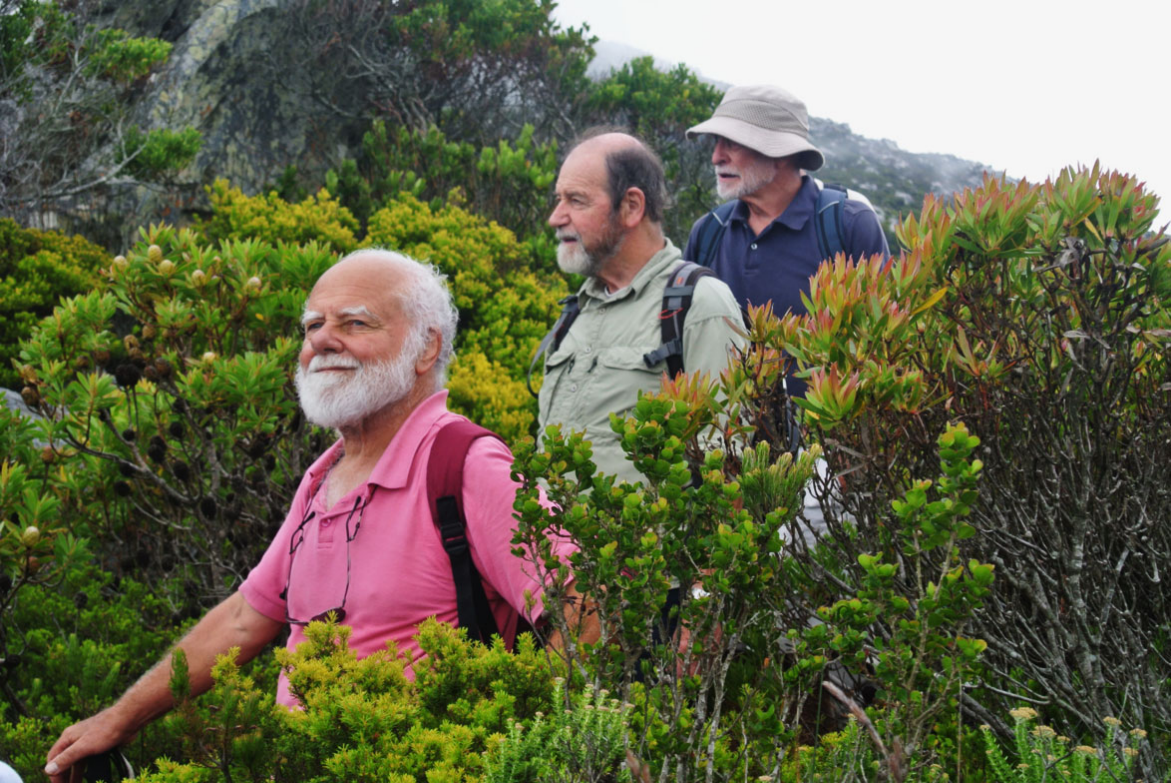
Walking in the foothills fynbos near Greyton, South Africa, with archaeologist Prof. Andy Smith and veterinary scientist Prof. John Nesbit (painting by Leonie Ash).

For many years Werner Däppen visited Cambridge during his spring break. Our most common discussions concerned testing the physical and chemical pictures against systems simple enough to analyse exactly, such as a single particle in a box. Unfortunately, although we could determine a precise effective equation of state, we were unable to design convincing analogues of the approximate chemical and physical pictures. In the last two decades Rosanne and I have escaped annually to South Africa to avoid boreal winter. We own a small house in Greyton, a village east of Cape Town, in which, fortuitously, the density of (mainly retired) university professors is comparable with that in Cambridge. The village is located on the wine route, nestled between two modest mountain ranges whose foothills boast the greatest floral diversity in the world. We have made many friends there, with whom we enjoy good food and wine, and pleasant mountain hikes (Figure 14). Sadly, our new life put paid to Werner’s visits. One cannot be in two places at once.

## Unhurried Investigations

I have already mentioned my protracted investigation with Takashi Sekii on error correlation, and my separate unfinished investigations with Radec Smolec and Günter Houdek and with Kumar Chitre and Bhooshan Paradkar on turbulent shear flow; also unfinished are collaborations with Takashi on a seismic signature of magnetic penetration of the tachocline and with Masao Takata on the acoustic radius of the Sun, all of them unhurried. The principal remaining unhurried investigations have been with graduate students who are learning research. I have enjoyed working with them enormously. By making it a teaching experience, overt progress is apparently unhurried. But I hope it is useful. I regard the activity as the duty of a supervisor, and I do not have my name on resulting publications unless I have made a substantial original contribution. As a result, I sometimes feel that our relationship had been incomplete, which makes me sad. I often resolve to engage in a subsequent collaboration, but that doesn’t always work; interests or opportunities can diverge too rapidly. I recognise that others think differently: indeed, I was once seriously shocked by a prospective employer who noticed that my student had no publications co-authored with me, and was concerned that the student might be difficult to get on with. The suspicion couldn’t have been farther from the truth. I had naively thought that publishing without me was a sure indication that the author had contributed almost everything, which should be to her advantage. It can also lead to slow completion. An extreme case was a student who persisted in not writing up his results; eventually I was moved to refusing even to speak to him until he presented me with at least one chapter of his thesis. It worked. Now he has the longest publication list of all my former students. I did not teach him how to achieve that.

## Dangers of Helioseismology

Lest you, my reader, believe astrophysics to be a peaceful activity, permit me to enlighten you (leaving aside the apparent danger described in Section 18). I start with a visit to India in 1984, which had been delayed by the assassination of Indira Gandhi, leaving great tension throughout the country. Whilst I was there I accompanied my erstwhile student, now the Raja of Mahmudabad, on a visit to a village near by, where he was to adjudicate on local disputes. We had to be accompanied by armed guards for our protection. Some years later I was lecturing at a summer school in Ahmedabad when dense smoke was seen rising from a serious racially incited riot in the city centre. The other lecturers flew directly home. However, I was scheduled to deliver a public lecture in Bombay (now Mumbai), where the riot was even more intense. Yet I was not going to default. As I journeyed through Bombay I passed many taxis that had been burnt by Hindus (taxis are driven by Muslims) killing the drivers. There was a curfew, and my lecture was cancelled because nobody was allowed to travel. So my coming to Bombay was in vain. In the early hours of the next morning I was due to be taken to the airport by a car from the British Council, but it didn’t arrive (I learned later that it had been intercepted by police, notwithstanding its diplomatic immunity). No public taxi was operating, but I managed to persuade the driver of a government car to moonlight. He demanded a handsome fee – of the order of several months of his normal income, I estimated – but I was desperate to leave. A nearby hotel opened its bank for me to draw the money. On the way we were stoned, but my driver assured me that we would survive because he was Hindu. How would an aggressor have known? The airport was packed with people hoping to leave the country on stand-bys released by no-shows, so my ‘taxi’ fee had been well spent.

In 2008, during a visit to the Tata Institute of Fundamental Research (TIFR) in Mumbai, my wife, Rosanne, and I dined in a restaurant with our good friends Kumar Chitre, now sadly deceased, and his wife Suvarna. From our taxi back to our guest house at TIFR in the south of the city, next to the Navy Nagar (cantonment), we heard a deafening explosion, and naturally thought it to be from manoeuvres off the coast. We were wrong. We were awakened in the middle of the night by a phone call from a worried daughter in England, who had just heard on the BBC news of the militant attack on Mumbai. A restaurant very near ours had been bombed, the occupants of a Jewish Outreach centre past which we had driven only seconds before the blast were being tortured and shot, and the two most prestigious hotels in Mumbai were on fire. We were confined to the Institute for five days, where we were safe. One positive effect for us was that the director of the Institute and his wife, whose home was next to a burning Taj Mahal hotel, joined us for safety, and the quality of the food improved from good to excellent.

In the first half of 1990 Jüri Toomre and I ran a workshop on helioseismology at the Institute of Theoretical Physics in Santa Barbara. It ended a week prematurely when a serious wildfire, the most destructive in California for some 40 years, destroyed nearly 450 houses (that record was surpassed in Oakland only a year later). In the early evening of what turned out to be our last day, Jüri and I had retired to the house he was renting for a glass or two of wine. The house overlooked a valley, on the other side of which we noticed two people start the fire, by accident, we thought. They were safely too far away to be of concern to us, so we believed. However, the combination of desiccated vegetation resulting from extreme drought and a strong wind sent burning embers flying across the valley over our heads. Our wives left in each of our cars, and we remained for as long as we dared hosing down the house and salvaging belongings into the house owners’ two cars, by which time we were now completely surrounded by fire. We each drove through burning scrub and arrived at the highway over which the fire had leapt leaving a wall of thick smoke. I shall never forget driving blind for 100 metres or so, gripping the steering wheel to hold a steady course hoping that no crashed car was in the way. I stayed up all that night watching the approach of the fire towards our rented house in which we hosted evacuees Jüri and his wife Linda, Michael Thompson, his wife Kate and their infirm neighbour who had forgotten her heart pills but had brought her canary – we were looking after the house owner’s two cats!

Wildfires in the fynbos surrounding Greyton are disturbingly common. I have helped fight them to prevent their penetration into the village, thereby protecting some houses and making new friends. Rosanne and I experienced two wildfires in Boulder too, but neither surrounded us completely; at another time we were impeded by a flash flood, but the source was too far away to be life threatening.

Before the fire in Santa Barbara an earthquake terrified some of the workshop participants; it was not our first: nearly 25 years earlier we had experienced one in Boulder, brought about by the local nuclear facility lubricating a fault in the underlying rock with radioactive waste. Neither was as exciting as those in Japan. My first was in hospital in Tokyo, where I had been abed for three months culminating with spinal surgery; before my discharge I was taken to a long corridor on the tenth floor to practise walking, fully expecting after having been horizontal for so long that I would feel dizzy. And so I was, for the white line painted down the middle of the floor appeared to wave. I was quickly pulled aside by my strapping physiotherapist so as to grasp the handrail on the wall; the line really was waving. I experienced several others thereafter: the most severe had occurred just as Rosanne and I were to leave Cambridge for Tokyo when our taxi driver suggested that we check the airline first. Sure enough, all flights to Japan had been cancelled in the wake of a tsunami that flooded the Fukushima power station. We flew several days later to an anxious community and several weaker shocks; we returned earlier than originally planned, on the last flight to Europe from an otherwise deserted Narita airport,^26^ closed in fear of radioactive fallout.

## Some Closing Remarks

I entered academic life believing it to be useful and satisfying. That has indeed been so. I have enjoyed teaching students immensely, at the same time having the opportunity to follow my immediate research interests, indulging in distractions by whatever exciting issues arise. With such a lifestyle, ongoing work is often interrupted. It has led to severe delays, I suspect to the annoyance of my patient collaborators. However, I hope that they judge my interactions with them to have compensated to some degree. To be sure, this policy yields fewer publications, but it doesn’t necessarily follow that scientific output is significantly diminished. I am extremely grateful for having had the privilege of being paid to do what I love.

Things have changed since I started, and I certainly do not advise young scientists to follow my example. I have tried not to replicate in research journals what I have already reported in conference proceedings, but, as Jørgen Christensen-Dalsgaard (2007) has complained on my behalf, conference proceedings are rarely acknowledged. Nowadays, it is necessary to scale the research-metrics ladder, because the volume of publications has risen so much that prospective employers and reviewers often find time merely to acknowledge the existence of papers rather than read them. Only after establishing oneself as an integral member of the academic community can one afford to follow one’s true desires. With regard to publication, I have noticed that those who publish every stage of their progress, each with fewer approximations than its predecessor, thereby requiring their readers to invest more time than should be necessary, now tend to be recognised by paper-counting reviewers as being greater authorities than their counterparts who, more efficiently, publish only their correct final result. Thus, prior to embarking on a research career, young scientists should first recognise why they have entered their field: principally to acquire recognition, or to advance knowledge most efficiently. Only then can they balance advertising their progress against the pursuit of greater understanding.

It has been very fulfilling interacting with so many wonderful people on my journey through life. I thank especially my research supervisor, Roger Tayler; my research students, Neil Balmforth, Guillaume Bascoul, Cecil Bloch, Marcus Brüggen, Heon-Young Chang, Jørgen Christensen-Dalsgaard, Ann Marie Cody, Alan Cooper, Margarida Cunha, Fisher Dilke, Julian Elliott, Nic Ellis, Dave Galloway, Pascale Garaud, John Gribbin, Chris Jordinson, Amoury Legait, Chris Jones, Doug Lin, David MacFarlane, Mohammed Nouri-Zonos, Colin Rosenthal, Sulaiman Kahn Raja of Mahmudabad, Peter Taylor, Michael Thompson, Will Thorne, Patrick Tooth and Toby Wood; my postdocs, Thierry Corbard, Werner Däppen, Noël Dolez, Günter Houdek, Sasha Kosovichev, Umin Lee, Ilidio Lopes, Bill Merryfield, Mike Montgomery, Cheri Morrow, Katie Mussack, Lida Nejad, Eva Novotny, Takashi Sekii, Masao Takata and Michael Thompson; my other senior collaborators, amongst whom are Norm Baker, Kumar Chitre, Tom Duvall, Wojtek Dziembowski, Phil Goode, Jack Harvey, Donald Lynden-Bell, Mike McIntyre, Phil Scherrer, Hiromoto Shibahashi, Ed Spiegel, Philip Stark, Jüri Toomre, Nigel Weiss, Sylvie Vauclair and Jean-Paul Zahn; this memoir is too short for me to have discussed the work of many of them. I thank Amanda Smith for her help with preparing the illustrations, my children and their spouses and a referee for commenting usefully on the manuscript. I am grateful to the American Astronomical Society for the award of the Hale Prize, and to the Royal Astronomical Society first for the Eddington Medal and subsequently for the Gold Medal. Most especially I am grateful to my loving wife, Rosanne, who has supported me throughout life; also my children, Kim, Heidi, Julian and Russell and their spouses Jaimie, Dom, Yurim and Tess, and my grandchildren, Marcus, Caitlin, Callum, Sam, Cara, Mahni and Juno all for being there.

## Footnotes

1 I never met my paternal grandfather, for he had been killed in the Fields of Flanders.

2 An eighth of a pound sterling: two-and-a-half weeks’ pocket money.

3 The roof has subsequently been repaired.

4 That is, one ranked in the First Class.

5 I didn’t yet realise that dedicated scientists enjoy discovery in whatever field they find themselves.

6 Again I paraphrase; as Rayleigh has pointed out, the criterion generalises to swirling flow – later, it transpired that in the criterion, which is commonly, and somewhat misleadingly, called the inflexion-point condition, the magnitude of the vorticity has to be a maximum (Føjrtoft, 1950; Balmforth and Morrison, 1999) – yet again I paraphrase; and I realised that more generally vorticity should be replaced by potential vorticity, by which I mean $\omega. \nabla s / \rho$, where $\omega$ is vorticity, $s$ is specific entropy and $\rho$ is density.

7 For example, at JILA in 1967 I was involved in an inconclusive experiment heating a suspension of iron filings in porridge immersed in a vertical magnetic field in an attempt to simulate a sunspot, notwithstanding a prescient warning by Harold Jeffreys (1926); and a few years later a physics student and I spent a summer in a refrigerator at the Scott Polar Research Institute investigating Mpemba’s claim (Mpemba and Osborne, 1969) that hot ice cream freezes faster than cold (and realising that we had to write our notes in pencil).

8 Recalling my admonition from the Cambridge computer service, which I mention later in Section 13, I had printed complete solutions for each value of $R$ with home-made digital line-printer graphics, which is why there was so much output.

9 The formula was not universally welcomed: the referee of the original publication wrote that it should not be published. Interestingly, his report was sent via Federal Express, who mistakenly delivered it to a remote location in the East Anglian fens owned by a psychiatrist knowing nothing about our subject yet was so interested as to read the entire discussion, then offering such a detailed description of the referee’s personality as to leave no doubt as to his identity.

10 after Newton, Raphson and Kantorovich

11 I wrote out the initial programme and Jørgen put it into effect, although I must point out that Jørgen asserts that I had made so many errors that what remains is barely recognisable.

12 These estimates came from model calibrations. I subsequently reported a direct estimate from an asymptotic evaluation of the sound speed (Gough, 1984a) that did not depend on a theoretical solar model, presenting also a (somewhat dubious) hint of limited overshooting or an asphericity of the convection-zone boundary (cf. Christensen-Dalsgaard, Gough, and Thompson, 1991; Christensen-Dalsgaard, Gough, and Knudstrup, 2018; Gough and Kosovichev, 1995).

13 Discussions of such representations applicable to solar-type stars are presented by, for example, Ledoux and Walraven (1958), Gough (1985), Vorontsov and Zharkov (1989), Gough and Thompson (1991), Gough (1993), Christensen-Dalsgaard (2002).

14 I must confess, however, that I did once fail completely to produce a manuscript: a substantial review for a conference organised by Christensen-Dalsgaard and Frandsen (1988). I had essentially finished writing it, except for a crucial section bringing together a variety of published theoretical results to draw a novel seismological conclusion. Unfortunately, one of the results was a serious misfit, and since it was purportedly error free, it appeared to have invalidated my argument. I corresponded with the author, and after considerable delay it transpired that his published result was erroneous; by then it was too late to submit my article.

15 From here on I call him John, to obviate the risk of confusion with Jack Harvey.

16 That is, someone familiar with telescopes, whom Jack would acknowledge as an observer.

17 Not to be confused with ‘John’ Miller.

18 An argument that I didn’t publish until a couple of decades later (Gough, 1990a).

19 The NBS prediction would have been one of them, had it been entered.

20 Masao Takata and I are trying to complete a seismological analysis to determine the acoustic radius and its variation (see, e.g. Takata and Gough, 2003).

21 A less approximate treatment in more restricted shearing flow (see Smolec, Houdek, and Gough, 2011) was almost completed some years earlier, but unfortunately our living in separate countries has impeded completion.

22 A work for large orchestra written by the Danish composer Poul Ruders describing the Sun as being like a gong in a sandstorm.

23 This was before the days of computer presentations. In fact, I don’t know how our presentation could have been made as effective with only modern technology.

24 Why hadn’t Ed mentioned it in his paper? I wrote to him accusing him of having known this result all along. He modestly denied it. Surely it was at least in his subconscious. Scientists can be divided into two types: those whose subconscious knowledge is usually right, and those who are usually wrong. Ed is of the first type. Nevertheless, it seemed that this now obvious result was hardly worth publishing.

25 It seemed obvious to take due account in the equation of state of electrons being relativistic, yet it took Elliott and Kosovichev (1998) to expose this apparently unobvious obvious property.

26 That was one of two ‘escapes’ through an empty airport: the second was from South Africa in 2020 in the wake of the Covid-19 outbreak; all commercial flights were cancelled, but eventually we were lucky to be assigned a repatriation flight from Cape Town Airport, staffed for our flight – aside from air-traffic control and a solitary young lady seeing that passengers had passports – entirely by British-Airways and British-High-Commission personnel.
